# Supplementary material for: Susceptibility status of Aedes aegypti (Diptera: Culicidae) to public health insecticides in Southern Afar Region, Ethiopia
Source: PLoS One. 2024 Aug 23;19(8):e0309335. doi: 10.1371/journal.pone.0309335 (PMC11343450; doi:10.1371/journal.pone.0309335)
Supplement: S1 Table — (DOCX) [file pone.0309335.s001.docx]

| Study sites | Total female *Aedes aegypti* exposed | Time in minutes | Observed *Aedes aegypti* knocked-down in response to the insecticides | | | |
| --- | --- | --- | --- | --- | --- | --- |
|  |  |  | Deltamethrin | Alpha-cyprmethrin | Permethrin | Controls |
| Awash Sebat | 100 | 10 | 14 | 13 | 58 | 0 |
|  |  | 20 | 54 | 87 | 90 | 0 |
|  |  | 30 | 95 | 97 | 98 | 0 |
|  |  | 40 | 98 | 100 | 99 | 0 |
|  |  | 50 | 100 | 100 | 100 | 0 |
|  |  | 60 | 100 | 100 | 100 | 0 |
| Awash Arba | 100 | 10 | 23 | 13 | 81 | 0 |
|  |  | 20 | 97 | 93 | 94 | 0 |
|  |  | 30 | 100 | 100 | 98 | 0 |
|  |  | 40 | 100 | 100 | 100 | 0 |
|  |  | 50 | 100 | 100 | 100 | 0 |
|  |  | 60 | 100 | 100 | 100 | 0 |
| Werer | 100 | 10 | 13 | 15 | 20 | 0 |
|  |  | 20 | 59 | 56 | 65 | 0 |
|  |  | 30 | 97 | 94 | 98 | 0 |
|  |  | 40 | 100 | 100 | 100 | 0 |
|  |  | 50 | 100 | 100 | 100 | 0 |
|  |  | 60 | 100 | 100 | 100 | 0 |

**Note:** The experiments were carried out on 20 non-blood fed female *Aedes aegypti* with five replicates (total of 100) for each test in the exposure tubes and 20 *Ae. aegypti* with 2 replicates (total of 40) in the control tubes. In case of Alpha-cyprmethrin in Awash Arba town, the average knocked-downs of *Ae. aegypti* of the two tests were taken.
