# Supplementary material for: Susceptibility status of Aedes aegypti (Diptera: Culicidae) to public health insecticides in Southern Afar Region, Ethiopia
Source: PLoS One. 2024 Aug 23;19(8):e0309335. doi: 10.1371/journal.pone.0309335 (PMC11343450; doi:10.1371/journal.pone.0309335)
Supplement: S2 Table — (DOCX) [file pone.0309335.s002.docx]

| S. No. | Study sites | Insecticide | | No. of dead and alive *Ae. aegypti* after 24 hrs of recovery times | | |
| --- | --- | --- | --- | --- | --- | --- |
| 1 | Awash Sebat | 0.05% Deltamethrin | Total female *Aedes aegypti* used in the test (exposure and control tubes) | Dead(n) | Alive(n) | Remark |
|  |  |  |  |  |  | R; Replicate  C; Control |
|  |  | R1 | 20 | 20 | 0 |  |
|  |  | R2 | 20 | 20 | 0 |  |
|  |  | R3 | 20 | 20 | 0 |  |
|  |  | R4 | 20 | 20 | 0 |  |
|  |  | R5 | 20 | 20 | 0 |  |
|  |  | C1 | 20 | 0 | 20 |  |
|  |  | C2 | 20 | 0 | 20 |  |
|  |  | 0.75% Permethrin |  |  |  |  |
|  |  | R1 | 20 | 20 | 0 |  |
|  |  | R2 | 20 | 20 | 0 |  |
|  |  | R3 | 20 | 20 | 0 |  |
|  |  | R4 | 20 | 20 | 0 |  |
|  |  | R5 | 20 | 20 | 0 |  |
|  |  | C1 | 20 | 0 | 20 |  |
|  |  | C2 | 20 | 0 | 20 |  |
|  |  | 0.05% Alpha-cypermethrin |  |  |  |  |
|  |  | R1 | 20 | 20 | 0 |  |
|  |  | R2 | 20 | 20 | 0 |  |
|  |  | R3 | 20 | 20 | 0 |  |
|  |  | R4 | 20 | 20 | 0 |  |
|  |  | R5 | 20 | 20 | 0 |  |
|  |  | C1 | 20 | 0 | 20 |  |
|  |  | C2 | 20 | 0 | 20 |  |
|  |  | 0.25% Pirimiphos-methyl |  |  |  |  |
|  |  | R1 | 20 | 20 | 0 |  |
|  |  | R2 | 20 | 20 | 0 |  |
|  |  | R3 | 20 | 20 | 0 |  |
|  |  | R4 | 20 | 20 | 0 |  |
|  |  | R5 | 20 | 20 | 0 |  |
|  |  | C1 | 20 | 0 | 20 |  |
|  |  | C2 | 20 | 0 | 20 |  |
|  |  | 0.1% Bendiocarb |  |  |  |  |
|  |  | R1 | 20 | 16 | 4 |  |
|  |  | R2 | 20 | 18 | 2 |  |
|  |  | R3 | 20 | 17 | 3 |  |
|  |  | R4 | 20 | 18 | 2 |  |
|  |  | R5 | 20 | 19 | 1 |  |
|  |  | C1 | 20 | 0 | 20 |  |
|  |  | C2 | 20 | 0 | 20 |  |
|  |  | 0.1% Propoxur |  |  |  |  |
|  |  | R1 | 20 | 16 | 4 |  |
|  |  | R2 | 20 | 18 | 2 |  |
|  |  | R3 | 20 | 17 | 3 |  |
|  |  | R4 | 20 | 18 | 2 |  |
|  |  | R5 | 20 | 18 | 2 |  |
|  |  | C1 | 20 | 0 | 20 |  |
|  |  | C2 | 20 | 0 | 20 |  |
| 2 | Awash Arba | 0.05% Deltamethrin |  |  |  |  |
|  |  | R1 | 20 | 20 | 0 |  |
|  |  | R2 | 20 | 20 | 0 |  |
|  |  | R3 | 20 | 20 | 0 |  |
|  |  | R4 | 20 | 20 | 0 |  |
|  |  | R5 | 20 | 20 | 0 |  |
|  |  | C1 | 20 | 0 | 20 |  |
|  |  | C2 | 20 | 0 | 20 |  |
|  |  | 0.75% Permethrin |  |  |  |  |
|  |  | R1 | 20 | 20 | 0 |  |
|  |  | R2 | 20 | 20 | 0 |  |
|  |  | R3 | 20 | 20 | 0 |  |
|  |  | R4 | 20 | 20 | 0 |  |
|  |  | R5 | 20 | 20 | 0 |  |
|  |  | C1 | 20 | 0 | 20 |  |
|  |  | C2 | 20 | 0 | 20 |  |
|  |  | 0.05% Alpha-cypermethrin (1) |  |  |  |  |
|  |  | R1 | 20 | 18 | 2 |  |
|  |  | R2 | 20 | 20 | 0 |  |
|  |  | R3 | 20 | 18 | 2 |  |
|  |  | R4 | 20 | 20 | 0 |  |
|  |  | R5 | 20 | 20 | 0 |  |
|  |  | C1 | 20 | 0 | 20 |  |
|  |  | C2 | 40 | 0 | 20 |  |
|  |  | 0.05% Alpha-cypermethrin (2) |  |  |  |  |
|  |  | R1 | 20 | 18 | 2 |  |
|  |  | R2 | 20 | 17 | 3 |  |
|  |  | R3 | 20 | 20 | 0 |  |
|  |  | R4 | 20 | 19 | 1 |  |
|  |  | R5 | 20 | 20 | 0 |  |
|  |  | C1 | 20 | 0 | 20 |  |
|  |  | C2 | 20 | 0 | 20 |  |
|  |  | 0.25% Pirimiphos-methyl |  |  |  |  |
|  |  | R1 | 20 | 20 | 0 |  |
|  |  | R2 | 20 | 20 | 0 |  |
|  |  | R3 | 20 | 20 | 0 |  |
|  |  | R4 | 20 | 20 | 0 |  |
|  |  | R5 | 20 | 20 | 0 |  |
|  |  | C1 | 20 | 0 | 20 |  |
|  |  | C2 | 20 | 0 | 20 |  |
|  |  | 0.1% Bendiocarb |  |  |  |  |
|  |  | R1 | 20 | 17 | 3 |  |
|  |  | R2 | 20 | 18 | 2 |  |
|  |  | R3 | 20 | 17 | 3 |  |
|  |  | R4 | 20 | 18 | 2 |  |
|  |  | R5 | 20 | 18 | 2 |  |
|  |  | C1 | 20 | 0 | 20 |  |
|  |  | C2 | 20 | 0 | 20 |  |
|  |  | 0.1% Propoxur |  |  |  |  |
|  |  | R1 | 20 | 16 | 4 |  |
|  |  | R2 | 20 | 19 | 1 |  |
|  |  | R3 | 20 | 18 | 2 |  |
|  |  | R4 | 20 | 16 | 4 |  |
|  |  | R5 | 20 | 18 | 2 |  |
|  |  | C1 | 20 | 0 | 20 |  |
|  |  | C2 | 20 | 0 | 20 |  |
| 3 | Werer | 0.05% Deltamethrin |  |  |  |  |
|  |  | R1 | 20 | 20 | 0 |  |
|  |  | R2 | 20 | 20 | 0 |  |
|  |  | R3 | 20 | 20 | 0 |  |
|  |  | R4 | 20 | 20 | 0 |  |
|  |  | R5 | 20 | 20 | 0 |  |
|  |  | C1 | 20 | 0 | 20 |  |
|  |  | C2 | 20 | 0 | 20 |  |
|  |  | 0.75% Permethrin |  |  |  |  |
|  |  | R1 | 20 | 20 | 0 |  |
|  |  | R2 | 20 | 20 | 0 |  |
|  |  | R3 | 20 | 20 | 0 |  |
|  |  | R4 | 20 | 20 | 0 |  |
|  |  | R5 | 20 | 20 | 0 |  |
|  |  | C1 | 20 | 0 | 20 |  |
|  |  | C2 | 20 | 0 | 20 |  |
|  |  | 0.05% Alpha-cypermethrin |  |  |  |  |
|  |  | R1 | 20 | 20 | 0 |  |
|  |  | R2 | 20 | 19 | 1 |  |
|  |  | R3 | 20 | 20 | 0 |  |
|  |  | R4 | 20 | 20 | 0 |  |
|  |  | R5 | 20 | 20 | 0 |  |
|  |  | C1 | 20 | 0 | 20 |  |
|  |  | C2 | 20 | 0 | 20 |  |
|  |  | 0.25% Pirimiphos-methyl |  |  |  |  |
|  |  | R1 | 20 | 20 | 0 |  |
|  |  | R2 | 20 | 20 | 0 |  |
|  |  | R3 | 20 | 20 | 0 |  |
|  |  | R4 | 20 | 20 | 0 |  |
|  |  | R5 | 20 | 20 | 0 |  |
|  |  | C1 | 20 | 0 | 20 |  |
|  |  | C2 | 20 | 0 | 20 |  |
|  |  | 0.1 Propoxur |  |  |  |  |
|  |  | R1 | 20 | 17 | 3 |  |
|  |  | R2 | 20 | 19 | 1 |  |
|  |  | R3 | 20 | 17 | 3 |  |
|  |  | R4 | 20 | 17 | 3 |  |
|  |  | R5 | 20 | 17 | 3 |  |
|  |  | C1 | 20 | 0 | 20 |  |
|  |  | C2 | 20 | 0 | 20 |  |

**Note:** No *Ae. aegypti* mortalities were recorded in the control tubes at 24 hrs recovery time. In case of Alpha-cyprmethrin in Awash Arba town, the average percentage mortalities of *Ae. aegypti* of the two tests were taken.
