# Supplementary material for: Susceptibility status of Aedes aegypti (Diptera: Culicidae) to public health insecticides in Southern Afar Region, Ethiopia
Source: PLoS One. 2024 Aug 23;19(8):e0309335. doi: 10.1371/journal.pone.0309335 (PMC11343450; doi:10.1371/journal.pone.0309335)
Supplement: S3 Table — (DOCX) [file pone.0309335.s003.docx]

| S.No. | Sites | Latitude (N) | Longitude(E) | Elevation (in meter) | Remark |
| --- | --- | --- | --- | --- | --- |
| 1 | Awash Sebat | 8.98389 | 40.159223 | 925 |  |
| 2 | Awash Arba | 9.126098 | 40.170989 | 842 |  |
| 3 | Werer | 9.33453 | 40.181385 | 729 |  |
