# Supplementary material for: Susceptibility status of Aedes aegypti (Diptera: Culicidae) to public health insecticides in Southern Afar Region, Ethiopia
Source: PLoS One. 2024 Aug 23;19(8):e0309335. doi: 10.1371/journal.pone.0309335 (PMC11343450; doi:10.1371/journal.pone.0309335)
Supplement: Attachment — Submitted filename: PONE-D-24-03915.pdf [file pone.0309335.s004.pdf]

The authors conducted a study to evaluate the susceptibility status of *Aedes aegypti* (Diptera: Culicidae) to public health insecticides in the Southern Afar region, Ethiopia. The findings revealed that populations of *Ae. aegypti* in the study area showed phenotypic resistance to carbamates such as propoxur and bendiocarb. However, they were susceptible to certain pyrethroids, including permethrin, alphacypermethrin, and deltamethrin, as well as the organophosphate insecticide primiphos-methyl. The study provides compelling evidence of an emergence of insecticide resistance within Aedes aegypti populations in the country. While the study provides valuable insights into the emergence of insecticide resistance within *Aedes aegypti* populations, the writing of the paper requires revision to enhance its overall quality.

Major comments

1. What criteria underlie the selection of insecticides in this study, specifically the inclusion of three pyrethroids and two carbamates? Additionally, are these insecticides currently in use for public health purposes?
2. According to the WHO guideline cited in the methodology section, the recommended discriminating doses for susceptibility tests of Aedes mosquitoes using pyrethroids are 0.25% Permethrin, 0.03% alpha-cypermethrin, and 0.03% deltamethrin. However, it appears that different discriminating doses of pyrethroids were used for the study. Why it is different?
3. Why only phenotypic resistance studied? Was there a specific reason for not investigating the resistance mechanisms at play in the mosquito populations?
4. How did you identified the Aedes mosquitoes used for the susceptibility tests were all *Ae. Aegypti*?
5. How and why was the association between pyrethroid resistance in the study area and agricultural practices established?

Minor comments

1. Are *Ae aegypti* and *Ae bromeliae* the only two species these the only two Aedes species existing in the country? For example *Ae. vittaus* and *Ae. africanus* also exist in the country.
2. Why the Knockdown effect of permethrin and alpha-cypermethrin not elaborated like that of deltamethrin?
3. The vertical axis maximum value should not exceed 100 (Fig 2). This works for all graphs
4. The susceptibility test of alphacypermethri in Awash Arba town repeated or not?
5. Why is integrated vector control strategies recommended without supporting data from the study findings?
6. How are pyrethroids currently utilized in the control of vectors?

Suggestions

Suggestions and comments are provided in the main document
