# Supplementary material for: Susceptibility status of Aedes aegypti (Diptera: Culicidae) to public health insecticides in Southern Afar Region, Ethiopia
Source: PLoS One. 2024 Aug 23;19(8):e0309335. doi: 10.1371/journal.pone.0309335 (PMC11343450; doi:10.1371/journal.pone.0309335)
Supplement: Attachment — Submitted filename: PONE-D-24-03915_MS.pdf [file pone.0309335.s005.pdf]

PLOS ONE

Susceptibility status of Aedes aegypti (Diptera: Culicidae) to public health insecticides in Southern Afar region, Ethiopia

--Manuscript Draft--

**Manuscript Number:**	PONE-D-24-03915
**Article Type:**	Research Article
**Full Title:**	Susceptibility status of Aedes aegypti (Diptera: Culicidae) to public health insecticides in Southern Afar region, Ethiopia
**Short Title:**	Susceptibility status of Aedes aegypti to public health insecticides
**Corresponding Author:**	Mohammed Seid<br>Addis Ababa University<br>Addis Ababa, ETHIOPIA
**Keywords:**	Aedes aegypti; Afar; Ethiopia; insecticides; susceptibility
**Abstract:**	Background Mosquito-borne viral diseases such as dengue fever, chikungunya and yellow fever have been documented in Ethiopia since the 1960s. However, efficacy of public health insecticides against Aedes aegypti that transmit these viruses remain poorly understood in the country particularly in the Afar region. Thus, the aim of this study was to assess the susceptibility status of Ae. aegypti to public health insecticides. Methodology Larvae and pupae of Aedes mosquitoes were collected from the Awash Arba, Awash Sebat and Werer towns of the Afar Regional State of Ethiopia during July to October 2022, brought to Aklilu Lemma Institute of Pathobiology, Addis Ababa University Insectary and reared to adults under standard conditions. Non-blood fed 3 to 5 days old female Aedes aegypti were exposed to standard WHO papers impregnated with public health insecticides that belonged to the pyrethroids, carbamates and organophosphate classes following the World Health Organization standard guidelines. Knockdown rates (KDT) were noted at 10 min interval until one hour. After an hour, the exposed females were transferred into holding tubes and kept for 24 h. Mortality was recorded after 24 hours. Result Aedes aegypti from Awash Arba showed high KDT95 (26.5 minutes) to deltamethrin compared to the Ae. aegypti from the other two towns. Mortalities of Ae. aegypti exposed to propoxur was 87% in all the study towns. Similarly, 88% of Ae. aegypti mortality was recorded when treated with bendiocarb in Awash Sebat and Awash Arba towns. Suspected resistance of Ae. aegypti (95% mortality) exposed to alphacypermethrin were recorded from Awash Arba town. However, Ae. aegypti collected from all the three sites were observed to be susceptible to delthamethrin, permethrin, and pirimiphos methyl. All the Ae. aegypti in the control group showed 0% mortalities. Conclusion Aedes aegypti was resistant to bendiocarb and propoxur, and possibly resistant to alphacypermethrin. On the other hand, it was susceptible to delthamethrin, permethrin and pirimiphos methylcontrol. Thus, delthamethrin, permethrin and pirimiphos methyl can be used in the control of adult Ae. aegypti in the Afar Region of Ethiopia.
**Order of Authors:**	Mohammed Seid
	Esayas Aklilu
	Abebe Animut
**Additional Information:**	
**Question**	**Response**
**Financial Disclosure**<br><br>Enter a financial disclosure statement that describes the sources of funding for the work included in this submission. Review the submission guidelines for detailed requirements. View published research	The author(s) received no specific funding for this work.

Powered by Editorial Manager® and ProduXion Manager® from Aries Systems Corporation

articles from *PLOS ONE* for specific examples.<br><br>This statement is required for submission and **will appear in the published article** if the submission is accepted. Please make sure it is accurate.<br><br>**Funded studies**<br>Enter a statement with the following details:<br>• Initials of the authors who received each award<br>• Grant numbers awarded to each author<br>• The full name of each funder<br>• URL of each funder website<br>• Did the sponsors or funders play any role in the study design, data collection and analysis, decision to publish, or preparation of the manuscript?<br><br>Did you receive funding for this work?	
**Competing Interests**<br><br>Use the instructions below to enter a competing interest statement for this submission. On behalf of all authors, disclose any competing interests that could be perceived to bias this work—acknowledging all financial support and any other relevant financial or non-financial competing interests.<br><br>This statement is required for submission and **will appear in the published article** if the submission is accepted. Please make sure it is accurate and that any funding sources listed in your Funding Information later in the submission form are also declared in your Financial Disclosure statement.<br><br>View published research articles from *PLOS ONE* for specific examples.	The authors declared that there is no conflict of interest.

Powered by Editorial Manager® and ProduXion Manager® from Aries Systems Corporation

**NO authors have competing interests**<br><br>Enter: *The authors have declared that no competing interests exist.*<br><br>**Authors with competing interests**<br><br>Enter competing interest details beginning with this statement:<br><br>*I have read the journal's policy and the authors of this manuscript have the following competing interests: [insert competing interests here]*<br><br>* typeset	
**Ethics Statement**<br><br>Enter an ethics statement for this submission. This statement is required if the study involved:<br><br>• Human participants<br>• Human specimens or tissue<br>• Vertebrate animals or cephalopods<br>• Vertebrate embryos or tissues<br>• Field research<br><br>Write "N/A" if the submission does not require an ethics statement.<br><br>General guidance is provided below. Consult the submission guidelines for detailed instructions. **Make sure that all information entered here is included in the Methods section of the manuscript.**	"N/A"

**Format for specific study types**<br><br>**Human Subject Research (involving human participants and/or tissue)**<br>• Give the name of the institutional review board or ethics committee that approved the study<br>• Include the approval number and/or a statement indicating approval of this research<br>• Indicate the form of consent obtained (written/oral) or the reason that consent was not obtained (e.g. the data were analyzed anonymously)<br><br>**Animal Research (involving vertebrate animals, embryos or tissues)**<br>• Provide the name of the Institutional Animal Care and Use Committee (IACUC) or other relevant ethics board that reviewed the study protocol, and indicate whether they approved this research or granted a formal waiver of ethical approval<br>• Include an approval number if one was obtained<br>• If the study involved *non-human primates*, add *additional details* about animal welfare and steps taken to ameliorate suffering<br>• If anesthesia, euthanasia, or any kind of animal sacrifice is part of the study, include briefly which substances and/or methods were applied<br><br>**Field Research**<br><br>Include the following details if this study involves the collection of plant, animal, or other materials from a natural setting:<br>• Field permit number<br>• Name of the institution or relevant body that granted permission	
**Data Availability**<br><br>Authors are required to make all data underlying the findings described fully available, without restriction, and from the time of publication. PLOS allows rare exceptions to address legal and ethical concerns. See the PLOS Data Policy and FAQ for detailed information.	No - some restrictions will apply

Powered by Editorial Manager® and ProduXion Manager® from Aries Systems Corporation

A Data Availability Statement describing where the data can be found is required at submission. Your answers to this question constitute the Data Availability Statement and **will be published in the article**, if accepted.<br><br>**Important:** Stating 'data available on request from the author' is not sufficient. If your data are only available upon request, select 'No' for the first question and explain your exceptional situation in the text box.<br><br>Do the authors confirm that all data underlying the findings described in their manuscript are fully available without restriction?	
**Describe where the data may be found in full sentences. If you are copying our sample text, replace any instances of XXX with the appropriate details.**<br><br>• If the data are **held or will be held in a public repository**, include URLs, accession numbers or DOIs. If this information will only be available after acceptance, indicate this by ticking the box below. For example: *All XXX files are available from the XXX database (accession number(s) XXX, XXX.).*<br>• If the data are all contained **within the manuscript and/or Supporting Information files**, enter the following: *All relevant data are within the manuscript and its Supporting Information files.*<br>• If neither of these applies but you are able to provide **details of access elsewhere**, with or without limitations, please do so. For example:<br><br>*Data cannot be shared publicly because of [XXX]. Data are available from the XXX Institutional Data Access / Ethics Committee (contact via XXX) for researchers who meet the criteria for access to confidential data.*<br><br>*The data underlying the results presented in the study are available from (include the name of the third party*	The data supports the findings are included in the article and additional data are found in the corresponding author upon request.

and contact information or URL).<br>• This text is appropriate if the data are owned by a third party and authors do not have permission to share the data.<br><br>* typeset	
Additional data availability information:	

Manuscript Click here to access/download;Manuscript;Manuscript.doc

Susceptibility status of *Aedes aegypti* (Diptera: Culicidae) to public health insecticides in Southern Afar region, Ethiopia

Mohammed Seid[1, 2*], Esayas Aklilu[1], Abebe Animut[1]

[1] Aklilu Lemma Institute of Pathobiology, Addis Ababa University, Addis Ababa, Ethiopia

[2] Department of Biology, College of Natural and Computational Science, Mattu University, Ethiopia

*Corresponding author

Email: mohammed.seid.legas@gmail.com (MS)

Abstract

Background: Mosquito-borne viral diseases such as dengue fever, chikungunya and yellow fever have been documented in Ethiopia since the 1960s. However, efficacy of public health insecticides against *Aedes aegypti* that transmit these viruses remain poorly understood in the country particularly in the Afar region. Thus, the aim of this study was to assess the susceptibility status of *Ae. aegypti* to public health insecticides.

Methodology: Larvae and pupae of *Aedes* mosquitoes were collected from the Awash Arba, Awash Sebat and Werer towns of the Afar Regional State of Ethiopia during July to October 2022, brought to Aklilu Lemma Institute of Pathobiology, Addis Ababa University Insectary and reared to adults under standard conditions. Non-blood fed 3 to 5 days old female *Aedes aegypti* were exposed to insecticides that belonged to the pyrethroids, carbamates and organophosphate classes following the World Health Organization standard guidelines. Knockdown rates (KDT) were noted at 10 min interval until one hour. Mortality was recorded after 24 hours.

Result: *Aedes aegypti* from Awash Arba showed high KDT_{95} (26.5 minutes) to deltamethrin compared to the *Aedes aegypti* from other study sites.. Mortalities of *Ae. aegypti* exposed to propoxur was 87% in all the study towns. Similarly, 88% of *Ae. aegypti* mortality was recorded when treated with bendiocarb in Awash Sebat and Awash Arba towns. Suspected re sistance of *Ae. aegypti* (95% mortality) exposed to alphacypermethrin were recorded from Awash Arba town. However, *Ae. aegypti* collected from all the three sites were observed to be susceptible to delthamethrin, permethrin, and pirimiphos methyl.

Conclusion: *Aedes aegypti* was resistant to bendiocarb and propoxur, and possibly resistant to alphacypermethrin. On the other hand, it was susceptible to delthamethrin, permethrin and

pirimiphos methylcontrol. Thus, delthamethrin, permethrin and pirimiphos methyl can be used in the control of adult *Ae. aegypti* in the Afar Region of Ethiopia.

Key words: *Aedes aegypti*, Afar, Ethiopia, Insecticides, Susceptibility

Authors Summary

Insecticide based mosquito control is an immediate and effective response during mosquito-borne viral diseases outbreak. However, mosquitos' resistance to insecticides adversely affect insecticide based control programmes. Insecticide resistance and possible resistance were observed in *Ae. aegypti* in the Southern Afar Regional State Ethiopia. On the other hand, *Ae. aegypti* was susceptible for delthamethrin, permethrin and pirimiphos methyl. Therefore, the Ethiopian Ministry of Health and Afar Regional Health Bureau can use these insecticides for the control of *Ae. aegypti*.

Introduction

The ever increasing outbreaks of arboviral diseases drew public attention in several countries of the world [1]. The East African countries including Kenya, Somalia, Djibouti, Eritrea and Ethiopia are epicentres of outbreaks of arboviral diseases such as dengue, yellow fever and chikungunya [2-4]. In Ethiopia, yellow fever caused about 30,000 deaths from over 200,000 infections during the 1960 and 1962 [5]. Recently, there have been yellow fever outbreaks in South Omo [6], dengue fever outbreaks in Dire Dawa administrative city, Somali Regional State, Gewane and Amibara districts of Afar Regional State [7, 8]. In addition, chikungunya outbreaks were also reported in Dire Dawa city, Afar and Somali Regional States [9, 10]. All these outbreaks with potentially unrecognized and/or under reported deaths owing to the highly limited laboratory facilities in the country.

Mosquitoes transmit over 90% of the viral pathogens through their bites among which *Aedes aegypti* is the leading vector followed by *Ae. albopictus*[11, 12]. *Ae. aegypti* is anthropophilic in its behaviour and occurs in close proximity to human habitations [13, 14]. *Ae. aegypti* has two morphological subspecies (ecotypes) the domestic *Ae. aegypti aegypti* and sylvan *Ae .aegypti formosus* [15, 16]. In Ethiopia, *Ae. aegypti* and *Ae. bromeliae* are implicated as the potential vectors of viral diseases [6, 8, 17, 18].

In the absence of effective therapeutic drugs and vaccines to treat and control mosquito borne viral diseases, preventions largely depend on vector control tools mainly public health insecticides [13]. The pyrethroids such as deltamethrin, alpha-cypermethrin, permethrin and

organophosphates primiphos methyl and Fenitrothion are among the public health insecticides used against *Aedes* mosquitoes [19]. However, the potential resistance to the insecticides may adversely affect implementation of the control strategy in endemic areas [20]. Mosquitoes could resist insecticides through their inherent ability to detoxify and avoid contacts either behaviourally or physiologically[21].

Evidences from many African countries indicate that the *Anopheles* and *Aedes* mosquitoes developed resistance to insecticides [22-24]. For instance in Ethiopia, malaria transmitting *Anopheles arabiensis* has developed resistance to insecticides in several areas of the country including in the Amibara district of the Afar Regional State [24, 25]. The causes for the resistance could be frequent use of the chemicals in indoor residual spraying and long-lasting insecticidal treated nets for the control of mosquito borne diseases in the area [20] or in argro-pesticides [26]. However, the susceptibility status of *Aedes* mosquitoes to insecticides currently used is lacking in the country.

Recently, the Ethiopian Public Health Institute prepared arboviral disease vectors, surveillance and control guideline in 2021 through chemical and environmental management methods. Vector control by chemical method such as fumigation, repellents and residual treatments were implemented in Dire Dawa administrative city and Somalia Regional State during dengue fever and chikungunya outbreaks (EPHI, 2021 unpublished). Unfortunately, there is a scarcity of evidences on the efficacy of the insecticides for the control of virus transmitting *Aedes* species in the Southern Afar Region in the presence of increased reports of viral diseases incidence and prevalence. Therefore, assessing the effectiveness of the existing public health insecticides to control *Aedes* species is crucial to strengthen the arboviral diseases prevention and control programs in the country. This inspired us to generate base line data in relation to the applicability of insecticide control tools in the prevention and control of virus transmitting *Aedes* mosquitoes in the area. Therefore, the aim of the study was to assess the susceptibility status of *Ae. aegypti* to public health insecticides in the Southern part of the Afar Regional State of Ethiopia.

Materials and Methods

Study sites

The study was carried out in Awash Arba, Were and Awash Sebat towns, Gabi-Rasu Zone (Zone 3), of Afar Regional State, Ethiopia. The Awash Arba and Werer towns are located about 250 km to the North-east of Addis Ababa at 9.33453^{o}N latitude, 40.181385^{o}E longitude

and an altitude range of 720 metres above sea level (masl) to 1100 masl (Central statistics Agency, 2019 unpublished). Awash Arba and Werer sites were selected from Amibara district which are about 30 km distance far apart. Agro-ecologically, these two towns are semi-arid with temperature ranging from 25 °C to 35 °C, and average annual rainfall of 530 mm. The communities livelihoods are characterized as pastoralist and agro-pastoralist in which they mainly obtained income on livestock and from livestock and other agricultural crop production [27].

Awash Sebat town administration is located 214 km North-east of Addis Ababa at 8.98810^{o}N latitude and 40.163936^{o}E longitude (Central statistics Agency, 2019 unpublished). Its altitude ranges from 820masl to 1120 masl and the temperature varies from 22.6^{o}C to 30.6^{o}C. The town and its surroundings receive a mean annual rainfall of 606.6mm. The study towns were selected in collaboration with the local district health professionals and on the basis of the recent outbreak reports of dengue fever and chikungunya (Awash Sebat town and Amibara district health bureaus, 2022 unpublished).

The study areas are found in the middle Awash Valley, where infectious disease such as malaria, chikungunya and dengue fever are common [28]. Considerable attention has been given by the regional government to achieve better health in the region including the three towns [29]. The region has hospitals, health centres, clinics and health posts in different zones and districts. There are one hospital, one clinic and five health centers and few private clinics. Insecticide spraying is practiced for the control of malaria and arboviral diseases in these towns [25]. Similarly, agro-chemicals and agro-pesticides are used since maize and sorghum productions are common in these areas which is used to improve agricultural crop production [30].

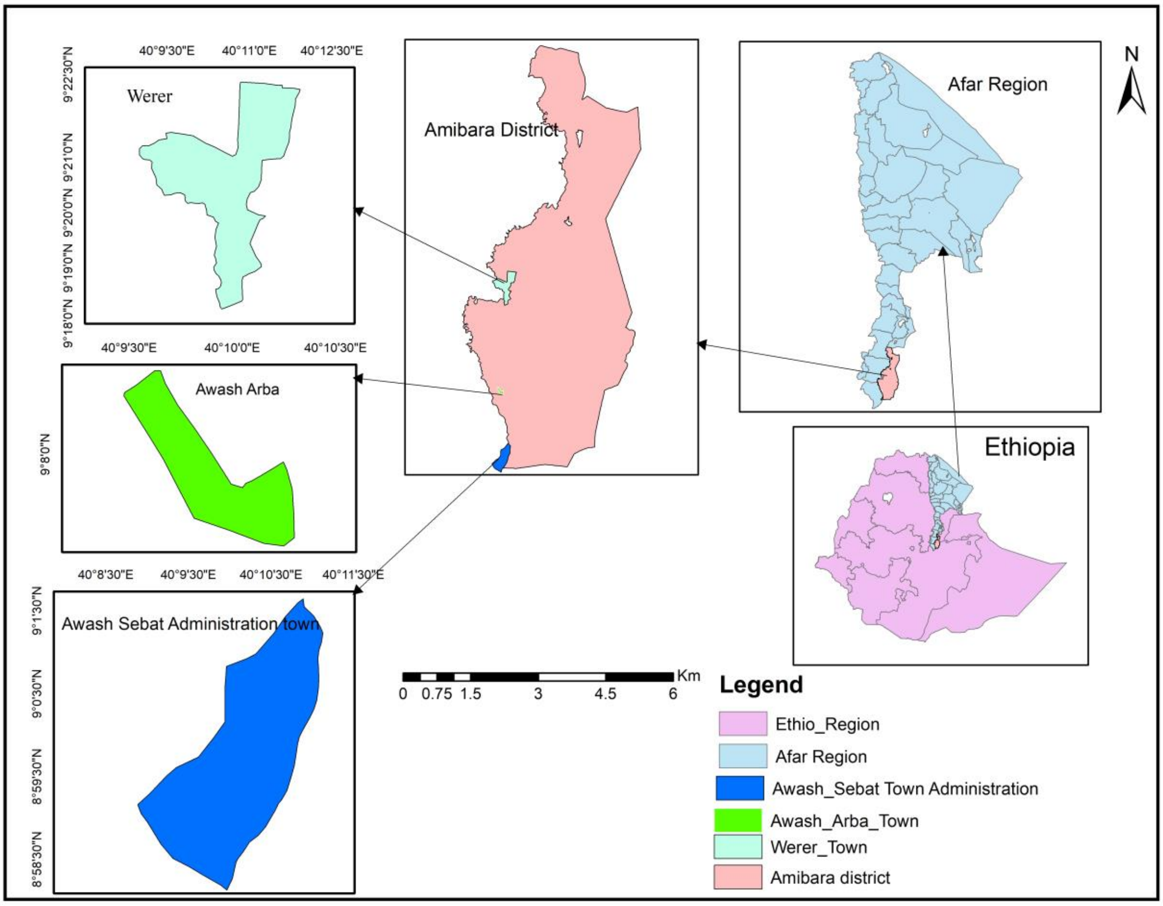

Figure 1. Map of Awash Arba, Awash Sebat and Werer Towns, Southern Afar, Ethiopia (Source: Extracted from Ethio_GIS, 2023)

Larval collection and rearing

Larvae and pupae of *Aedes* species were collected from different habitats including tyres, water storage drums, discarded plastics and metallic containers from July to October, 2022. The collected larvae were brought to the insectary of Aklilu Lemma Institute of Pathobiology, Addis Ababa University, Ethiopia and reared to adults under standard laboratory conditions. The larvae were fed tetra cichlid floating fish food (®/TM/©2019 Germany). The emergence of larvae in to pupae was checked each morning and pupae were collected in glass flasks and transferred in to (30cm x 30cm x 30cm) netted cages. Emerged adult *Aedes aegypti* were kept at 27 ± 2 °C temperature, 75 ± 10% relative humidity and fed on 10% sucrose solution until the bioassay tests. Emergent adults were identified to species using morphological taxonomic keys for *Aedes* species [31].

Insecticide susceptibility

Prior to insecticide susceptibility tests, sugar fed female *Ae. aegypti* were starved for 2hrs in order to prevent them from having high weight which leads them to be knocked down. Non-blood fed, 3-5 days old female *Ae. aegypti* were tested for their susceptibility to insecticides following the World Health Organization insecticide testing procedures(16). They were exposed to pyrethroids (0.05% delta-methrin, 0.05% alpha cypermethrin, 0.75% permethrin) , carbamates (0.1% bendio carb,and 0.1% propoxur:) and organophosphates (0.25% pirimip hos methyl) which are commonly used for preventing disease transmitting mosquitoes [19]. In each test, 20 female *Ae. aegypti* were exposed to each five treatment replicates and two control test tubes making a total of 140 mosquitoes in a single test. The exposed mosquitoes were observed for 60 minutes at an interval of 10 minutes to observe their knockdown. After an hour, the exposed females were transferred into holding tubes and kept for 24 h at 27.0 ± 2.0 °C and 75 ± 10% relative humidity and fed on 10% sugar solution. Mortality was recorded after 24 hours. In case of Werer town the susceptibility status of *Ae. aegypti* to bendiocarb was not conducted due to insufficient mosquito samples.

Ethical Issues

The study protocol obtained ethical approval from the institutional review board (IRB) of Aklilu Lemma Institute of Pathobiology, Addis Ababa University, Ethiopia (June 13, 2022; ALIPB IRB/80/2022). Written permission letters were obtained from the district health bureau and health centers of the towns. The households were informed about the objective of the study and their agreements were sought prior to the larvae/pupae collection.

Data analysis

Mortality was determined on the basis of the dead and alive mosquitoes after the 24 hours recovery period. Susceptible were those with a mortality in the range of 98-100%, suspected for resistance were with a mortality 90-98% and resistant if mortality was <90% (34). The knockdown effect of pyrethroids against the *Ae. aegypti* were recorded during the 60 minutes exposure at an interval of 10 minutes from the start. Probit analysis was used to determine mean duration at which 50% (KD50) and 95% (KD95) of the exposed *Ae. aegypti* mosquitoes were knocked down. Overall adult mortalities and knockdown times were computed using SPSS Software version 20.

Results

Knockdown effect of insecticides against *Aedes aegypti*

Knockdown time of *Ae. aegypti* exposed to insecticides was depicted in Table 1**.** The knock down effect of 50% (KDT$_{50}$) of *Ae. aegypti* exposed of deltamethrin ranged from 11.75 to 22.5 minutes whereas KDT$_{95}$ of the insecticide ranged from 18.75 to 26.5 minutes. *Ae. aegypti* from the Awash Arba showed high KDT$_{95}$ (26.5 minutes) to deltamethrin as compared to the *Ae. aegypti* from the other sites (Table 1). Overall, the KDT$_{50}$ and KDT$_{95}$ of *Ae. aegypti* varied among *Ae. aegypti* in knockdown to insecticides across the study localities.

Table 1 Knockdown times of 50% and 95% of *Ae. Aegypti* exposed to commonly used insecticides in three localities from July to October 2022 in Southern Afar region Ethiopia

Insecticides	Localities	KDT50±SE	KDT95±SE
Deltamethrin	Awash sebat	22.5± 4.16	26.5±4.5
	Awash Arba	11.75±10.4	17.5±0.5
	Werer	12.75±6.84	18.75±0.13
Permethrin	Awash sebat	11.25±3.4	15.25
	Awash Arba	9±1.0	15.5±4.4
	Werer	12.25±2.59	15.5±0.5
Alphacypermethrin	Awash sebat	14±3.0	18±10.5
	Awash Arba	14.75±4.12	16±1.0
	Werer	15.75±0.81	16.5±0.5

SE, standard error; KDT50, time taken for 50% of the tested *Ae. aegypti* to be knocked-down; KDT95 time taken for 95% of the tested *Ae. aegypti* to be knocked-down

[illegible]sceptibility status of *Aedes aegypti* to insecticides

The percentage mortalities of *Ae. aegypti* mosquito population against insecticides was presented in figures (fig. 2-4). Accordingly, *Ae. aegypti* mosquitoes showed resistance to bendiocarb and propoxur insecticides in which the percentage mortalities were recorded to be 88% and 87% respectively in Awash Sebat town. *Ae. aegypti* mortalities to deltamethrin, permethrin alphacypermthrin and pirimiphos methyl were 100% in Awash sebat town (Fig. 2). All mosquitoes in the control group showed 0% mortality in all study sites.

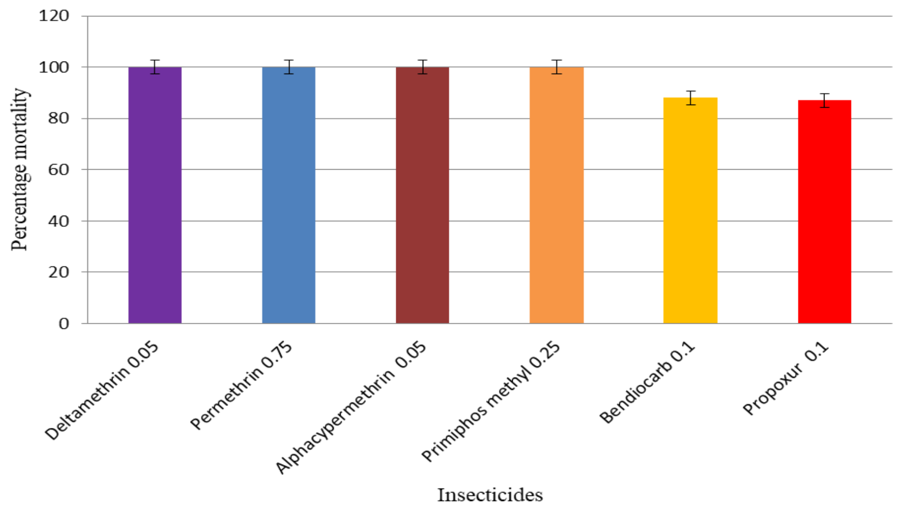

Figure 2. Insecticide susceptibility status of *Ae. aegypti* from Awash Sebat town, Southern Afar regional State Ethiopia from July to October 2022 (Error bars represent 95% confidence intervals).

In addition, *Ae. aegypti* mosquitoes were also observed resistance to bendiocarb and propoxur from Awash Arba. Similarly *Ae. aegypti* mosquitoes were observed to be suspected resistant to alphacypermethrin in which the mortality of *Ae. aegypti* mosquitoes due to alphacypermethrin was 95% (Fig 3). 100% of *Ae. aegypti* mosquitoes mortalities were recorded in Awash Arba to deltamethrin, permethrin and pirimiphos methyl.

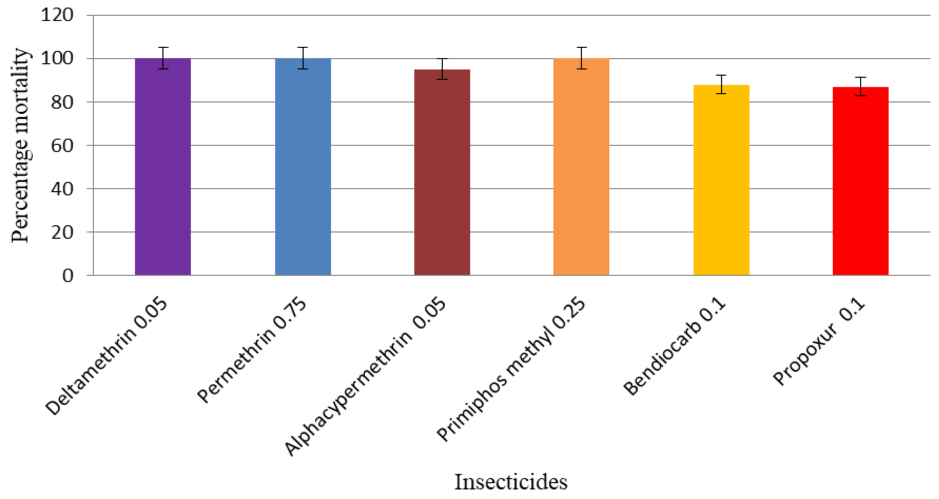

Figure 3. Insecticide susceptibility status of *Ae. aegypti* population Awash Arba town, Southern Afar region Ethiopia from July to October 2022 (Error bars represent 95% confidence intervals).

About 87%and 99% mortalities were observed among the *Ae. aegypti* mosquitoes from Werer town when exposed to propoxur and alphacypermethrin insecticides respectively (fig.

4). However, 100% mortalities of *Ae. aegypti* from this site were recorded to deltamethrin, permethrin and pirimiphos methyl.

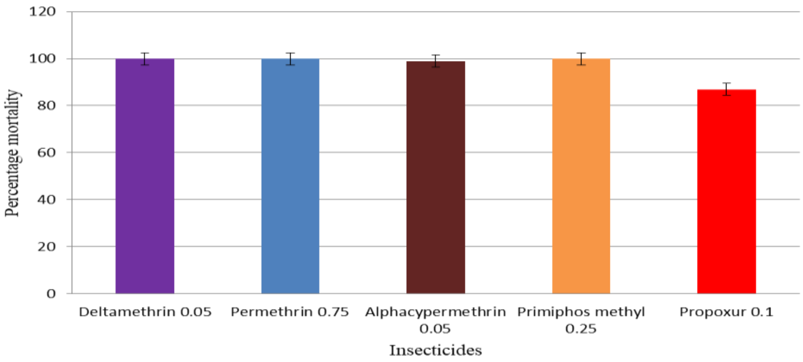

Figure 4. Insecticide susceptibility status of Adult *Ae.aegypti* population in Werer town, Southern Afar region Ethiopia from July to October 2022 (Error bars represent 95% confidence intervals).

Discussion

Recent emergence and re-emergence of arboviral diseases in the Eastern and the North Eastern parts of the country including Dire Dawa administrative city, Somali Regional State and Amibara and Gewane districts of Afar regional State calls an alarm to confirm the efficacy of mortality of the public health insecticides in Afar regional State and elsewhere in the country [7, 32]. However, the control of viral disease transmitting mosquitos' vector is poorly investigated and needs detailed research. Major focus was given to the malaria vector and there is the scarcity of research findings conducted to control *Aedes* mosquitoes either chemically, biologically or other means of vector control in the country [33]. As far as our knowledge concern, this study was first in its kind to report the susceptibility status of *Ae. aegypti* to public health chemical insecticides in the southern Afar regional State and there are limited data to compare our findings to others in the country.

Ae. aegypti is generally considered as exophillic, exophagic and day time bitters. They usually feed on human and other vertebrate hosts as described in previous studies [34, 35]. However, they may tend to be endophilic and endophagic in which they rest and fed indoors [36]. This makes controlling of the *Aedes* mosquitoes and the viral diseases they transmitted more difficult [37]. As a result, different intervention tools have been adopted to control these mosquitoes including indoor residual insecticide spraying and insecticide impregnated long lasting insecticide treated nets in addition to environmental management by removing *Aedes* breeding habitats.

The present study revealed that the *Ae. aegypti* KDT_{50} and KDT_{95} for deltamethrin, ranged from 11.25-22.5 minutes and 15.25-26.5 minutes recorded in the study towns. This knock down results were highest as compared to other pyrethroids in the study areas. On the other hand, *Ae. aegypti* showed the lowest KDT_{50} to permethrin as compared to delthamethrin and alphacypermethrin in Awah town. Relatively high tolerance of deltamethrin observed in the present study areas may be attributable to its frequent use in the insecticide treated nets. However, the findings observed in the present study were lower than results reported in Sudan, Tanzania and Nigeria [38-40].

Ae. aegypti populations varied in their response to public health insecticides. In the present study, *Ae. aegypti* was resistant to bendiocarb where the mortality rates were less than 90%. *Ae. aegypti* resistance to bendiocarb was also reported in a previous study in Tanzania [39]. However, these finding were in contrary to the finding reported from Sudan, Nigeria and Cambodia [38, 40, 41]. The variation in the findings from the present study may be due to ecological factors in addition to the concentration difference of the insecticides.
~~In the present study *Ae. aegypti* mortally against propoxur insecticide was assessed~~. It was observed that *Ae. aegypti* mortality when exposed to propoxur showed ~~insecticide~~ resistance in all the study towns. Similar findings were reported in Thailand where the efficacy of *Ae. aegypti* mortality to propoxur was 83% which indicated the phenotypic resistance of *Ae. aegypti* to propxur in this area[42].

Mosquitoes as a disease vector emerge insecticide resistance to commonly used public health insecticides possibly one of the following ways. First, frequent uses of insecticide chemicals such as insecticide treated bed net and indoor residual insecticide spraying to control mosquito borne diseases may lead to mosquitoes to develop insecticide resistance gene [43]. On the other hand, now a days agro-pesticide are now widely used to improve agricultural crop production. Unfortunately however, agro-pesticides induce insecticides resistance selection pressure in different ways. For instance, insecticides used to control mosquitoes can counteract to agro-pesticides used which have the same mode of action may drive a section pressure to chemical based insecticide control tools [26]. Similarly, agro-pesticide used in agriculture may ease insecticide tolerance as the pesticides washed to mosquito breeding habitats which confer resistance mechanism at the adult stages [44].

Ethiopia has long history in utilizing pesticides to increase agricultural production and to improve human health since 1964 [45, 46]. Thus, the emergence of insecticide resistance in bendiocarb and propoxur insecticides in the present study could potentially arise from the

frequent exposer of the mosquito to carbamate insecticides in agriculture used as an agro-pesticides or herbicides.

The pyerethroids such as deltamethrin are chemical insecticides commonly used to control malaria and other mosquito borne diseases in Ethiopia. In present study, the pyrethroid alphacypermethrin showed less than 97% of mortality of adult *Ae. aegypti* population in the Awash Arba town which revealed the emergence of possible resistance in *Ae. aegypti* mosquito population in this area. The emergence of possible resistance of *Ae. aegypti* mosquitoes in Awash Arba may be the frequent use of pyrethroids in the insecticide treated nets to control malaria in this area and agro-pesticides as discussed above. This may lead failer to vector control interventions programs in the study area and elsewhere in the country. Similar findings were reported in Ghana[22].

In the present study, complete susceptibility of *Ae. aegypti* mosquito populations were recorded to the organophosphate chemical insecticide i.e. pirimiphos methyl in all the study areas. Likewise, the study conducted in Cambodia[41] reported similar findings in that the adult *Ae. aegypti* populations were completely susceptible for the pirimiphos methyl even in its lower concentration than in the present study. However, this finding was in contrary to the findings reported in Nigeria [40]. Deltamethrin and perimethrin of the pyrethroid insecticides are also showed 100% mortality to adult *Ae.aegypti* mosquito population in the study areas. These findings were in contrary to the findings reported in Malaysia [47] where resistance phenotypes were observed in permethrin and deltamethrin. However, Similar findings was reported in Tanzania [39] and in Peru [48] where deltamethrin showed greater than 99% of mortality in adult *Ae. aegypti*. Thus, this findings could serve as a base line to the Ethiopian ministry of health and the Afar region health bureau to implement the chemical interventions particularly the pyrethroids deltametrin, permethrin and the organophosphate pirimiphos methyl either in the form of aerial spray or surface treatment to reduce the burden of arboviral diseases during diseases outbreak in different parts of the country.

Conclusion

The present study reported that *Ae. aegypti* population showed insecticide resistance to bendiocarb and propoxur and possible resistance to alphacypermethrin and complete susceptibility to deltamethrin, perimethrin and pirimiphos methyl insecticides. From the finding of the present study, the following points would be forwarded. Further study should be inspired on insecticide resistance of *Aedes* mosquitoes in the study areas and elsewhere in

Ethiopia including molecular aspects to mark resistance pattern. In addition, integrated vector control strategies should be developed in the study areas and other arboviral endemic areas of Ethiopia. Finally, the organophosphate insecticide pirimiphos methyl can be used in addition to deltamethrin and permethrin for the control of adult *Ae. aegypti* mosquitoes in Ethiopia.

Acknowledgement

The authors would like to acknowledge Aklilu Lemma Institute of Pathobiology Addis Ababa University, Ethiopia for providing us field and laboratory materials and chemicals. We also like to gratitude the vector biology and control units staffs, Mr. Wosen Sisay for his technical assistance in the insectary. Finally, we appreciate all the communities and data collectors from the localities of Awash sebat town, Awash Arba and Werer for their collaboration during the study period.

Author contributions

Conceptualization: Mohammed Seid, Esayas Aklilu and Abebe Animut

Data curation: Mohammed Seid

Formal Analysis: Mohammed Seid

Investigations: Mohammed Seid, Esayas Aklilu and Abebe Animut

Methodology: Mohammed Seid, Esayas Aklilu and Abebe Animut

Supper-vision: Mohammed Seid, Esayas Aklilu and Abebe Animut

Validation: Mohammed Seid, Esayas Aklilu and Abebe Animut

Visualization: Mohammed Seid, Esayas Aklilu and Abebe Animut

Writing original draft: Mohammed Seid

Writing, reviewing and editing: Mohammed Seid, Esayas Aklilu and Abebe Animut

Conflict of interest

The authors declared that there is no conflict of interest.

Funding

The study was financially supported by Addis Ababa University and Ministry of Higher Education Ethiopia

References

1. Braack, L., et al., *Mosquito-borne arboviruses of African origin: review of key viruses and vectors.* Parasites & vectors, 2018. **11**(1): p. 1-26.

2. Langat, S.K., et al., *Origin and evolution of dengue virus type 2 causing outbreaks in Kenya: Evidence of circulation of two cosmopolitan genotype lineages.* Virus Evolution, 2020. **6**(1): p. veaa026.
3. Gutu, M.A., et al., *Another dengue fever outbreak in Eastern Ethiopia—An emerging public health threat.* PLoS Neglected Tropical Diseases, 2021. **15**(1): p. e0008992.
4. Usman, A., et al., *Dengue fever outbreaks in Eritrea, 2005–2015.* Global health research and policy, 2016. **1**(1): p. 1-8.
5. Sérié, C., et al., *Studies on yellow fever in Ethiopia. 6. Epidemiologic study.* Bulletin of the World Health Organization, 1968. **38**(6): p. 879-884.
6. Lilay, A., et al., *Reemergence of yellow fever in Ethiopia after 50 years, 2013: epidemiological and entomological investigations.* BMC infectious diseases, 2017. **17**(1): p. 1-6.
7. Woyessa, A.B., et al., *The first acute febrile illness investigation associated with dengue fever in Ethiopia, 2013: a descriptive analysis.* The Ethiopian Journal of Health Development, 2014. **28**(3).
8. Mekuriaw, W., et al., *Epidemiological, Entomological, and Climatological Investigation of the 2019 Dengue Fever Outbreak in Gewane District, Afar Region, North-East Ethiopia.* Insects, 2022. **13**(11): p. 1066.
9. Geleta, D., et al., *Epidemiological Description of Chikungunya Virus Outbreak in Dire Dawa Administrative City, Western Ethiopia, 2019.* Int J Clin Exp Med Sci, 2020. **6**(3): p. 41.
10. Alayu, M., et al., *Risk factors for Chikungunya outbreak in Kebridhar City, Somali Ethiopia, 2019. Unmatched case-control study.* bioRxiv, 2020: p. 2020.01. 21.913673.
11. Gubler, D.J., *Dengue, urbanization and globalization: the unholy trinity of the 21st century.* Tropical medicine and health, 2011. **39**(4SUPPLEMENT): p. S3-S11.
12. Li, Y., et al., *Urbanization increases Aedes albopictus larval habitats and accelerates mosquito development and survivorship.* PLoS neglected tropical diseases, 2014. **8**(11): p. e3301.
13. WHO, et al., *Dengue: guidelines for diagnosis, treatment, prevention and control*. 2009: World Health Organization.
14. Powell, J.R., *Mosquito-borne human viral diseases: why Aedes aegypti?* The American journal of tropical medicine and hygiene, 2018. **98**(6): p. 1563.

15. Mattingly, P., *Taxonomy of Aedes aegypti and related species.* Bulletin of the World Health Organization, 1967. **36**(4): p. 552.
16. Rose, N.H., et al., *Climate and urbanization drive mosquito preference for humans.* Current Biology, 2020. **30**(18): p. 3570-3579. e6.
17. Getachew, D., et al., *Breeding sites of Aedes aegypti: potential dengue vectors in Dire Dawa, East Ethiopia.* Interdisciplinary perspectives on infectious diseases, 2015. **2015**.
18. Ferede, G., et al., *Distribution and larval breeding habitats of Aedes mosquito species in residential areas of northwest Ethiopia.* Epidemiology and health, 2018. **40**.
19. WHO, *Monitoring and managing insecticide resistance in Aedes mosquito populations: interim guidance for entomologists.* 2016.
20. Moyes, C.L., et al., *Contemporary status of insecticide resistance in the major Aedes vectors of arboviruses infecting humans.* PLoS neglected tropical diseases, 2017. **11**(7): p. e0005625.
21. WHO, *Global plan for insecticide resistance management in malaria vectors*. 2012: World Health Organization.
22. Owusu-Asenso, C.M., et al., *Spatiotemporal distribution and insecticide resistance status of Aedes aegypti in Ghana.* Parasites & Vectors, 2022. **15**(1): p. 1-14.
23. Mathias, L., et al., *Habitat productivity and pyrethroid susceptibility status of Aedes aegypti mosquitoes in Dar es Salaam, Tanzania.* Infectious Diseases of Poverty, 2017. **6**(1): p. 1-10.
24. Kenea, O., et al., *Impact of combining indoor residual spraying and long-lasting insecticidal nets on Anopheles arabiensis in Ethiopia: results from a cluster randomized controlled trial.* Malaria Journal, 2019. **18**(1): p. 1-11.
25. Messenger, L.A., et al., *Insecticide resistance in Anopheles arabiensis from Ethiopia (2012–2016): a nationwide study for insecticide resistance monitoring.* Malaria journal, 2017. **16**(1): p. 1-14.
26. Nkya, T.E., et al., *Impact of environment on mosquito response to pyrethroid insecticides: facts, evidences and prospects.* Insect biochemistry and molecular biology, 2013. **43**(4): p. 407-416.
27. Mekuyie, M., A. Jordaan, and Y. Melka, *Understanding resilience of pastoralists to climate change and variability in the Southern Afar Region, Ethiopia.* Climate Risk Management, 2018. **20**: p. 64-77.

28. Gebru, H. and J. Ahmed, *Water quality assessments as implication to human health: a case study in awash sebat and its surrounding areas, Afar, Ethiopia.* International Journal of Earth Sciences and Engineering, 2016. **9**(5): p. 1999-2008.
29. Goshu, D., et al., *Socioeconomic Development in Afar Region: Achievements, Gaps and Priorities.* 2021.
30. Sileshi, G., A. Zemedkun, and S. Nurhussien, *Assessment of the pesticide use, practice and hazards on cotton growing farmers in the Awash Valley Ethiopia.* AgriRxiv, 2021(2021): p. 20210139206.
31. Rueda, L.M., *Pictorial keys for the identification of mosquitoes (Diptera: Culicidae) associated with dengue virus transmission*. 2004, Walter Reed Army Inst Of Research Washington Dc Department Of Entomology.
32. Ahmed, Y.M. and A.A. Salah, *Epidemiology of dengue fever in Ethiopian Somali region: retrospective health facility based study.* Cent Afr J Public Health, 2016. **2**(2): p. 51-6.
33. Waldetensai, A., et al., *Aedes mosquito responses to control interventions against the Chikungunya outbreak of Dire Dawa, Eastern Ethiopia.* International Journal of Tropical Insect Science, 2021: p. 1-10.
34. Diouf, B., et al., *Resting behavior of blood-fed females and host feeding preferences of Aedes aegypti (Diptera: Culicidae) morphological forms in Senegal.* Journal of Medical Entomology, 2021. **58**(6): p. 2467-2473.
35. Ponlawat, A. and L.C. Harrington, *Blood feeding patterns of Aedes aegypti and Aedes albopictus in Thailand.* Journal of medical entomology, 2005. **42**(5): p. 844-849.
36. Diallo, D. and M. Diallo, *Resting behavior of Aedes aegypti in southeastern Senegal.* Parasites & Vectors, 2020. **13**(1): p. 1-7.
37. Bartlow, A.W., et al., *Forecasting zoonotic infectious disease response to climate change: mosquito vectors and a changing environment.* Veterinary sciences, 2019. **6**(2): p. 40.
38. Husham, A., M. Abdalmagid, and M. Brair, *Status susceptibility of dengue vector; Aedes aegypti to different groups of insecticides in Port Sudan City-Red Sea State.* Sudanese Journal of Public Health, 2010. **5**(4): p. 199-202.
39. Kahamba, N.F., et al., *Habitat characteristics and insecticide susceptibility of Aedes aegypti in the Ifakara area, south-eastern Tanzania.* Parasites & Vectors, 2020. **13**(1): p. 1-15.

40. Ukpai, O.M. and C.M. Ekedo, *Insecticide susceptibility status of Aedes aegypti in umudike, Ikwuano lga Abia State, Nigeria.* Animal Research International, 2018. **15**(3).
41. Boyer, S., et al., *Monitoring insecticide resistance of adult and larval Aedes aegypti (Diptera: Culicidae) in Phnom Penh, Cambodia.* Parasites & Vectors, 2022. **15**(1): p. 1-7.
42. Pethuan, S., et al., *Biochemical studies of insecticide resistance in Aedes (Stegomyia) aegypti and Aedes (Stegomyia) albopictus (Diptera: Culicidae) in Thailand.* Trop Biomed, 2007. **24**(1): p. 7-15.
43. Barbosa, S., et al., *Modelling the impact of insecticide-based control interventions on the evolution of insecticide resistance and disease transmission.* Parasites & vectors, 2018. **11**: p. 1-21.
44. Poupardin, R., et al., *Cross-induction of detoxification genes by environmental xenobiotics and insecticides in the mosquito Aedes aegypti: impact on larval tolerance to chemical insecticides.* Insect biochemistry and molecular biology, 2008. **38**(5): p. 540-551.
45. Mengistie, B., *Policy-practice nexus: pesticide registration, distribution and use in Ethiopia.* SM J. Environ. Toxicol, 2016. **2**(1): p. 1006-1019.
46. Negatu, B., S. Dugassa, and Y. Mekonnen, *Environmental and health risks of pesticide use in Ethiopia.* Journal of Health Pollution, 2021. **11**(30): p. 210601.
47. Ishak, I.H., et al., *Contrasting patterns of insecticide resistance and knockdown resistance (kdr) in the dengue vectors Aedes aegypti and Aedes albopictus from Malaysia.* Parasites & vectors, 2015. **8**(1): p. 1-13.
48. Pinto, J., et al., *Susceptibility to insecticides and resistance mechanisms in three populations of Aedes aegypti from Peru.* Parasites & vectors, 2019. **12**(1): p. 1-11.
