# Supplementary material for: Susceptibility status of Aedes aegypti (Diptera: Culicidae) to public health insecticides in Southern Afar Region, Ethiopia
Source: PLoS One. 2024 Aug 23;19(8):e0309335. doi: 10.1371/journal.pone.0309335 (PMC11343450; doi:10.1371/journal.pone.0309335)
Supplement: Attachment — Submitted filename: PONE-D-24-03915_R1_reviewer_2.pdf [file pone.0309335.s007.pdf]

PLOS ONE

Susceptibility status of Aedes aegypti (Diptera: Culicidae) to public health insecticides in Southern Afar region, Ethiopia

--Manuscript Draft--

**Manuscript Number:**	PONE-D-24-03915R1
**Article Type:**	Research Article
**Full Title:**	Susceptibility status of Aedes aegypti (Diptera: Culicidae) to public health insecticides in Southern Afar region, Ethiopia
**Short Title:**	Susceptibility status of Aedes aegypti to public health insecticides
**Corresponding Author:**	Mohammed Seid<br>Addis Ababa University<br>Addis Ababa, ETHIOPIA
**Keywords:**	Aedes aegypti; Afar; Ethiopia; insecticides; Susceptibility
**Abstract:**	Mosquito-borne viral diseases such as dengue fever, chikungunya and yellow fever have been documented in Ethiopia since the 1960s. However, efficacy of public health insecticides against Aedes aegypti that transmit these viruses remain poorly understood in the country particularly in the Afar region. Thus, the aim of this study was to assess the<br>susceptibility status of Ae. aegypti to deltamethrin, permethrin, alpha-cypermethrin, piri miphos-methyl, bendiocarb and propoxur insecticides. Larvae and pupae of Aedes mosquitoes were collected from the Awash Arba, Awash Sebat and Werer towns of the Afar Regional State of Ethiopia during July to October 2022, brought to Aklilu Lemma Institute of Pathobiology, insectary and reared to adults. Non-blood fed 3 to 5 days old female Ae. aegypti were exposed to pyrethroids, carbamates and organophosphate insecticides following the World Health Organization standard guidelines. Knockdown rates were noted at 10 min interval until one hour. The mortality in mosquitoes was recorded 24 hours after 60 minutes<br>of exposure. Mortalities of Ae. aegypti exposed to propoxur were 87% in all the study t owns. Similarly, 88% of Ae. aegypti mortality was recorded when treated with bendioca rb in Awash Sebat and Awash Arba towns. Suspected resistance of Ae. aegypti (95% mortality) exposed to alpha-cypermethrin were recorded from Awash Arba town. However, Ae. aegypti collected from all the three sites were observed to be susceptible to deltamethrin, permethrin, and pirimiphos-methyl. Aedes aegypti was resistant to 0.1% bendiocarb and 0.1% propoxur, and possibly resistant to 0.05% alpha-cypermethrin. On the other hand, it was susceptible to 0.05% deltamethrin, 0.75% permethrin and 0.25% pirimiphos-methyl. Thus, deltamethrin, permethrin and pirimiphos-methyl can be used in the control of adult Ae. aegypti in the Afar Region of Ethiopia. However, further studies should be carried out to evaluate the susceptibility of Ae. aegypti to alpha-cypermethrin insecticide in the Awash Arba area.
**Order of Authors:**	Mohammed Seid
	Esayas Aklilu
	Abebe Animut
**Response to Reviewers:**	Comments on journal requirements<br>1.Follow the PLOS ONE Journal submission guidelines.<br>Response: We have followed PLOS ONE Journal submission guidelines throughout the revised manuscript.<br>2.Funding information:<br>Response: we have provided the funding information. The statement “the study was financially supported by Addis Ababa University the Vice President for Research and Technology Transfer Office through its thematic research grant” has been included. Unfortunately, we did not include the funding statement in the submission system before. Thus, we kindly request you to incorporate the funding statement on the online submission system.<br>3.Non-author contact information:<br>Response: To ensure guarantee for the long-term stability and availability of the data,

Powered by Editorial Manager® and ProduXion Manager® from Aries Systems Corporation

we provided the data used as supporting information file 1, supporting information file 2 and supporting information file 3. Supporting information file 1 shows measurement used to estimate duration in minutes in which KDT50 and KDT95 Aedes aegypti knocked-down and the 95% confidence interval were analyzed (Table 1 in the manuscript). Supporting information file 2 presents mortality values of Ae. aegypti used to build the percentage mortality graphs (graphs 2-4 in the manuscript). Supporting information file 3 reveals representative GPS points of the study sites recorded during Ae. aegypti larvae and pupae collection. We mapped the study areas using Ethio-region GIS shape files to show the location of the study sites. We did not use map image for analysis in the result section of the manuscript.

4.Figure 1 copy-right:

Response: Authors have mapped the study area (named as Figure 1 in the manuscript) using arc GIS software version 10.8 from GIS Shape files of Ethiopia freely and no individual or organization involved. Thus, there is no copy-right issue for this figure.

Additional Editor’s comments

All reviewers in felt that the manuscript lacked sufficient detail to evaluate. In particular they requested more detail on the methodology including the sampling methods and statistical analyses performed. They have also listed additional specific changes that should be addressed prior to resubmission.

Response: We have provided details on each section as per the comments given by the reviewers. We have made slight modification where necessary in the revised manuscript.

Reviewer #1

Major comments

1.What criteria underlie the selection of insecticides in this study, specifically the inclusion of three pyrethroids and two carbamates? Additionally, are these insecticides currently in use for public health purposes?

Response: Viral diseases outbreaks are continuously reported in Ethiopia including the present study areas. Insecticide based viral diseases transmitting mosquito control is a quick responses during outbreaks. Thus, “the insecticides were selected for the study because they have been used for the control of mosquitoes of public health importance in Ethiopia and the study area”. “The insecticides are also among the insecticide classes used in the country”. The statements have been included in the methods (phenotypic susceptibility of Aedes aegypti) sub-section of revised manuscript. Yes, most are being used depending on the local transmission situation.

2.According to the WHO guideline cited in the methodology section, the recommended discriminating doses for susceptibility tests of Aedes mosquitoes using pyrethroids are 0.25% Permethrin, 0.03% alpha-cypermethrin, and 0.03% deltamethrin. However, it appears that different discriminating doses of pyrethroids were used for the study. Why it is different? Response: Thank you for the important question. The insecticides are used for public health in the study areas in particular and in Ethiopia in general with the same concentration of the present study tests. Thus, we used concentrations of the insecticides, WHO criteria, used to evaluate insecticide susceptibility status of Anopheles arabiensis. Furthermore, studies from Tanzania and Burkina Faso were used similar concentration of insecticides to test Aedes aegypti susceptibility to insecticides (Kahamba et al., 2020; Namountougou et al., 2020).

3.Why only phenotypic resistance studied? Was there a specific reason for not investigating the resistance mechanisms at play in the mosquito populations?

Responses: We share the comment as a limitation. We were not able to detect resistant genes to bendiocarb and propoxur due to lack of the reagents. We recommend further molecular investigations regarding the resistant genes.

4.How did you identified the Aedes mosquitoes used for the susceptibility tests were all Ae. Aegypti?

Responses: Experienced personnel were carried out the collection and insecticide susceptibility tests. Selected numbers of the mosquitoes were identified before the test as Ae. aegypti. All the mosquitoes were checked for being Ae. aegypti at the end of each experiment. All the mosquitoes were identified as Ae. aegypti.

5.How and why was the association between pyrethroid resistance in the study area and agricultural practices established?

Responses: Pyrethroids are mainly used in the control of mosquitoes that transmit viral diseases and malaria. They are also used in the control of agricultural pests (Shafer et al., 2008; Chrustek et al., 2018). It is known that insecticides used in agricultural practices induce public health insects to develop resistance against public health

insecticides. Because some insecticides used in agricultural insects share a similar mechanism of action and bio-active ingredients with public health insecticides. In the study areas, farmers used the pyrethroids to control pests of crops. As a result, uses of the insecticides in agriculture might potentially contribute to resistant mosquitoes.
Miner comments
1.Are Ae. aegypti and Ae. bromeliae the only two species these the only two Aedes species existing in the country? For example Ae. vittaus and Ae. africanus also exist in the country.
Responses: Thank you for your relevant comment. The statement “In Ethiopia, Ae. aegypti, Ae. bromeliae, Ae. vittatus, Ae. hirsutus, Ae. simpsoni complex and Ae. africanus are implicated as the potential vectors of viral diseases” has been included in the revised manuscript as suggested.
2.Why the Knockdown effect of permethrin and alpha-cypermethrin not elaborated like that of deltamethrin?
Responses: The statement “On the other, Ae. aegypti exposed to permethrin showed the lowest KDT50 in Awash Arba”, has been included in the revised manuscript as commented.
3.The vertical axis maximum value should not exceed 100 (Fig 2). This works for all graphs.
Responses: We have corrected the vertical axis of all the graphs to 100 in the revised manuscript as suggested.
4.The susceptibility test of alpha-cypermethrin in Awash Arba town repeated or not?
Response: Yes, the test was repeated. The statement “Ae. aegypti susceptibility test was repeated for alpha-cypermethrin in Awash Arba town since Ae. aegypti populations were appeared to be suspected phenotypic resistance to this insecticide” has been included in the revised manuscript.
5.Why is integrated vector control strategies recommended without supporting data from the study findings?
Responses: Thank you for the relevant question. We modified the conclusion based on the findings of the study in the revised manuscript.
6.How are pyrethroids currently utilized in the control of vectors?
Response: Pyrethroids are utilized currently to control mosquito vectors in the form of long lasting insecticides treated nets or in the insecticide residual spray. Moreover, recently the mosquito vector emerge resistance to pyrethroids. Thus, potential new approaches existing pyrethroids are emerging such as pyrethroids plus PBO (pyrethroid synergist piperonyl butoxide) which are used to break the pyrethroid resistance by blocking metabolic resistance. The PBO are not acting as insecticides but they facilitate the action of the pyrethroids.
Suggestions:
Response: All the suggestions given have been included in the revised manuscript.
Reviewer #2
I suggest improvement in the manuscript and offer the following comments and suggestions:
Overall: correct numerous typos and improve English language; ensure the references cited in the text match with those in the References list.
Response: The grammar and typos have been checked and corrected in the revised the manuscript. Similarly, all references cited in the text have been checked their correctness in the reference list.
Abstract:
- Background: In the last sentence of the “Background”, mention names of the insecticides instead of stating “public health insecticides”.
Response: “deltamethrin, permethrin, alpha-cypermethrin, pirimiphos-methyl, bendiocarb and propoxur” have been included in the revised manuscript in the last sentence of Abstract’s background.
-Methodology: Modify the sentence “Mortality was recorded after 24 hours” to “The mortality in mosquitoes was recorded 24 hours after 60 minutes of exposure”. Delete the abbreviation “KDT” for knockdown rate since later you have used KDT for the knockdown time.
Response: “The abbreviation “KDT” has been deleted. The mortality in mosquitoes was recorded 24 hours after 60 minutes of exposure” has been added in revised manuscript as suggested.
Results: In this sentence KDT95 implies Knockdown time, so modify the sentence to “Aedes aegypti from Awash Sebat showed high knock down time (KDT95 34.01 minutes) to deltamethrin ….”

Powered by Editorial Manager® and ProduXion Manager® from Aries Systems Corporation

Response: “Deltamethrin in Ae. aegypti showed high knockdown time (KDT95 34.01 minutes) in Awash Sebat as compared to other towns has been rephrased.
-Results: Rename “alphacypermethrin” to “alpha-cypermethrin”, “delthamethrin” to “deltamethrin” and “pirimiphos methyl” to “pirimiphos-methyl” throughout the manuscript (in text, figures, and tables).
Response: “alphacypermethrin” to “alpha cypermethrin”, “delthamethrin” to “deltamethrin”, “pirimiphosmethyl” to “pirimiphos-methyl” have been renamed in the entire revised manuscript sections as suggested.
- Conclusion: delete “control” at the end of the second sentence. Mention the insecticide discriminating concentration for each insecticide impregnated paper used. Also state that further studies are required to confirm resistance to alpha-cypermethrin.
Response: The word control has been deleted from the conclusion. In addition, the statement “However, further studies should be carried out to evaluate the susceptibility of Ae. aegypti to alpha-cypermethrin insecticide in the Awash Arba area”, has been included in the revised manuscript.
-Introduction
-Correct typos: change “Ae. albopictus[11, 12].” to “Ae. albopictus [11, 12].”, “Ae .aegypti” to “Ae. aegypti”, “Fenitrothion” to “fenitrothion”.
Response: “Ae. albopictus[11, 12],” has been corrected to “Ae. albopictus [11, 12].” Ae .aegypti” to “Ae. aegypti”, “Fenitrothion” to “fenitrothion”, in the revised the manuscript as suggested.
-Material and Methods
Study sites: Correct many typos (e.g. “820masl” to “820 masl” and so on.
Response: “820masl” to “820 masl”, “22.6oC to 30.6oC” to “22.6 oC to 30.6 oC” and “606.6mm” to “606.6 mm” have been corrected in the revised manuscript.
Exactly what insecticide spraying is practiced in the study areas for the control of malaria and arboviral diseases to give an indication of insecticidal interventions used.
Response: The statement “Insecticide such as deltamethrin, permethrin, alpha-cypermethrin, pirimiphos-methyl bendiocarb and propoxur are used,” has been included in the revised version of the manuscript.
Figure 1 is not mentioned in the text under the study sites.
Response: Fig 1, has been cited in the text in the revised manuscript.
Insecticide susceptibility:
- Reference # 16 attributed to the WHO procedure is incorrect. Please recheck correctness of all references used in the text.
Response: All the references have been checked and reference number 16 has been corrected by reference 20 in the revised manuscript.
- Correct numerous typos such as “delta-methrin” to “deltamethrin”.
Response: delta-methrin” has been corrected to “deltamethrin in the revised manuscript.
- A correct number of mosquitoes (100) were exposed in the treatments but not in the control (40 instead of 50). This is a slight deviation from the WHO tube test procedure.
Response: We followed the WHO 2016 insecticide susceptibility test and exposed the control mosquitoes following the procedures in that manual.
- “fed on 10% sugar solution” is an incorrect statement; instead, state “were offered cotton wool pads soaked in 10% sugar solution”.
Response: the statement “were offered cotton wool pads soaked in 10% sugar solution” has been corrected in the revised manuscript as suggested.
Data analysis
Correct this statement “suspected for resistance were with a mortality 90-97%” to “suspected of resistance were with a mortality of 90–97%”.
Response: the statement “suspected of resistance were with a mortality of 90–97%” has been added in the revised manuscript.
Reference # 34 is incorrect; the correct reference should be: WHO (2022). Manual for monitoring insecticide resistance in mosquito vectors and selecting appropriate interventions. https://iris.who.int/handle/10665/356964.
Response: Since we initially used WHO (2016). Monitoring and managing insecticide resistance in Aedes mosquito populations: interim guidance for entomologists. Thus reference # 34 has been corrected by reference 20.
-Correct the abbreviations “50% (KD50) and 95% (KD95)” to “50% (KDT50) and 95% (KDT95).
Response: The abbreviations “50% (KD50) and 95% (KD95)” have been corrected to “50% (KDT50) and 95% (KDT95) in the revised manuscript as suggested.
-Results

-In Table 1 header, change “KDT50±SE” to “KDT50 ± SE” and “KDT95±SE” to “KDT95 ± SE”
Response: We have used estimated “KDT50 “KDT95 with their 95% confidence interval in the revised manuscript.
- Correct numerous typos in the text and figures.
Response: we have edited typos in the text and corrected the vertical axis of the figures in the revised manuscript.
-Discussion:
-Is there any publication supporting the following statement “Ae. aegypti is generally considered as exophilic, exophagic …....”.
Response: reference number 37 cited in this study support the high exophilic and exophagic-ness of Ae. aegypti.
- While KDT95 for deltamethrin in one town was higher, the tube test does confirm full susceptibility of Ae. aegypti.
Response: The KDT95 for deltamethrin in Awash Sebat higher as compared to other pyrethroids in the study. Thus, it did not necessarily confirm resistance or suspect resistance since the mortality of Ae. aegypti were determined after 24 hrs.
- Some of the WHO insecticide discriminating concentrations for Aedes aegypti adults have changed in 2022 following the publication of the above said WHO manual based on a WHO multi-centre study in which the authors' institution was a collaborator, as follows:
o deltamethrin 0.05% to 0.03%
o permethrin 0.75% to 0.40%
o bendiocarb 0.1% to 0.2%
o pirimiphos-methyl 0.25% to 60 mg/m2
o alpha-cypermethrin 0.05% : no change
o propoxur 0.1% : no change
-Since Ae. aegypti was found resistant to bendiocarb 0.1% concentration while its concentration has been increased to 0.2%, discuss that further tests should be conducted with 0.2% impregnated papers to confirm resistance.
Response: the statements “World health organization in 2022 has recommended that the insecticide discriminating dose of bendiocarb for Ae. aegypti is changed from its initial 0.1% to 0.2% [45]. Thus, future efficacy studies of bendiocarb should target the 0.2% concentration”, have been added in the revised manuscript.
References
- I did not check correctness of each reference cited, but I noticed at least two incorrect references, therefore authors should recheck all references cited.
Response: All the references cited in the text have been checked to their correctness in the reference list.
Reviewer #3:
The authors have a good reason for conducting this research, the manuscript is well organized and written clearly enough to be accessible to non-specialists. However, lacks a detailed description of the method used to collect larvae and pupae.
It is understood that they collected in three places in the South of the Afar region of Ethiopia, where vector-transmitted infections occur, and that they did so from July to October 2022. However, they do not specify how many times each of the locations were visited during that 3 to 4 months for the collections to obtain a variety of samples, nor how many breeding sites they found from where they collected the specimens.
Response: The statements “A cross-sectional study was designed to collect larvae and pupae of Aedes species from July to October, 2022 in the Awash Sebat, Awash Arba and Werer towns. The study towns were selected purposively in collaboration with the local district health professionals and on the basis of the recent outbreak reports of dengue fever and chikungunya virus. The towns were surveyed once per month”, have been included in the revised manuscript.
The latter is important because it is not known if the offspring they collected come from the same progeny or from few of them, hence the importance of collecting in different breeding sites and at different collection times in the same place. The material collected on the same day at various breeding sites of a locality must be combined so that there is mixing of the genes and thus the results of resistance to insecticides may correspond to several parental mosquitoes originated from the collected site. It is important to describe the number of samples collected on the dates in each of the locations. If possible, describe the number of larvae and pupae collected or an approximate number.
Response: The objective of the study was to collect sufficient number of Ae. aegypti

larvae and pupae for the susceptibility test. We did not record habitat characteristics, counts of larvae and pupae.
By other hand, it is not specified how many experiments were carried out for the tests with each insecticide. It is understood that only 100 mosquitoes were used and 40 for two control tubes, so that makes only a test. However, at least two other replicates of collections separated in time must be carried out for the same collection location. In this way you can work with an average mortality for more precise data on the locality that is being collected, and if differences in mortalities are found in the three collections could be a good motive for discussion in this manuscript to enrich the discussion.
Response: Thank you for your relevant suggestion. The tests were performed in replicates (five treatment and two control replicates) for each insecticide as per the World Health Organization 2016 criteria. We believe that the results obtained are sufficiently enough to support the conclusion drawn in the study.
-Discussion, 3rd paragraph, at the end: referring to the phrase "the findings observed in the present study were lower than results reported in Sudan, Tanzania and Nigeria" it would be good to add if those from Sudan, Tanzania and Nigeria were reported as resistant or only tolerants to deltamethrin, and maybe is good to mention their KDT50 and 95 values, so we can have an idea for comparison.
Response: Comparison of KDT50 and KDT95 values of deltamethrin to the study carried out in Sudan, Nigeria and Tanzania has been made. The statement “Overall the tube test revealed that Ae. aegypti were susceptible to deltamethrin in Sudan, Tanzania but suspected resistance in Nigeria,” has been included at the end of discussion section 3rd paragraph in the revised manuscript.
A sentence could be added in this section about the resistance mechanisms that could be present in the mosquitoes detected resistant to propoxur and bendiocarb, but not to organophosphate. It is known that the two groups of insecticides share the same site of action, and that if resistance to both groups has been diagnosed, such a situation could explain a resistance based on a point mutation in the site of action, however this is not the case.
Response: The statement “In addition, Ae. aegpti resistance to propoxur and bendiocarb may occur as a result of mutation in the insecticide target site enzyme Acetyl-cholinesterase (AChE) as reported in Cuba,” has been included in the revised manuscript.
Minor corrections:
Introduction, 3rd paragraph. "Fenitrothion" change to "fenitrothion"
Response: “Fenitrothion" has been changed to "fenitrothion" in the revised manuscript.
Material and Methods: 1st paragraph. "Were" change to "Werer"
Response: "Were" has been corrected to "Werer" in the revised manuscript.
Insecticide susceptibility paragraph: "delta-methrin"change to "deltamethrin" and "bendio carb" change to bendiocarb"
Response: delta-methrin" has been changed to "deltamethrin" and "bendio carb" has been changed to bendiocarb" in the revised manuscript.
-Results, 1st paragraph, Table 1. "Ae. Aegypti" change to "Ae. aegypti"
Response:"Ae. Aegypti" has been changed to "Ae. aegypti" in the revised manuscript
Discussion, 4th paragraph. "propxur" change to "propoxur"
Response: propxur" has been change to "propoxur" in the revised manuscript
Conclusion. "perimethrin" change to "permethrin"
Response: "perimethrin" has been changed to "permethrin" in the revised manuscript.
Check the double spaces between words throughout the writing and correct them to one space.
Response: We have made the manuscript text double space while one space between words in the revised manuscript.
Reference

Kahamba NF, et al. Habitat characteristics and insecticide susceptibility of Aedes aegypti in the Ifakara area, south-eastern Tanzania. Parasit Vectors. 2020; 13(1) 1-15.
Namountougou M, et al. Monitoring Insecticide Susceptibility in Aedes Aegypti Populations from the Two Biggest Cities, Ouagadougou and Bobo-Dioulasso, in Burkina Faso: Implication of Metabolic Resistance. Trop. Med. Infect. Dis. 2020; 5(84):1-9.
Shafer TJ, Rijal SO, Gross GW. Complete inhibition of spontaneous activity in neuronal networks in vitro by deltamethrin and permethrin. Neuro Toxicology. 2008;29:20 212.
Chrustek A, Hołyńska-Iwan I, Dziembowska I, Bogusiewicz J, Wróblewski M, Cwynar A. et al. Current Research on the Safety of Pyrethroids Used as Insecticides. 2018; 54(4): 61

Additional Information:	
**Question**	**Response**
**Financial Disclosure**<br><br>Enter a financial disclosure statement that describes the sources of funding for the work included in this submission. Review the submission guidelines for detailed requirements. View published research articles from *PLOS ONE* for specific examples.<br><br>This statement is required for submission and **will appear in the published article** if the submission is accepted. Please make sure it is accurate.<br><br>**Funded studies**<br>Enter a statement with the following details:<br>• Initials of the authors who received each award<br>• Grant numbers awarded to each author<br>• The full name of each funder<br>• URL of each funder website<br>• Did the sponsors or funders play any role in the study design, data collection and analysis, decision to publish, or preparation of the manuscript?<br><br>Did you receive funding for this work?	Yes
Please add funding details.<br>as follow-up to "**Financial Disclosure**<br><br>Enter a financial disclosure statement that describes the sources of funding for the work included in this submission. Review the submission guidelines for detailed requirements. View published research articles from *PLOS ONE* for specific examples.<br><br>This statement is required for submission and **will appear in the published article** if the submission is accepted. Please make sure it is accurate.	The study was financially supported by Addis Ababa University the Vice President for Research and Technology Transfer Office through its thematic research grant.

**Funded studies**<br>Enter a statement with the following details:<br>• Initials of the authors who received each award<br>• Grant numbers awarded to each author<br>• The full name of each funder<br>• URL of each funder website<br>• Did the sponsors or funders play any role in the study design, data collection and analysis, decision to publish, or preparation of the manuscript?<br><br>Did you receive funding for this work?"	
Please select the country of your main research funder (please select carefully as in some cases this is used in fee calculation).<br>as follow-up to "**Financial Disclosure**<br><br>Enter a financial disclosure statement that describes the sources of funding for the work included in this submission. Review the submission guidelines for detailed requirements. View published research articles from *PLOS ONE* for specific examples.<br><br>This statement is required for submission and **will appear in the published article** if the submission is accepted. Please make sure it is accurate.<br><br>**Funded studies**<br>Enter a statement with the following details:<br>• Initials of the authors who received each award<br>• Grant numbers awarded to each author<br>• The full name of each funder<br>• URL of each funder website<br>• Did the sponsors or funders play any role in the study design, data collection and analysis, decision to publish, or preparation of the manuscript?<br><br>Did you receive funding for this work?"	
**Competing Interests**	The authors declared that there is no conflict of interest.

Use the instructions below to enter a competing interest statement for this submission. On behalf of all authors, disclose any competing interests that could be perceived to bias this work—acknowledging all financial support and any other relevant financial or non-financial competing interests.<br><br>This statement is required for submission and **will appear in the published article** if the submission is accepted. Please make sure it is accurate and that any funding sources listed in your Funding Information later in the submission form are also declared in your Financial Disclosure statement.<br><br>View published research articles from *PLOS ONE* for specific examples.<br><br>**NO authors have competing interests**<br><br>Enter: *The authors have declared that no competing interests exist.*<br><br>**Authors with competing interests**<br><br>Enter competing interest details beginning with this statement:<br><br>*I have read the journal's policy and the authors of this manuscript have the following competing interests: [insert competing interests here]*<br><br>* typeset	
**Ethics Statement**<br><br>Enter an ethics statement for this submission. This statement is required if the study involved:<br><br>• Human participants<br>• Human specimens or tissue<br>• Vertebrate animals or cephalopods<br>• Vertebrate embryos or tissues	"N/A"

Powered by Editorial Manager® and ProduXion Manager® from Aries Systems Corporation

• Field research<br><br>Write "N/A" if the submission does not require an ethics statement.<br><br>General guidance is provided below. Consult the submission guidelines for detailed instructions. **Make sure that all information entered here is included in the Methods section of the manuscript.**	

Powered by Editorial Manager® and ProduXion Manager® from Aries Systems Corporation

**Format for specific study types**<br><br>**Human Subject Research (involving human participants and/or tissue)**<br>• Give the name of the institutional review board or ethics committee that approved the study<br>• Include the approval number and/or a statement indicating approval of this research<br>• Indicate the form of consent obtained (written/oral) or the reason that consent was not obtained (e.g. the data were analyzed anonymously)<br><br>**Animal Research (involving vertebrate animals, embryos or tissues)**<br>• Provide the name of the Institutional Animal Care and Use Committee (IACUC) or other relevant ethics board that reviewed the study protocol, and indicate whether they approved this research or granted a formal waiver of ethical approval<br>• Include an approval number if one was obtained<br>• If the study involved *non-human primates*, add *additional details* about animal welfare and steps taken to ameliorate suffering<br>• If anesthesia, euthanasia, or any kind of animal sacrifice is part of the study, include briefly which substances and/or methods were applied<br><br>**Field Research**<br><br>Include the following details if this study involves the collection of plant, animal, or other materials from a natural setting:<br>• Field permit number<br>• Name of the institution or relevant body that granted permission	
**Data Availability**<br><br>Authors are required to make all data underlying the findings described fully available, without restriction, and from the time of publication. PLOS allows rare exceptions to address legal and ethical concerns. See the PLOS Data Policy and FAQ for detailed information.	Yes - all data are fully available without restriction

A Data Availability Statement describing where the data can be found is required at submission. Your answers to this question constitute the Data Availability Statement and **will be published in the article**, if accepted.<br><br>**Important:** Stating 'data available on request from the author' is not sufficient. If your data are only available upon request, select 'No' for the first question and explain your exceptional situation in the text box.<br><br>Do the authors confirm that all data underlying the findings described in their manuscript are fully available without restriction?	
**Describe where the data may be found in full sentences. If you are copying our sample text, replace any instances of XXX with the appropriate details.**<br><br>• If the data are **held or will be held in a public repository**, include URLs, accession numbers or DOIs. If this information will only be available after acceptance, indicate this by ticking the box below. For example: *All XXX files are available from the XXX database (accession number(s) XXX, XXX.*).<br>• If the data are all contained **within the manuscript and/or Supporting Information files**, enter the following: *All relevant data are within the manuscript and its Supporting Information files.*<br>• If neither of these applies but you are able to provide **details of access elsewhere**, with or without limitations, please do so. For example:<br><br>*Data cannot be shared publicly because of [XXX]. Data are available from the XXX Institutional Data Access / Ethics Committee (contact via XXX) for researchers who meet the criteria for access to confidential data.*<br><br>*The data underlying the results presented in the study are available from (include the name of the third party*	All relevant data are within the manuscript and its Supporting Information files.

and contact information or URL).<br>• This text is appropriate if the data are owned by a third party and authors do not have permission to share the data.<br><br>* typeset	
Additional data availability information:	

Powered by Editorial Manager® and ProduXion Manager® from Aries Systems Corporation

Manuscript Click here to access/download;Manuscript;Manuscript.doc

Susceptibility status of *Aedes aegypti* (Diptera: Culicidae) to public health insecticides in Southern Afar region, Ethiopia

Mohammed Seid[1, 2*], Esayas Aklilu[1], Abebe Animut[1]

[1] Aklilu Lemma Institute of Pathobiology, Addis Ababa University, Addis Ababa, Ethiopia

[2] Department of Biology, College of Natural and Computational Sciences, Mattu University, Mattu, Ethiopia

*Corresponding author

Email: mohammed.seid.legas@gmail.com (MS)

Abstract

Mosquito-borne viral diseases such as dengue fever, chikungunya and yellow fever have been documented in Ethiopia since the 1960s. However, efficacy of public health insecticides against *Aedes aegypti* that transmit these viruses remain poorly understood in the country particularly in the Afar region. Thus, the aim of this study was to assess the susceptibility status of *Ae. aegypti* to deltamethrin, permethrin, alpha-cypermethrin, pirimiph os-methyl, bendiocarb and propoxur insecticides. Larvae and pupae of *Aedes* mosquitoes were collected from the Awash Arba, Awash Sebat and Werer towns of the Afar Regional State of Ethiopia during July to October 2022, brought to Aklilu Lemma Institute of Pathobiology, insectary and reared to adults. Non-blood-fed, ~~Non-blood fed~~ 3 to 5 days old female *Ae. aegypti* were exposed to pyrethroid, carbamate ~~pyrethroids, carbamates~~ and organophosphate insecticides following the World Health Organization standard guidelines. Knockdown rates were noted at 10 min interval until one hour. The mortality in mosquitoes was recorded 24 hours after 60 minutes of exposure. Mortalities of *Ae. aegypti* exposed to propoxur were 87% in all the study towns. Similarly, 88% of *Ae. aegypti* mortality was recorded when treated with bendiocarb in Awash Sebat and Awash Arba towns. Suspected resistance of *Ae. aegypti* (95% mortality) exposed t o alpha-cypermethrin was ~~were~~ recorded from Awash Arba town. However, *Ae. aegypti* collected from all the three sites was were observed to be susceptible to deltamethrin, permethrin, and

pirimiphos-methyl. *Aedes aegypti* was resistant to 0.1% bendiocarb and 0.1% propoxur, and possibly resistant to 0.05% alpha-cypermethrin. On the other hand, it was susceptible to 0.05% deltamethrin, 0.75% permethrin and 0.25% pirimiphos-methyl. Thus, deltamethrin, permethrin and pirimiphos-methyl can be used in the control of adult *Ae. aegypti* in the Afar Region of Ethiopia. However, further studies should be carried out to evaluate the susceptibility of *Ae. aegypti* to alpha-cypermethrin insecticide in the Awash Arba area.

Introduction

The ever increasing outbreaks of arboviral diseases drew public attention in several countries of the world [1]. The East African countries including Kenya, Somalia, Djibouti, Eritrea and Ethiopia are epicentres of outbreaks of arboviral diseases such as dengue fever, yellow fever and chikungunya virus [2-4]. In Ethiopia, yellow fever caused about 30,000 deaths from over 200,000 infections during the 1960 and 1962 [5]. Recently, there have been yellow fever outbreaks in South Omo [6], dengue fever outbreaks in Dire Dawa city administration, Somali Regional State, Gewane and Amibara districts of Afar Regional State [7, 8]. In addition, chikungunya outbreaks were also reported in Dire Dawa city administration, Afar and Somali Regional States [9, 10]. All these outbreaks with potentially unrecognized and/or under reported deaths owing to the highly limited laboratory facilities in the country.

Mosquitoes transmit over 90% of the viral pathogens through their bites among which *Ae. aegypti* is the leading vector followed by *Ae. albopictus* [11, 12]. *Ae. aegypti* is anthropophilic in its behaviour and occurs in close proximity to human habitations [13, 14]. *Ae. aegypti* has two morphological subspecies (ecotypes) the domestic *Ae. aegypti aegypti* and sylvan *Ae. aegypti formosus* [15, 16]. In Ethiopia, *Ae. aegypti, Ae. bromeliae, Ae. vittatus, Ae. hirsutus, Ae. simpsoni* complex and *Ae. africanus* are implicated as the potential vectors of viral diseases [6, 8, 17-19].

In the absence of effective therapeutic drugs and vaccines to treat and control mosquito borne viral diseases, preventions largely depend on vector control tools mainly public health insecticides [13]. The pyrethroids such as deltamethrin, alpha-cypermethrin, permethrin and organophosphates primiphos-methyl and fenitrothion are among the public health insecticides used against *Aedes* mosquitoes [20]. However, the potential resistance to the insecticides may adversely affect implementation of the control strategy in endemic areas [21]. Mosquitoes could resist insecticides through their inherent ability to detoxify and avoid contacts either behaviourally or physiologically [22].

Evidences from many African countries indicate that the *Anopheles* and *Aedes* mosquitoes developed resistance to insecticides [23-25]. For instance in Ethiopia, malaria transmitting *Anopheles arabiensis* has developed resistance to insecticides in several areas of the country including in the Amibara district of the Afar Regional State [25, 26]. The causes for the resistance could be frequent use of the insecticide chemicals in indoor residual spraying and long-lasting insecticidal treated nets for the control of mosquito borne diseases in the area [21] or in argro-pesticides [27]. However, the susceptibility status of *Aedes* mosquitoes to insecticides currently used is lacking in the country.

Recently, the Ethiopian Public Health Institute prepared arboviral disease vectors, surveillance and control guideline in 2021 through chemical and environmental management methods. Vector control by chemical method such as fumigation, repellents and residual treatments were implemented in Dire Dawa city administration and Somalia Regional State during dengue fever and chikungunya outbreaks (EPHI, 2021 unpublished). Unfortunately, there is a scarcity of evidences on the efficacy of the insecticides for the control of virus transmitting *Aedes* species in the Southern Afar Region in the presence of increased reports of viral diseases incidence and prevalence. Therefore, assessing the effectiveness of the existing public health insecticides to control *Aedes* species is crucial to strengthen the

indoor residual?

arboviral diseases prevention and control programs in the country. This inspired us to generate baseline base line data in relation to the applicability of insecticide control tools in the prevention and control of virus transmitting *Aedes* mosquitoes in the area. Therefore, the aim of the study was to assess the susceptibility status of *Ae. aegypti* to public health insecticides in the Southern part of the Afar Regional State of Ethiopia.

Materials and Methods

Study sites

The study was carried out in Awash Arba, Werer and Awash Sebat towns, Gabi-Rasu Zone (Zone 3), of Afar Regional State, Ethiopia. The Awash Arba and Werer towns are located about 250 km to the North-east of Addis Ababa at 9.33453^{o}N latitude, 40.181385^{o}E longitude and an altitude range of 720 metres above sea level (masl) to 1100 masl (Central statistics Agency, 2019 unpublished). Awash Arba and Werer sites were selected from Amibara district which are about 30 km distance far apart (Fig 1). Agro-ecologically, these two towns are semi-arid with temperature ranging from 25 °C to 35 °C, and average annual rainfall of 530 mm. The communities livelihoods are characterized as pastoralist and agro-pastoralist in which they mainly obtained income on livestock and from livestock and other agricultural crop production [28].

Awash Sebat town administration is located 214 km North-east of Addis Ababa at 8.98810^{o}N latitude and 40.163936^{o}E longitude (Central statistics Agency, 2019 unpublished). Its altitude ranges from 820 masl to 1120 masl and the temperature varies from 22.6 ^{o}C to 30.6 ^{o}C. The town and its surroundings receive a mean annual rainfall of 606.6 mm.

The study areas are found in the middle Awash Valley, where infectious disease such as malaria, chikungunya and dengue fever are common [29]. Considerable attention has been given by the regional government to achieve better health in the region including the three towns [30]. The region has hospitals, health centres, clinics and health posts in different

zones and districts. There are one hospital, one clinic and five health centres and few private clinics. Insecticide such as deltamethrin, permethrin, alpha-cypermethrin, pirimiphos-methyl bendiocarb and propoxur are used as insecticide residual spraying and/or long lasting insecticide treated nets for the control of malaria and arboviral diseases in these towns [26, 31]. Similarly, agro-chemicals and agro-pesticides are used since maize and sorghum productions are common in these areas which is used to improve agricultural crop production [32].

Fig 1. Map of the study areas. A map showing the locations of the three study areas**:** Awash Sebat, Awash Arba and Werer Towns, in the Southern Afar Region, Ethiopia.

Larval collection and rearing

A cross-sectional study was designed to collect larvae and pupae of *Aedes* species from July to October, 2022 in the Awash Sebat, Awash Arba and Werer towns. The study towns were selected purposively in collaboration with the local district health professionals and on the basis of the recent outbreak reports of dengue fever and chikungunya virus (Awash Sebat town and Amibara district health bureaus, 2022 unpublished). The towns were surveyed once per month. Larvae and pupae were collected from different positive habitats including tyres, water storage drums, discarded plastics and metallic containers using standard dipper (350 ml), ladles and pipets/nets. The collected larvae and pupae, from each habitat, were transferred with labelled plastic jars, transported to the insectary of Aklilu Lemma Institute of Pathobiology, Addis Ababa University, Ethiopia and reared to adults under standard laboratory conditions. Tetra cichlid floating fish food (®/TM/©2019 Germany) was provided as food to the larvae. The emergence of larvae in to pupae was checked each morning and pupae were collected in glass flasks and transferred in to (30cm x 30cm x 30cm) netted cages. Emerged adult *Ae. aegypti* were kept at 27 ± 2 °C temperature, 75 ± 10% relative

humidity and fed on 10% sucrose solution until the bioassay tests. Emergent adults were identified to species using morphological taxonomic keys for *Aedes* species [33].

Phenotypic susceptibility status of *Aedes aegypti*

Prior to insecticide susceptibility tests, sugar fed female *Ae. aegypti* were starved for 2hrs in order to prevent them from having high weight which leads them to be knocked-down. Non-blood fed, 3-5 days old female *Ae. aegypti* were tested for their susceptibility to insecticides following the World Health Organization insecticide testing procedures [20]. They were exposed to pyrethroids (0.05% deltamethrin, 0.05% alpha-cypermethrin, 0.75% permethrin), carbamates (0.1% bendiocarb and 0.1% propoxur) and organophosphates (0.25% pirimiphos -methyl) which are commonly used for preventing disease transmitting mosquitoes [20]. The insecticides were selected for the study because they have been used for the control of public health important mosquitoes in Ethiopia and the study area. The insecticides are also among the insecticide classes used in the country. Similar concentrations of insecticides were used to test the susceptibility of *Ae. aegypti* in Tanzania [34]. In each test, 20 female *Ae. aegypti* were exposed to each five treatment replicates and two control test tubes making a total of 140 mosquitoes in a single test. The exposed mosquitoes were observed for 60 minutes at an interval of 10 minutes to observe their knockdown. After an hour, the exposed females were transferred into holding tubes and kept for 24 h at 27.0 ± 2.0 °C and 75 ± 10% relative humidity and were offered cotton wool pads soaked in 10% sugar solution. The mortality in mosquitoes was recorded 24 hours after 60 minutes of exposure. *Ae. aegypti* susceptibility test was repeated for alpha-cypermethrin in Awash Arba town since *Ae. aegypti* populations were appeared to be suspect phenotypic resistance to this insecticide. However, in case of Werer town the susceptibility status of *Ae. aegypti* to bendiocarb was not conducted due to insufficient mosquito samples.

Ethical Issues

The study protocol obtained ethical approval from the institutional review board (IRB) of Aklilu Lemma Institute of Pathobiology, Addis Ababa University, Ethiopia (June 13, 2022; ALIPB IRB/80/2022). Written permission letters were obtained from the district health bureau and health centres of the towns. The households were informed about the objective of the study and their agreements were sought prior to the larvae/pupae collection.

Data analysis

Mortality was determined on the basis of the dead and alive mosquitoes after the 24 hours recovery period. Susceptible were those with a mortality in the range of 98-100%, suspected for resistance were with a mortality 90-97% and resistant if mortality was <90% [20]. The ~~knockdown effect of pyrethroids against the *Ae. aegypti* were recorded during the 60 minutes exposure at an interval of 10 minutes from the start.~~ Probit analysis was used to determine estimated duration at which 50% (KDT_{50}) and 95% (KDT_{95}) of the exposed *Ae. aegypti* mosquitoes were knocked-down. Overall adult mortalities and knockdown times were computed using SPSS Software version 20.

Results

Knockdown effect of insecticides against *Aedes aegypti*

Knockdown time of *Ae. aegypti* exposed to insecticides was depicted in Table 1**.** The knockdown effect of 50% (KDT_{50}) of *Ae. aegypti* exposed to deltamethrin ranged from 12.15 to 17.06 minutes whereas KDT_{95} of the insecticide ranged from 18.74 to 34.01 minutes. Deltamethrin in *Ae. aegypti* showed high knockdown time (KDT_{95}, 34.01 minutes) in Awash Sebat as compared to other towns (Table 1). On the other hand, *Ae. aegypti* exposed to permethrin showed the lowest KDT_{50} in Awash Arba town (KDT_{50}, 5.11). Overall, the KDT_{50} and KDT_{95} of *Ae. aegypti* varied among *Ae. aegypti* in knockdown to insecticides across the study localities.

Table 1 Knockdown times (in minute) of 50% and 95% of *Ae. aegypti* exposed to commonly used insecticides in three localities from July to October 2022 in Southern Afar region Ethiopia

Insecticides	Localities	KDT_{50} ~~95% [CI]~~ [95% CI]	KDT_{95} 95% [CI] [95% CI]
Deltamethrin	Awash sebat	17.06 [15.81-18.28]	34.01 [31.02-38.12]
	Awash Arba	12.15 [11.41-12.97]	18.74 [16.98-21.56]
	Werer	16.53 [15.37-17.67]	30.92 [28.24-34.62]
Permethrin	Awash Sebat	8.92 [7.34-10.26]	24.21 [21.23-28.93]
	Awash Arba	5.11 [2.91-6.94]	19.23 [16.23-24.09]
	Werer	15.12 [12.45-17.66]	29.89 [24.64-41.53]
Alpha-cypermethrin	Awash Sebat	14.35 [13.35-15.35]	24.93 [22.77-27.97]
	Awash Arba	13.47 [12.64-14.36]	20.78 [19.04-23.32]
	Werer	16.71 [13.85-19.46]	32.90 [27.26-44.98]

CI, Confidence interval; KDT_{50}, time taken for 50% of the tested Ae. *aegypti* to be knocked-down; KDT_{95} time taken for 95% of the tested *Ae. aegypti* to be knocked-down

Susceptibility status of *Aedes aegypti* to insecticides

The percentage mortalities of *Ae. aegypti* mosquito population against insecticides was presented in figures Fig. (figs 2-4). Accordingly, *Ae. aegypti* mosquitoes showed phenotypic resistance to bendiocarb and propoxur insecticides in which the percentage mortalities were recorded to be 88% and 87% respectively in Awash Sebat town. *Ae. aegypti* mortalities to deltamethrin, permethrin alph-acypermthrin and pirimiphos-methyl were 100% in Awash Sebat town (Fig 2). All mosquitoes in the control group showed 0% mortality in all study sites.

Fig 2. Insecticide susceptibility status of *Ae. aegypti* populations from Awash Sebat town, Southern Afar region, Ethiopia, July to October 2022. Error bars represent 95% confidence intervals.

resistant

In addition, *Ae. aegypti* mosquitoes were also observed resistance to bendiocarb and propoxur from Awash Arba. Moreover, *Ae. aegypti* mosquitoes were observed to be suspected resistant to alpha-cypermethrin in which the mortality of *Ae. aegypti* mosquitoes due to alpha-cypermethrin was 95% (Fig 3). *Ae. eagypti* mortalities were observed to be 100% when exposed to deltamethrin, permethrin and pirimiphos-methyl insecticides in this town.

Fig 3. Insecticide susceptibility status of *Ae. aegypti* populations from Awash Arba town, Southern Afar region, Ethiopia, July to October 2022. Error bars represent 95% confidence intervals.

Ae. aegypti mortalities when exposed to propoxur insecticide was 87% whereas 99% mortalities were observed among the *Ae. aegypti* mosquitoes from Werer town when treated with alpha-cypermethrin insecticide (fig 4). However, 100% mortalities of *Ae. aegypti* from this site were recorded to deltamethrin, permethrin and pirimiphos-methyl.

Fig 4. Insecticide susceptibility status of *Ae. aegypti* populations in Werer town, Southern Afar region, Ethiopia, July to October 2022. Error bars represent 95% confidence intervals.

Discussion

Recent outbreaks of dengue fever and chikungunya virus fever in the Eastern and the North-eastern parts of Ethiopia especially in Dire Dawa city administration, Somali Regional State and Amibara and Gewane districts of Afar regional State requires the need for the control of the *Aedes* species that transmit the viruses. The insecticide based mosquito control is underway in Ethiopia in general and in Afar regional State in particular [7, 35]. In so doing,

there have been continuous evaluation of efficacy of the insecticides against malaria transmitting *Anopheles* species but there is scarcity of research findings conducted to control *Aedes* species either chemically, biologically or other means of vector control strategies in the country [36]. Thus, the control of viral disease transmitting *Aedes aegypti* vector is poorly investigated and needs detailed research. As far as our knowledge is concerned, this study was first in its kind to report the susceptibility status of *Ae. aegypti* to public health insecticides in the Southern Afar Regional State and there are limited data to compare our findings to others in the country.

Aedes aegypti is generally considered as exophillic, exophagic and day time biters. They usually feed on human and other vertebrate hosts as described in previous studies [37, 38]. However, they may tend to be endophilic and endophagic in which they rest and fed indoors [39]. This makes controlling of the *Ae. aegypti* and the viral diseases they transmit more difficult [40]. As a result, different intervention tools have been adopted to control these mosquitoes including indoor residual insecticide spraying and long lasting insecticide treated nets in addition to managing their breeding habitats [41].

In the present study, *Ae. aegypti* showed higher KDT_{50} and KDT_{95} as compared to the study conducted in Sudan to deltamethrin insecticide. For instance, the KDT_{50} and KDT_{95} recorded for deltamethrin in Sudan were 13 and 19 minutes respectively which were lower than the current study in Awash Sebat and Werer towns [42]. However, study conducted in Nigeria revealed that the KDT_{50} of deltamethrin was almost two times higher than the KDT_{50} results observed in Awash Sebat [43]. Relatively similar permethrin KDT_{50} results were observed in Awash Sebat town and Mlabani, Tanzania. However, higher deltamethrin and permethrin KDT_{95} results were observed in Mlabani than all the present study towns [34]. Overall, the tube test revealed that *Ae. aegypti* were susceptible to deltamethrin in Sudan and Tanzania but suspected resistance in Nigeria.

Aedes aegypti varied in response to public health insecticides. *Ae. aegypti* populations were observed to be phenotypic resistant to bendiocarb where the mortality rates were less than 90%. *Ae. aegypti* resistance to bendiocarb was also reported in a previous study in Tanzania during the rainy season [34]. However, these finding were in contrary to the finding reported from Sudan, Nigeria and Cambodia [42-44]. World ~~health organization in~~ Health Organization 2022 has recommended that the insecticide discriminating dose of bendiocarb for *Ae. aegypti* is changed from its initial 0.1% to 0.2% [45]. Thus, future efficacy studies of bendiocarb should target the 0.2% concentration. [add reference here]

In the present study, *Ae. aegypti* ~~mortally~~ mortality against propoxur insecticide was assessed. It was observed that *Ae. aegypti* mortality when exposed to propoxur showed insecticide resistance in all the study towns. Similar findings were reported in Thailand where the efficacy of *Ae. aegypti* mortality to propoxur was 83% which indicated the phenotypic resistance of *Ae. aegypti* to propoxur in this area [46].

The possible contributing factors to the emergences of phenotypic resistance of *Ae. aegypti* in the present study areas could be frequent uses of insecticide chemicals such as insecticide treated bed net and indoor residual insecticide spraying to control mosquito borne diseases as previously indicated [47]. On the other hand, Ethiopia has long history in utilizing pesticides to increase agricultural production and to improve human health since 1964 [48, 49]. Unfortunately however, agro-pesticides induce insecticides resistance selection pressure in different ways. For instance, insecticides used to control mosquitoes can counteract to agro -pesticides used which have the same mode of action and bio-active ingredients might drive a selection pressure to chemical based insecticide control tools [27]. Similarly, agro-pesticide used in agriculture may ease insecticide tolerance as the pesticides washed to mosquito breeding habitats which confer resistance mechanism at the adult stages [50]. Thus, the emergence of insecticide resistance in bendiocarb and propoxur insecticides in the present

study could potentially arise from the frequent exposer of the mosquito to carbamate insecticides in agriculture used as an agro-pesticides or herbicides. In addition, *Ae. aegpti* resistance to propoxur and bendiocarb may occur as a result of mutation in the insecticide target site enzyme Acetyl-cholinesterase (AChE) as reported in Cuba [51].

The pyerethroids such as deltamethrin are chemical insecticides commonly used to control malaria and other mosquito borne diseases in Ethiopia. In the present study, the pyrethroid alpha-cypermethrin showed less than 97% of mortality of adult *Ae. aegypti* populations in the Awash Arba town which revealed the emergence of possible resistance in *Ae. aegypti* in this area. The emergence of possible resistance of *Ae. aegypti* mosquitoes in Awash Arba may be the frequent use of pyrethroids in the insecticide treated nets to control malaria in this area and agro-pesticides as discussed above. This may lead failer to vector control interventions programs in the study area and elsewhere in the country. Similar findings were reported in Ghana [23].

Complete susceptibility of *Ae. aegypti* populations were recorded to the organophosphate chemical insecticide i.e. pirimiphos-methyl in all the study areas. Likewise, the study conducted in Cambodia [44] reported similar findings in that the adult *Ae. aegypti* populations were completely susceptible for the pirimiphos-methyl even in its lower concentration than in the present study. However, this finding was in contrary to the findings reported in Nigeria [43]. Deltamethrin and permethrin of the pyrethroid insecticides are also showed 100% mortality to adult *Ae. aegypti* mosquito populations in the study areas. These findings were in contrary to the findings reported in Malaysia [52] where resistance phenotyp e were observed in permethrin and deltamethrin. However, similar findings was reported in Tanzania [34] and in Peru [53] where deltamethrin showed greater than 99% of mortality in adult *Ae. aegypti*. Thus, this findings could serve as a base line to the Ethiopian ministry of health and the Afar region health bureau to implement the chemical interventions particularly

the pyrethroids deltamethrin, permethrin and the organophosphate pirimiphos-methyl either in the form of aerial spray or surface treatment to reduce the burden of arboviral diseases during diseases outbreak in different parts of the country.

Conclusion

The present study reported that *Ae. aegypti* populations showed insecticide resistance to bendiocarb and propoxur and possible resistance to alpha-cypermethrin and complete susceptibility to deltamethrin, permethrin and pirimiphos-methyl insecticides. Thus, based on the findings, the following points would be forwarded. Continuous surveillances of *Aedes* species susceptibility to insecticides should be carried out in the study areas and elsewhere in Ethiopia including molecular aspects to mark and evaluate resistance pattern. Moreover, proper uses of existing insecticides are mandatory to hinder *Ae. aegypti* resistance to insecticides in the study areas. In addition, the organophosphate insecticide pirimiphos-methyl can be used in addition to deltamethrin and permethrin in the control of adult *Ae. aegypti* mosquitoes in Ethiopia.

Acknowledgement

The authors would like to acknowledge Aklilu Lemma Institute of Pathobiology Addis Ababa University, Ethiopia for providing us field and laboratory materials and insecticide impregnated papers. We also like to gratitude the vector biology and control units staffs, Mr Wosen Sisay for his technical assistance in the insectary. Finally, we appreciated all the communities and data collectors from Awash Sebat, Awash Arba and Werer towns for their collaboration during the study period.

Author contributions

Conceptualization: Mohammed Seid, Esayas Aklilu and Abebe Animut

Formal Analysis: Mohammed Seid

Investigations: Mohammed Seid, Esayas Aklilu and Abebe Animut

Methodology: Mohammed Seid, Esayas Aklilu and Abebe Animut

Supper-vision: Mohammed Seid, Esayas Aklilu and Abebe Animut

Validation: Mohammed Seid, Esayas Aklilu and Abebe Animut

Visualization: Mohammed Seid, Esayas Aklilu and Abebe Animut

Writing original draft: Mohammed Seid

Writing, reviewing and editing: Mohammed Seid, Esayas Aklilu and Abebe Animut

References

1. Braack L, et al. Mosquito-borne arboviruses of African origin: review of key viruses and vectors. Parasit vectors. 2018; 11(1):1-26. doi: 10.1186/s13071-017-2559-9 PMID: 29316963
2. Langat SK, et al. Origin and evolution of dengue virus type 2 causing outbreaks in Kenya: Evidence of circulation of two cosmopolitan genotype lineages. Virus Evolution. 2020; 6(1):1-9. doi: 10.1093/ve/veaa026 PMID: 32523778
3. Gutu MA, et al. Another dengue fever outbreak in Eastern Ethiopia-An emerging public health threat. PLoS Negl Trop Dis. 2021; 15(1):1-16. doi:10.1371/journal.pntd.0008992 PMID: 33465086
4. Usman A, et al. Dengue fever outbreaks in Eritrea, 2005-2015. Glob health research policy. 2016; 1(1):1-8. doi: 10.1186/s41256-016-0016-5 PMID: 29202065
5. Serie C, et al. Studies on yellow fever in Ethiopia. Epidemiologic study. Bulletin of the World Health Organization. 1968; 38(6):879-884. PMID: 53036636
6. Lilay A, et al. Re-emergence of yellow fever in Ethiopia after 50 years, 2013: epidemiological and entomological investigations. BMC Infect Dis. 2017; 17(1):1-6. doi: 10.1186/s12879-017-2435-4 PMID: 28506254
7. Woyessa AB, et al. The first acute febrile illness investigation associated with dengue fever in Ethiopia, 2013: a descriptive analysis. The Ethio J Health Develop. 2014; 28(3):155-161.

8. Mekuriaw W, et al. Epidemiological, entomological and climatological investigation of the 2019 dengue fever outbreak in Gewane district, Afar Region, North-east Ethiopia. Insects. 2022; 13(11):1-13. doi: 10.3390/insects13111066 PMID: 36421969

9. Geleta D, et al. Epidemiological description of chikungunya virus Outbreak in Dire Dawa Administrative City, Western Ethiopia, 2019. Int J Clin Exp Med Sci. 2020; 6(3): 41-45. doi: 10.11648/j.ijcems.20200603.13

10. Alayu M, et al. Risk factors for Chikungunya outbreak in Kebridhar City, Somali Ethiopia, 2019. Unmatched case-control study. bioRxiv. 2020:1-20.

11. Gubler DJ. Dengue, urbanization and globalization: the unholy trinity of the 21st century. Trop Med and health. 2011; 39(4SUPPLEMENT):1-12.
doi: 10.2149/tmh.2011-S05 PMID: 22500131.12

12. Li Y, et al. Urbanization increases *Aedes albopictus* larval habitats and accelerates mosquito development and survivorship. PLoS Negl Trop Dis. 2014; 8(11): 1-12. doi: 10.1371/journal.pntd.0003301 PMID: 25393814

13. WHO. Dengue: guidelines for diagnosis, treatment, prevention and control. World Health Organization. 2009;1-148. PMID: 2376296314

14. Powell JR. Mosquito-borne human viral diseases: why *Aedes aegypti*? The Am J Tro Med and Hyg. 2018; 98(6):1563-1565.doi: 10.4269/ajtmh.17-0866
PMID: 29557341

15. Mattingly P. Taxonomy of *Aedes aegypti* and related species. Bulletin of the World Health Organization. 1967;36(4):552-554. PMID: 43835441616.

16. Rose NH, et al. Climate and urbanization drive mosquito preference for humans. Current Biol. 2020; 30(18): 1-29. doi: 10.1016/j.cub.2020.06.092 PMID: 32707056

17. Getachew D, et al. Breeding sites of *Aedes aegypti*: potential dengue vectors in Dire Dawa, East Ethiopia. Interd Perspectives Infect Dis. 2015; 2015:1-8. doi: 10.1155/2015/706276 PMID: 2643571218

18. Ferede G, et al. Distribution and larval breeding habitats of *Aedes* mosquito species in residential areas of northwest Ethiopia. Epidemiol and health. 2018; 40: 1-7. doi: 10.4178/epih.e2018015 PMID: 29748457

19. Woyessa A, et al. *Aedes* Mosquitoes Distribution and Risk of Yellow fever transmission in Gurage Zone South-west Ethiopia. Ethio J Publ Health and Nutrition. 4(1):1-10.

20. WHO. Monitoring and managing insecticide resistance in *Aedes* mosquito populations: interim guidance for entomologists. 2016;1-11.21.

21. Moyes CL, et al. Contemporary status of insecticide resistance in the major *Aedes* vectors of arboviruses infecting humans. PLoS Negl Trop Dis. 2017; 11(7): 1-20. doi: 10.1371/journal.pntd.0005625 PMID: 28727779

22. WHO. Global plan for insecticide resistance management in malaria vectors 2012;13-132.

23. Owusu-Asenso CM, et al. Spatiotemporal distribution and insecticide resistance status of *Aedes aegypti* in Ghana. Parasit Vectors. 2022; 15(1):1-14. doi: 10.1186/s13071-022-05179-w PMID: 35183249

24. Mathias L, et al. Habitat productivity and pyrethroid susceptibility status of *Aedes aegypti* mosquitoes in Dar es Salaam, Tanzania. Infect Dis Poverty. 2017; 6(1):1-10. doi: 10.1186/s40249-017-0316-0 PMID: 2859565325.

25. Kenea O, et al. Impact of combining indoor residual spraying and long-lasting insecticidal nets on *Anopheles arabiensis* in Ethiopia: results from a cluster

randomized controlled trial. Malaria J. 2019; 18(1):1-11. doi: 10.1186/s12936-019-2811-1 PMID: 31126286

26. Messenger LA, et al. Insecticide resistance in *Anopheles arabiensis* from Ethiopia (2012–2016): a nationwide study for insecticide resistance monitoring. Malaria J. 2017; 16(1):1-14. doi: 10.1186/s12936-017-2115-2 PMID: 2915102427.

27. Nkya TE, et al. Impact of environment on mosquito response to pyrethroid insecticides: facts, evidences and prospects. Insect biochemistry and molecular biol. 2013; 43(4):407-416. DOI: 10.1016/j.ibmb.2012.10.006 PMID: 23123179

28. Mekuyie M, Jordaan A and Melka Y. Understanding resilience of pastoralists to climate change and variability in the Southern Afar Region, Ethiopia. Climate Risk Managt. 2018; 20: 64-77.

29. Gebru H, Ahmed J. Water quality assessments as implication to human health: a case study in awash sebat and its surrounding areas, Afar, Ethiopia. Inter J Earth Sci and Engi. 2016; 9(5):1999-2008.

30. Goshu D, et al. Socioeconomic Development in Afar Region: Achievements, Gaps and Priorities. 2021.

31. Ministry of Health Ethiopia. Vector control operational manual for malaria elimination in Ethiopia. 2017; 1-56.

32. Sileshi G, Zemedkun A and Nurhussien S. Assessment of the pesticide use, practice and hazards on cotton growing farmers in the Awash Valley Ethiopia. AgriRxiv. 2021(2021): 1-22. doi: https://cabidigitallibrary.org

33. Rueda LM. Pictorial keys for the identification of mosquitoes (Diptera: Culicidae) associated with dengue virus transmission. Walter Reed Army Inst of Research Washington Dc Department of Entomology. 2004;1-60.doi: 10.11646/zootaxa.589.1.1

34. Kahamba NF, et al. Habitat characteristics and insecticide susceptibility of *Aedes aegypti* in the Ifakara area, south-eastern Tanzania. Parasit Vectors. 2020; 13(1) 1-15. doi: 10.1186/s13071-020-3920-y PMID: 32033619

35. Ahmed YM, Salah AA. Epidemiology of dengue fever in Ethiopian Somali region: retrospective health facility based study. Cent Afr J Public Health. 2016; 2(2):51-6.

36. Waldetensai A, et al. *Aedes* mosquito responses to control interventions against the Chikungunya outbreak of Dire Dawa, Eastern Ethiopia. Inter JTrop Insect Sci. 2021; 41:2511-2520. doi: 10.1007/s42690-021-00430-w

37. Diouf B, et al. Resting behavior of blood-fed females and host feeding preferences of *Aedes aegypti* (Diptera: Culicidae) morphological forms in Senegal. J Med Entomol. 2021; 58(6):2467-2473. doi: 10.1093/jme/tjab111 PMID: 34165556

38. Ponlawat A, Harrington LC. Blood feeding patterns of *Aedes aegypti* and *Aedes albopictus* in Thailand. J Med Entomol. 2005; 42(5):844-849. doi: 10.1093/jmedent/42.5.844 PMID: 16363170

39. Diallo D, Diallo M. Resting behavior of *Aedes aegypti* in southeastern Senegal. Parasit Vectors. 2020; 13(1):1-7. doi: 10.1186/s13071-020-04223-x PMID: 32682436

40. Bartlow AW, et al. Forecasting zoonotic infectious disease response to climate change: mosquito vectors and a changing environment. Veter sci .2019; 6(2): 1.20. doi: 10.3390/vetsci6020040 PMID: 31064099

41. Lima EP, MOF Goulart and Rolim Neto ML. Meta-analysis of studies on chemical, physical and biological agents in the control of *Aedes aegypti*. BMC public health. 2015; 15:1-14. doi:10.1186/s12889-015-2199-y PMID: 26341708

42. Husham A, Abdalmagid M and Brair M. Status susceptibility of dengue vector; *Aedes aegypti* to different groups of insecticides in Port Sudan City-Red Sea State. Sudanese J Public Health. 2010; 5(4):199-202.

43. Ukpai OM, Ekedo CM. Insecticide susceptibility status of *Aedes aegypti* in umudike, Ikwuano lga Abia State, Nigeria. Animal Research Inter. 2018; 15(3):3082-3089.

44. Boyer S, et al. Monitoring insecticide resistance of adult and larval Aedes aegypti (Diptera: Culicidae) in Phnom Penh, Cambodia. Parasit Vectors. 2022; 15(1): 1-7. doi: 10.1186/s13071-022-05156-3 PMID: 35101104

45. WHO. Manual for monitoring insecticide resistance in mosquito vectors and selecting appropriate interventions. World Health Organization. 2022; 1-55.

46. Pethuan S, et al. Biochemical studies of insecticide resistance in *Aedes* (Stegomyia) *aegypti* and *Aedes* (Stegomyia) *albopictus* (Diptera: Culicidae) in Thailand. Trop Biomed. 2007; 24(1):7-15. PMID: 17568372

47. Barbosa S, et al. Modelling the impact of insecticide-based control interventions on the evolution of insecticide resistance and disease transmission. Parasit vectors. 2018; 11:1-21. doi: 10.1186/s13071-018-3025-z PMID: 30153869

48. Mengistie B. Policy-practice nexus: pesticide registration, distribution and use in Ethiopia. SM J Environ Toxicol. 2016; 2(1):1006-1019.

49. Negatu B, Dugassa S, Mekonnen Y. Environmental and health risks of pesticide use in Ethiopia. J Health Pollution. 2021; 11(30):1-12. doi: 10.5696/2156-9614-11.30.210601 PMID: 34267988

50. Poupardin R, et al. Cross-induction of detoxification genes by environmental xenobiotics and insecticides in the mosquito Aedes aegypti: impact on larval tolerance to chemical insecticides. Insect biochemistry molecular biolo. 2008; 38(5): 540-551. doi: 10.1016/j.ibmb.2008.01.004 PMID: 18405832

51. Bisset J, Rodríguez MM, Fernández D. Selection of insensitive acetylcholinesterase as a resistance mechanism in *Aedes aegypti* (Diptera: Culicidae) from Santiago de Cuba. J Med Entomol. 2006; 43(6):1185-1189. PMID: 17162951

52. Ishak IH, et al. Contrasting patterns of insecticide resistance and knockdown resistance (kdr) in the dengue vectors *Aedes aegypti* and *Aedes albopictus* from Malaysia. Parasit vectors. 2015; 8(1): 1-13. doi:10.1186/s13071-015-0797-2 PMID: 25888775

53. Pinto J, et al. Susceptibility to insecticides and resistance mechanisms in three populations of *Aedes aegypti* from Peru. Parasit vectors. 2019; 12(1):1-11. doi: 10.1186/s13071-019-3739-6 PMID: 31640810

Supporting information

S1 Table. Observed knockdown times (in minutes) and number of knocked-down female *Ae. aegypti* exposed to insecticides during the 60 minutes in Awash Sebat, Awash Arba and Werer towns of Southern Afar, July to October 2022.

S2 Table. Female *Ae. aegypti* mortalities exposed to insecticides observed after 24 hrs of recovery period from Awash Sebat, Awash Arba and Werer towns, Southern Afar Regional State, Ethiopia, July to October, 2022.

S3 Table. Representative GPS points of the Awash Sebat, Awash Arba and Werer towns of Southern Afar Regional State, Ethiopia recorded during *Ae. aegypti* larvae and pupae collection.

Figure

Click here to access/download;Figure;Fig 1. tiff.tif

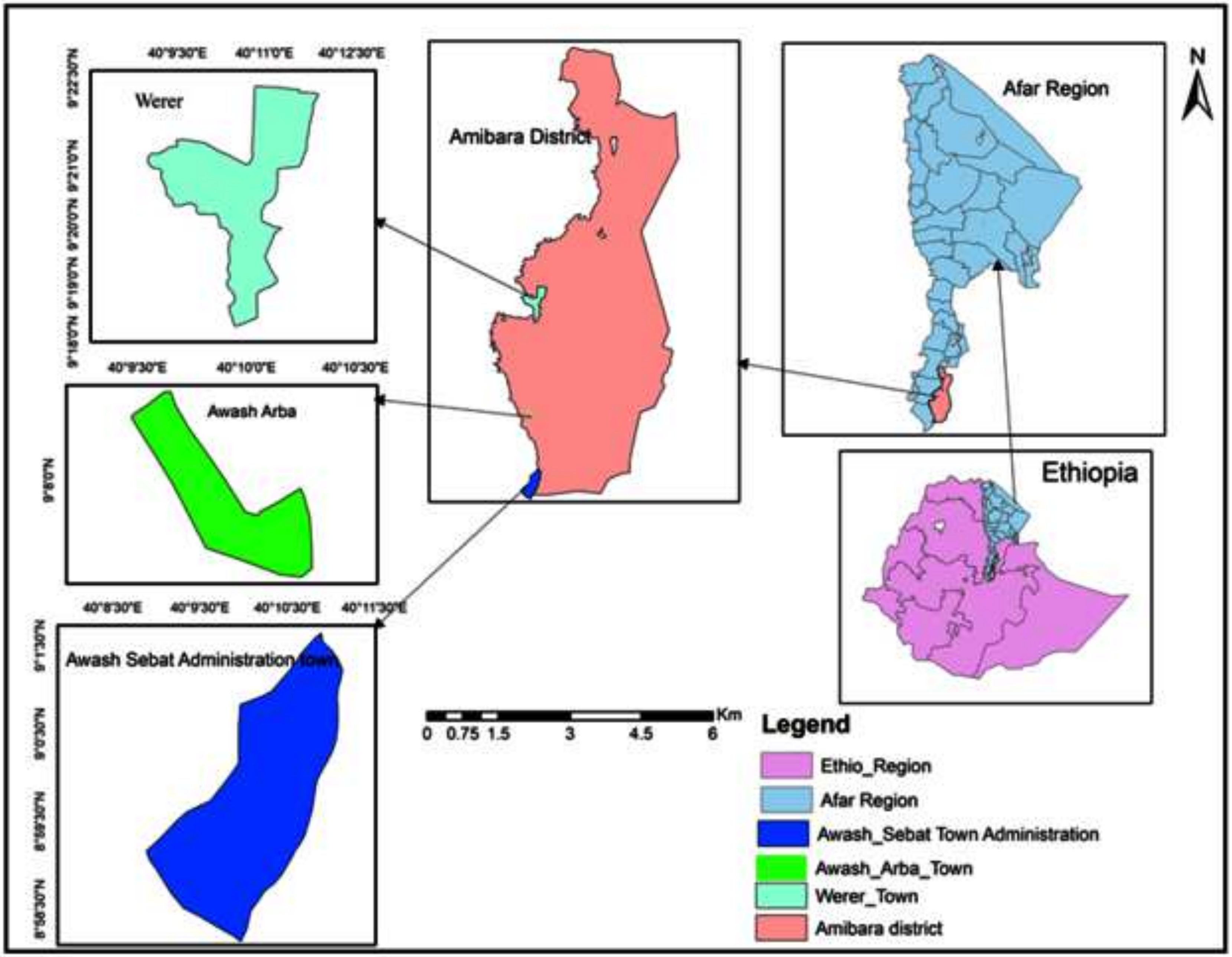

 Click here to access/download;Figure;Fig 2. tiff.tif

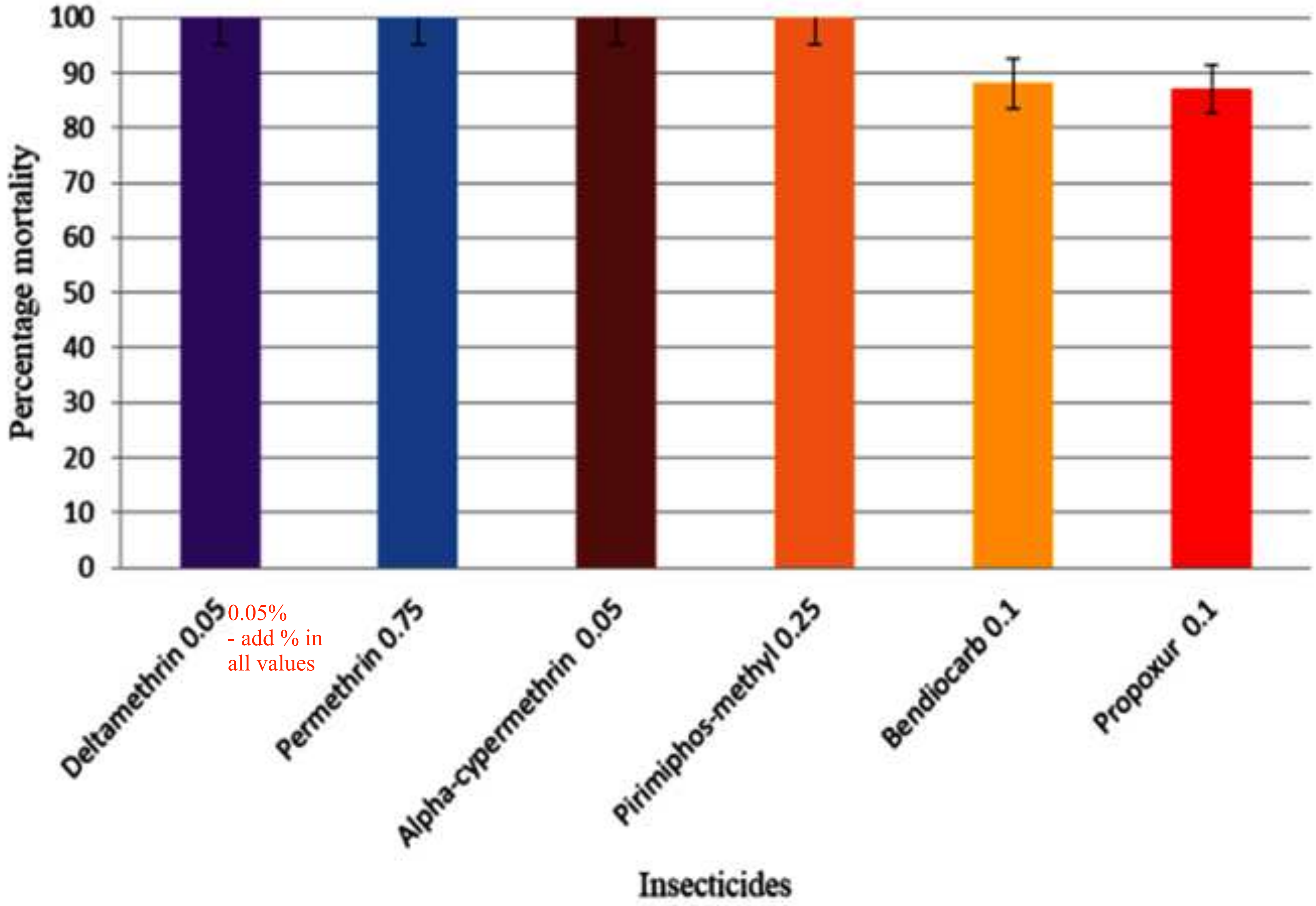

Click here to access/download;Figure;Fig 3. tiff.tif

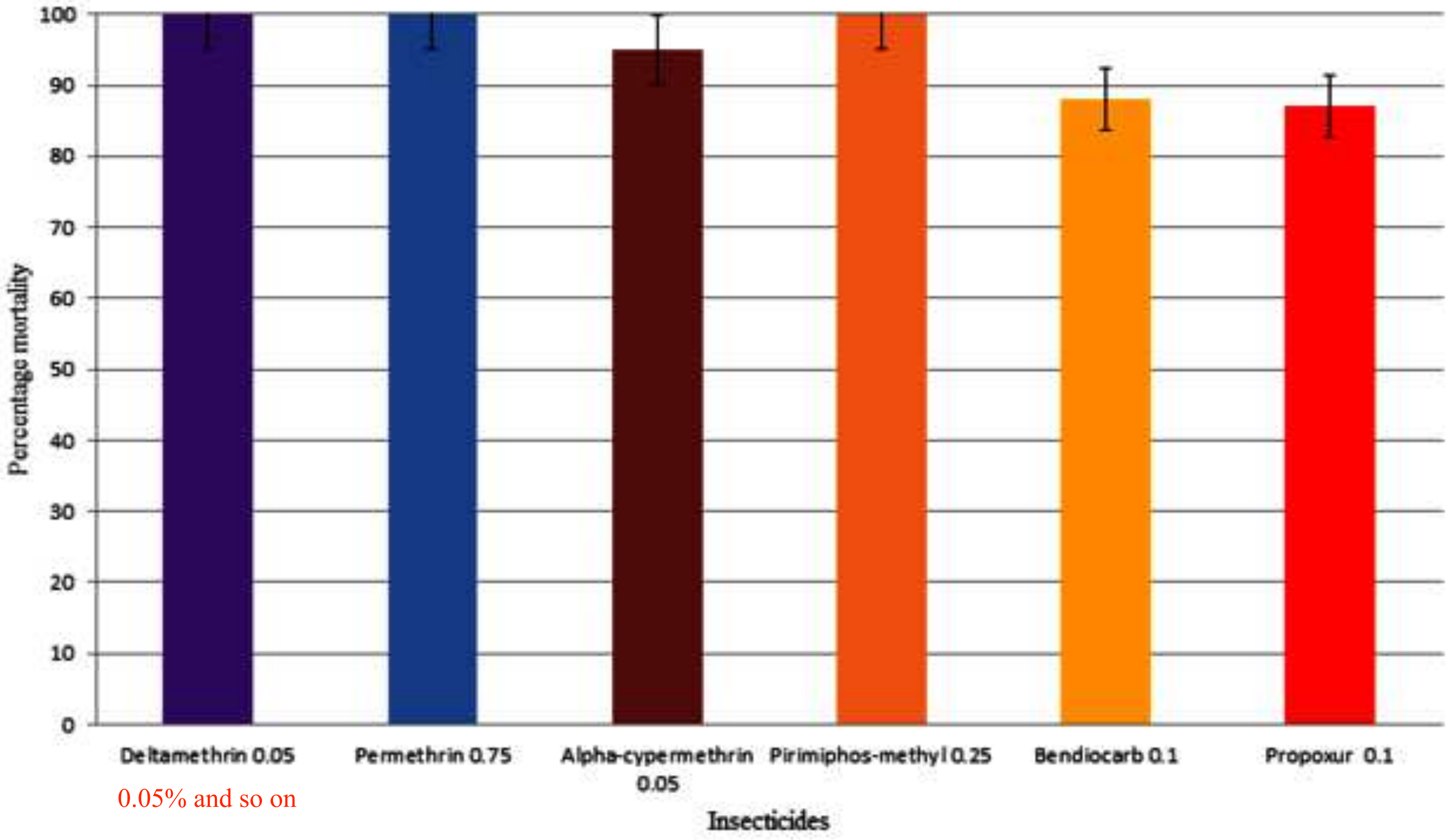

 Click here to access/download;Figure;Fig 4. tiff.tif

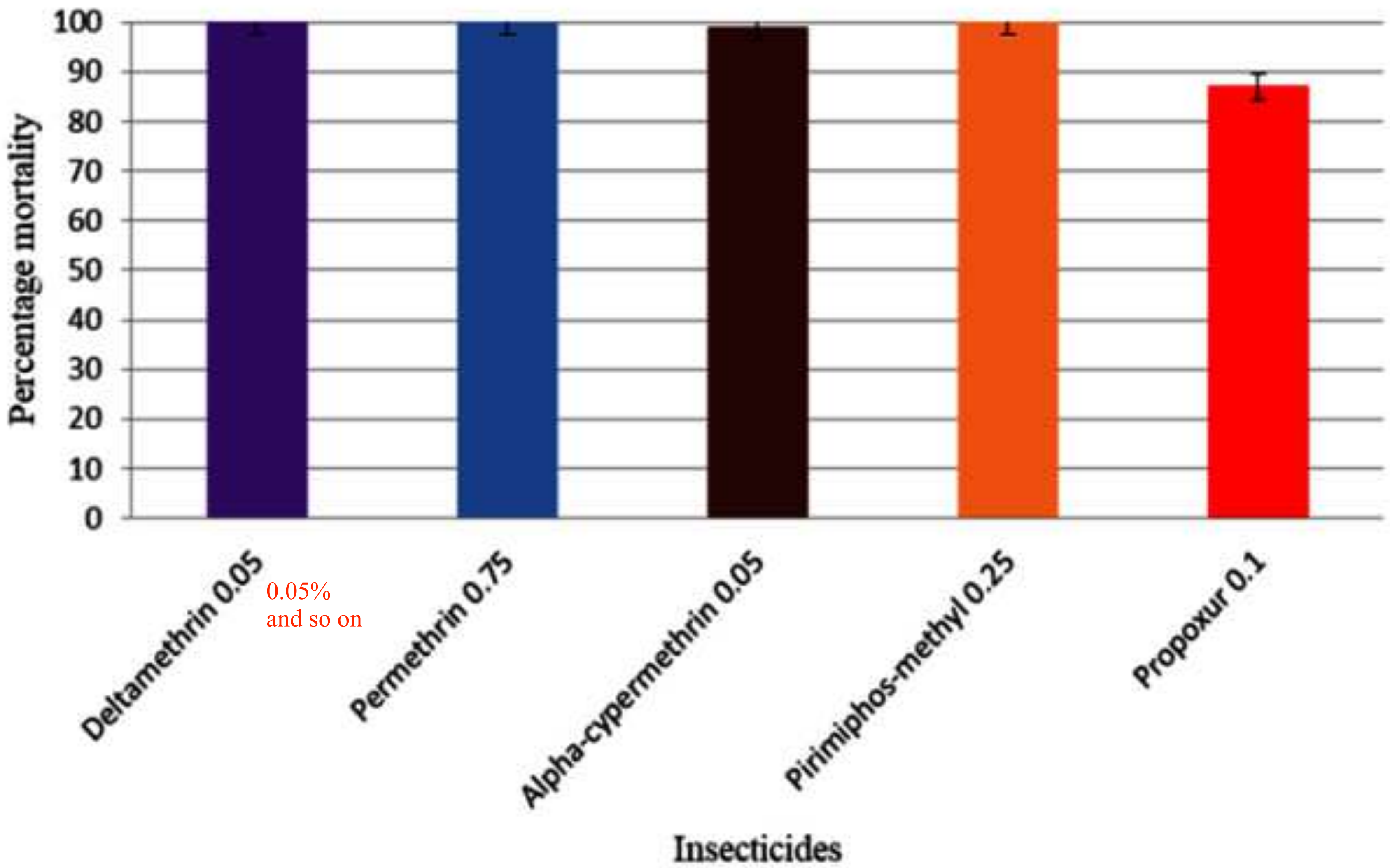

Click here to access/download
Supporting Information
S1_Table DOC.docx

Click here to access/download
Supporting Information
S2_Table DOC.docx

Click here to access/download
Supporting Information
S3_Table DOC.docx

Susceptibility status of *Aedes aegypti* (Diptera: Culicidae) to public health insecticides in Southern Afar region, Ethiopia

Mohammed Seid[1, 2*], Esayas Aklilu[1], Abebe Animut[1]

[1] Aklilu Lemma Institute of Pathobiology, Addis Ababa University, Addis Ababa, Ethiopia

[2] Department of Biology, College of Natural and Computational Science, Mattu University, Mattu, Ethiopia

*Corresponding author

Email: mohammed.seid.legas@gmail.com (MS)

Abstract

Background: Mosquito-borne viral diseases such as dengue fever, chikungunya and yellow fever have been documented in Ethiopia since the 1960s. However, efficacy of public health insecticides against *Aedes aegypti* that transmit these viruses remain poorly understood in the country particularly in the Afar region. Thus, the aim of this study was to assess the susceptibility status of *Ae. aegypti* to public health deltamethrin, permethrin, alpha-cypermethrin, pirimiphos-methyl, bendiocarb and propoxur insecticides.

Methodology: Larvae and pupae of *Aedes* mosquitoes were collected from the Awash Arba, Awash Sebat and Werer towns of the Afar Regional State of Ethiopia during July to October 2022, brought to Aklilu Lemma Institute of Pathobiology, Addis Ababa University Iinsectary and reared to adults under standard conditions. Non-blood fed 3 to 5 days old female *Ae.des aegypti* were exposed to insecticides that belonged to the pyrethroids, carbamates and organophosphate classes following the World Health Organization standard guidelines. Knockdown rates (KDT) were noted at 10 min interval until one hour. Mortality was recorded after 24 hours. The mortality in mosquitoes was recorded 24 hours after 60 minutes of exposure.

Results: *Aedes aegypti* from Awash Arba showed high KDT_{95} (26.5 minutes) *Aedes aegypti* from Awash Sebat showed high knock down time (KDT95 26.5 minutes) to deltamethrin compared to the *Ae.des aegypti* from other study sites. Mortalities of *Ae. aegypti* exposed to propoxur was 87% in all the study towns. Similarly, 88% of *Ae. aegypti* mortality was recorded when treated with bendiocarb in Awash Sebat and Awash Arba towns. Suspected re sistance of *Ae. aegypti* (95% mortality) exposed to alpha-cypermethrin were recorded from Awash Arba town. However, *Ae. aegypti* collected from all the three sites were observed to be susceptible to delthamethrin, permethrin, and pirimiphos-methyl.

Commented [u1]: Reviewer 2 comment

Commented [u2]: Reviewer 2

~~Conclusion~~: *Aedes aegypti* was resistant to 0.1% bendiocarb and 0.1% propoxur, and possibly resistant to 0.05% alpha-cypermethrin. On the other hand, it was susceptible to 0.05% delt~~h~~amethrin, 0.75% permethrin and 0.25% pirimiphos-~~ ~~methyl~~control~~. Thus, delt~~h~~amethrin, permethrin and pirimiphos-~~ ~~methyl can be used in the control of adult *Ae. aegypti* in the Afar Region of Ethiopia. However, further studies should be carried out to evaluate the susceptibility of *Ae. aegypti* to alpha-cypermethrin insecticide in the Awash Arba area.

~~**Key words:** *Aedes aegypti*, Afar, Ethiopia, Insecticides, Susceptibility~~

~~**Authors Summary**~~

~~Insecticide based mosquito control is an immediate and effective response during mosquito-borne viral diseases outbreak. However, mosquitos' resistance to insecticides adversely affect insecticide based control programmes. Insecticide resistance and possible resistance were observed in *Ae. aegypti* in the Southern Afar Regional State Ethiopia. On the other hand, *Ae. aegypti* was susceptible for delthamethrin, permethrin and pirimiphos methyl. Therefore, the Ethiopian Ministry of Health and Afar Regional Health Bureau can use these insecticides for the control of *Ae. aegypti*.~~

Introduction

The ever increasing outbreaks of arboviral diseases drew public attention in several countries of the world [1]. The East African countries including Kenya, Somalia, Djibouti, Eritrea and Ethiopia are epicentres of outbreaks of arboviral diseases such as dengue fever, yellow fever and chikungunya virus [2-4]. In Ethiopia, yellow fever caused about 30,000 deaths from over 200,000 infections during the 1960 and 1962 [5]. Recently, there have been yellow fever outbreaks in South Omo [6], dengue fever outbreaks in Dire Dawa city administration~~tive~~ ~~city~~, Somali Regional State, Gewane and Amibara districts of Afar Regional State [7, 8]. In addition, chikungunya outbreaks were also reported in Dire Dawa city administration~~city~~, Afar and Somali Regional States [9, 10]. All these outbreaks with potentially unrecognized and/or under reported deaths owing to the highly limited laboratory facilities in the country.

Mosquitoes transmit over 90% of the viral pathogens through their bites among which *Ae.*~~*des*~~ *aegypti* is the leading vector followed by *Ae. albopictus* [11, 12]. *Ae. aegypti* is anthropophilic in its behaviour and occurs in close proximity to human habitations [13, 14].

Commented [u3]: Reviewer 2 comment

Commented [u4]: Not part in the PLOS ONE Guideline

Ae. aegypti has two morphological subspecies (ecotypes) the domestic *Ae. aegypti aegypti* and sylvan *Ae. . aegypti formosus* [15, 16]. In Ethiopia, *Ae. aegypti*, *Ae. bromeliae*, *Ae. vittatus*, *Ae. hirsutus*, *Ae. simpsoni* complex and *Ae. Africanus* are implicated as the potential vectors of viral diseases [6, 8, 17-19].

Commented [u5]: Reviewer 2 comment

In the absence of effective therapeutic drugs and vaccines to treat and control mosquito borne viral diseases, preventions largely depend on vector control tools mainly public health insecticides [13]. The pyrethroids such as deltamethrin, alpha-cypermethrin, permethrin and organophosphates primiphos-—methyl and Ffenitrothion are among the public health insecticides used against *Aedes* mosquitoes [20]. However, the potential resistance to the insecticides may adversely affect implementation of the control strategy in endemic areas [21]. Mosquitoes could resist insecticides through their inherent ability to detoxify and avoid contacts either behaviourally or physiologically [22].

Evidences from many African countries indicate that the *Anopheles* and *Aedes* mosquitoes developed resistance to insecticides [23-25]. For instance in Ethiopia, malaria transmitting *Anopheles arabiensis* has developed resistance to insecticides in several areas of the country including in the Amibara district of the Afar Regional State [25, 26]. The causes for the resistance could be frequent use of the insecticide chemicals in indoor residual spraying and long-lasting insecticidal treated nets for the control of mosquito borne diseases in the area [21] or in argro-pesticides [27]. However, the susceptibility status of *Aedes* mosquitoes to insecticides currently used is lacking in the country.

Recently, the Ethiopian Public Health Institute prepared arboviral disease vectors, surveillance and control guideline in 2021 through chemical and environmental management methods. Vector control by chemical method such as fumigation, repellents and residual treatments were implemented in Dire Dawa city administrationve city and Somalia Regional State during dengue fever and chikungunya outbreaks (EPHI, 2021 unpublished). Unfortunately, there is a scarcity of evidences on the efficacy of the insecticides for the control of virus transmitting *Aedes* species in the Southern Afar Region in the presence of increased reports of viral diseases incidence and prevalence. Therefore, assessing the effectiveness of the existing public health insecticides to control *Aedes* species is crucial to strengthen the arboviral diseases prevention and control programs in the country. This inspired us to generate base line data in relation to the applicability of insecticide control tools in the prevention and control of virus transmitting *Aedes* mosquitoes in the area.

Therefore, the aim of the study was to assess the susceptibility status of *Ae. aegypti* to public health insecticides in the Southern part of the Afar Regional State of Ethiopia.

Materials and Methods

Study sites

The study was carried out in Awash Arba, Werer and Awash Sebat towns, Gabi-Rasu Zone (Zone 3), of Afar Regional State, Ethiopia. The Awash Arba and Werer towns are located about 250 km to the North-east of Addis Ababa at 9.33453°N latitude, 40.181385°E longitude and an altitude range of 720 metres above sea level (masl) to 1100 masl (Central statistics Agency, 2019 unpublished). Awash Arba and Werer sites were selected from Amibara district which are about 30 km distance far apart (Fig 1). Agro-ecologically, these two towns are semi-arid with temperature ranging from 25 °C to 35 °C, and average annual rainfall of 530 mm. The communities livelihoods are characterized as pastoralist and agro-pastoralist in which they mainly obtained income on livestock and from livestock and other agricultural crop production [28].

Awash Sebat town administration is located 214 km North-east of Addis Ababa at 8.98810°N latitude and 40.163936°E longitude (Central statistics Agency, 2019 unpublished). Its altitude ranges from 820 masl to 1120 masl and the temperature varies from 22.6 °C to 30.6 °C. The town and its surroundings receive a mean annual rainfall of 606.6 mm. ~~The study towns were selected in collaboration with the local district health professionals and on the basis of the recent outbreak reports of dengue fever and chikungunya (Awash Sebat town and Amibara district health bureaus, 2022 unpublished)~~.

The study areas are found in the middle Awash Valley, where infectious disease such as malaria, chikungunya and dengue fever are common [29]. Considerable attention has been given by the regional government to achieve better health in the region including the three towns [30]. The region has hospitals, health centres, clinics and health posts in different zones and districts. There are one hospital, one clinic and five health ~~centers~~centres and few private clinics. Insecticide such as deltamethrin, permethrin, alpha-cypermethrin, pirimiphos-methyl bendiocarb and propoxur are used as insecticide residual spraying and/or long lasting insecticide treated nets ~~is practiced~~ for the control of malaria and arboviral diseases in these towns [26, 31]. Similarly, agro-chemicals and agro-pesticides are used since maize and sorghum productions are common in these areas which is used to improve agricultural crop production [32].

Commented [u6]: Reviewer 3 comment

Commented [u7]: Moved to larval collection and raring section

Commented [u8]: Reviewer 2 comment

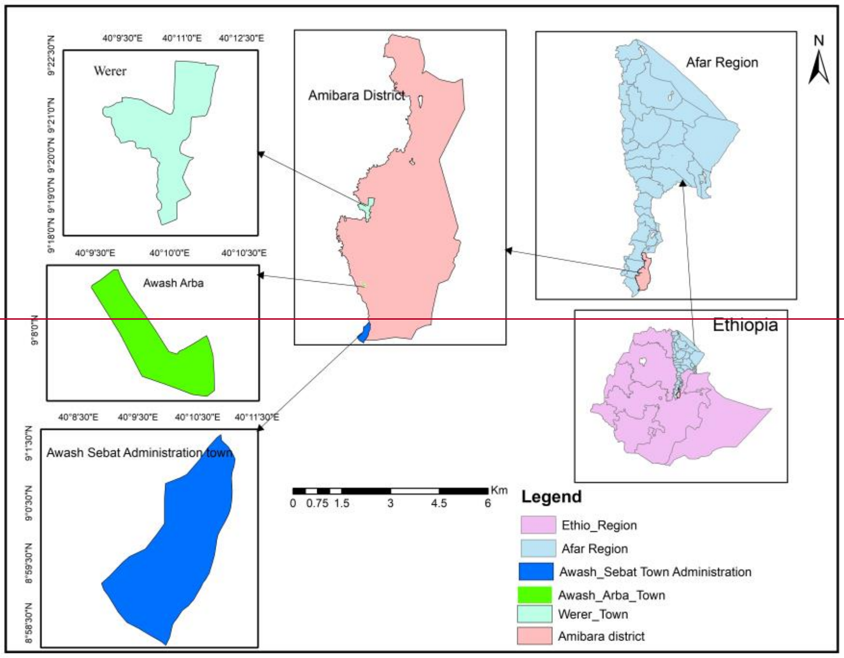

Fig 1. Map of the study areas. A map showing **the** ~~L~~locations of the three study areas: Awash Sebat, Awash Arba~~, Awash Sebat~~ and Werer Towns, in the Southern Afar Region, Ethiopia ~~(Source: Extracted from Ethio_GIS, 2023).~~

Larval collection and rearing

A cross-sectional study was designed to collect ~~L~~larvae and pupae of *Aedes* species from July to October, 2022 in the Awash Sebat, Awash Arba and Werer towns. The study towns were selected purposively in collaboration with the local district health professionals and on the basis of the recent outbreak reports of dengue fever and chikungunya (Awash Sebat town and Amibara district health bureaus, 2022 unpublished). The study sites were surveyed once per month for about 3-5 days. Larvae and pupae were collected from different positive habitats including tyres, water storage drums, discarded plastics and metallic containers using standard dipper (350 ml) and ladles and pipets/nets, ~~from July to October, 2022. The collected larvae were~~ ~~brought to~~transported to the insectary of Aklilu Lemma Institute of

Commented [u9]: Reviewer 3 comment

Pathobiology, Addis Ababa University, Ethiopia and reared to adults under standard laboratory conditions. The larvae were fed tTetra cichlid floating fish food (®/TM/©2019 Germany) was provided as food to the larvae. The emergence of larvae in to pupae was checked each morning and pupae were collected in glass flasks and transferred in to (30cm x 30cm x 30cm) netted cages. Emerged adult *Ae.des aegypti* were kept at 27 ± 2 °C temperature, 75 ± 10% relative humidity and fed on 10% sucrose solution until the bioassay tests. Emergent adults were identified to species using morphological taxonomic keys for *Aedes* species [33].

Phenotypic Insecticide susceptibility status of *Aedes aegypti*

Prior to insecticide susceptibility tests, sugar fed female *Ae. aegypti* were starved for 2hrs in order to prevent them from having high weight which leads them to be knocked down. Non-blood fed, 3-5 days old female *Ae. aegypti* were tested for their susceptibility to insecticides following the World Health Organization insecticide testing procedures[20](16). They were exposed to pyrethroids (0.05% deltamethrin, 0.05% alpha--cypermethrin, 0.75% permethrin), carbamates (0.1% bendio-carb,and 0.1% propoxur:) and organophosphates (0.25% pirimiphos--methyl) which are commonly used for preventing disease transmitting mosquitoes [20]. The insecticides were selected for the study because they have been used for the control of public health important mosquitoes in Ethiopia and the study area. The insecticides are also among the insecticide classes used in the country. Similar concentrations of insecticides were used to test the susceptibility of *Ae. aegypti* in Tanzania [34]. In each test, 20 female *Ae. aegypti* were exposed to each five treatment replicates and two control test tubes making a total of 140 mosquitoes in a single test. The exposed mosquitoes were observed for 60 minutes at an interval of 10 minutes to observe their knockdown. After an hour, the exposed females were transferred into holding tubes and kept for 24 h at 27.0 ± 2.0 °C and 75 ± 10% relative humidity and were offered cotton wool pads soaked in 10% sugar solutionfed on 10% sugar solution. The mortality in mosquitoes was recorded 24 hours after 60 minutes of exposure. Mortality was recorded after 24 hours. *Ae. aegypti* susceptibility test was repeated for alpha-cypermethrin in Awash Arba town since *Ae. aegypti* populations were appeared to be suspect phenotypic resistance to this insecticide. However, Iin case of Werer town the susceptibility status of *Ae. aegypti* to bendiocarb was not conducted due to insufficient mosquito samples.

Commented [u10]: Reviewer 2 comment

Ethical Issues

The study protocol obtained ethical approval from the institutional review board (IRB) of Aklilu Lemma Institute of Pathobiology, Addis Ababa University, Ethiopia (June 13, 2022; ALIPB IRB/80/2022). Written permission letters were obtained from the district health bureau and health centers of the towns. The households were informed about the objective of the study and their agreements were sought prior to the larvae/pupae collection.

Data analysis

Mortality was determined on the basis of the dead and alive mosquitoes after the 24 hours recovery period. Susceptible were those with a mortality in the range of 98-100%, suspected for resistance were with a mortality 90-97~~8~~% and resistant if mortality was <90% [20]~~(34)~~. The knockdown effect of pyrethroids against the *Ae. aegypti* were recorded during the 60 minutes exposure at an interval of 10 minutes from the start. Probit analysis was used to determine estimated ~~mean~~ duration at which 50% (KDT50) and 95% (KDT95) of the exposed *Ae. aegypti* mosquitoes were knocked down. Overall adult mortalities and knockdown times were computed using SPSS Software version 20.

Results

Knockdown effect of insecticides against *Aedes aegypti*

Knockdown time of *Ae. aegypti* exposed to insecticides was depicted in Table 1**.** The knock down effect of 50% (KDT$_{50}$) of *Ae. aegypti* exposed of deltamethrin ranged from 11.75 to 22.5 minutes whereas KDT$_{95}$ of the insecticide ranged from 18.75 to 26.5 minutes. Deltamethrin in *Ae. aegypti* showed high knockdown time (KDT$_{95}$ 26.5 minutes) in Awash Sebat as compared to other towns (Table 1). ~~*Ae. aegypti* from the Awash Arba showed high KDT$_{95}$ (26.5 minutes) to deltamethrin as compared to the *Ae. aegypti* from the other sites~~ (Table 1). On the other, *Ae. aegypti* exposed to permethrin showed the lowest KDT50 in Awash Arba. Overall, the KDT$_{50}$ and KDT$_{95}$ of *Ae. aegypti* varied among *Ae. aegypti* in knockdown to insecticides across the study localities.

Commented [u11]: Reviewer 2 comment

Table 1 Knockdown times of 50% and 95% of *Ae. a~~A~~egypti* exposed to commonly used insecticides in three localities from July to October 2022 in Southern Afar region Ethiopia

Commented [u12]: Reviewer 3 comment

Insecticides	Localities	KDT_{50} 95% [CI]	KDT_{95} 95% [CI]±SE
Deltamethrin	Awash sebat	22.5± 4.1617.06 [15.81-18.28]	26.5±4.5 34.01 [31.02-38.12]
	Awash Arba	11.75±10.4 12.15 [11.41-12.97]	17.5±0.518.74 [16.98-21.56]
	Werer	12.75±6.8416.53 [15.37-17.67]	18.75±0.1330.92 [28.24-34.62]
Permethrin	Awash Ssebat	11.25±3.4 8.92 [7.34-10.26]	15.2524.21 [21.23-28.93]
	Awash Arba	9±1.0 5.11 [2.91-6.94]	15.5±4.4 19.23 [16.23-24.09]
	Werer	12.25±2.59 15.12 [12.45-17.66]	15.5±0.5 29.89 [24.64-41.53]
Alpha-cypermethrin	Awash Ssebat	14±3.0 14.35 [13.35-15.35]	18±10.5 24.93 [22.77-27.97]
	Awash Arba	14.75±4.1213.47 [12.64-14.36]	16±1.0 20.78 [19.04-23.32]
	Werer	15.75±0.81 16.71 [13.85-19.46]	16.5±0.5 32.90 [27.26-44.98

CISE, Confidence interval standard error; KDT50, time taken for 50% of the tested *Ae. aegypti* to be knocked-down; KDT95 time taken for 95% of the tested *Ae. aegypti* to be knocked-down

Susceptibility status of *Aedes aegypti* to insecticides

The percentage mortalities of *Ae. aegypti* mosquito population against insecticides was presented in figures (figs. 2-4). Accordingly, *Ae. aegypti* mosquitoes showed phenotypic resistance to bendiocarb and propoxur insecticides in which the percentage mortalities were recorded to be 88% and 87% respectively in Awash Sebat town. *Ae. aegypti* mortalities to deltamethrin, permethrin alph-acypermthrin and pirimiphos-–methyl were 100% in Awash sSebat town (Fig. 2). All mosquitoes in the control group showed 0% mortality in all study sites.

Commented [u13]: We analysed using 95% of confidence interval.

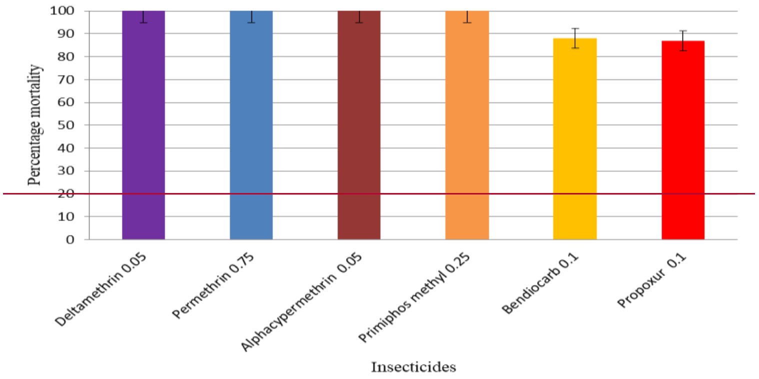

Fig 2. Insecticide susceptibility status of *Ae. aegypti* populations from Awash Sebat town, Southern Afar region, Ethiopia, from July to October 2022. (Error bars represent 95% confidence intervals).

In addition, *Ae. aegypti* mosquitoes were also observed resistance to bendiocarb and propoxur from Awash Arba. Moreover,Similarly *Ae. aegypti* mosquitoes were observed to be suspected resistant to alpha-cypermethrin in which the mortality of *Ae. aegypti* mosquitoes due to alpha-cypermethrin was 95% (Fig 3). *Ae. eagypti* mortalities were observed to be 100% when exposed to of *Ae. aegypti* mosquitoes mortalities were recorded in Awash Arba to deltamethrin, permethrin and pirimiphos--methyl insecticides in this town.

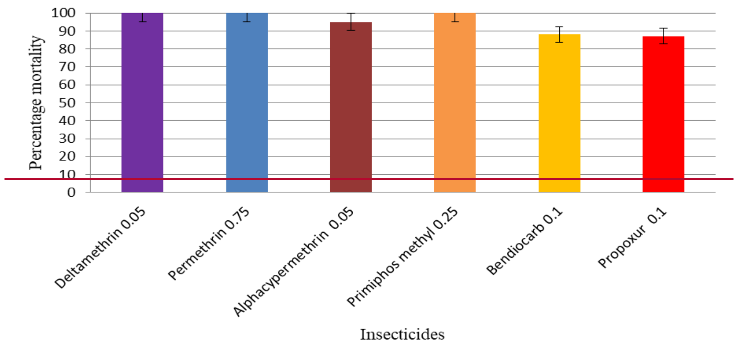

Fig 3. Insecticide susceptibility status of *Ae. aegypti* populations from Awash Arba town, Southern Afar region, Ethiopia, from July to October 2022. (Error bars represent 95% confidence intervals).

Ae. aegypti mortalities when exposed to propoxur insecticide was About 87% whereas and 99% mortalities were observed among the *Ae. aegypti* mosquitoes from Werer Werer town when treated with exposed to propoxur and alpha-cypermethrin insecticides respectively (fig. 4). However, 100% mortalities of *Ae. aegypti* from this site were recorded to deltamethrin, permethrin and pirimiphos--methyl.

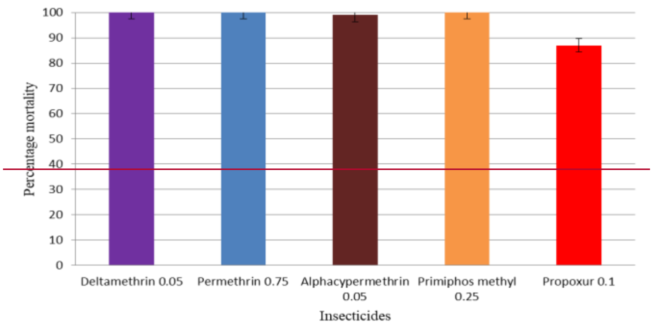

Fig 4. Insecticide susceptibility status of Adult *Ae.aegypti* populations in Werer town, Southern Afar region, Ethiopia, from July to October 2022. (Error bars represent 95% confidence intervals).

Discussion

Recent emergence and re-emergenceoutbreaks of dengue fever and chikungunya virus fever arboviral diseases in the Eastern and the North--aEastern parts of the Ethiopia country including especially in Dire Dawa city administrationve city, Somali Regional State and Amibara and Gewane districts of Afar regional State requires the need for the control of the *Aedes* species that transmit the viruses. The insecticide based mosquito control is underway in Ethiopia in general and calls an alarm to confirm the efficacy of mortality of the public health insecticides in Afar regional State in particular and elsewhere in the country [7, 35]. HoweverIn so doing there have been continuous evaluation of efficacy of the insecticides against malaria transmitting *Anopheles* species but there is scarcity of research findings conducted to control *Aedes* species either chemically, biologically or other means of vector control strategies in the country [36]. Thus, the control of viral disease transmitting *Aedes aegypti* mosquitos' vector is poorly investigated and needs detailed research. Major focus was given to the malaria vector and there is the scarcity of research findings conducted to control *Aedes* mosquitoes either chemically, biologically or other means of vector control in

the country [36]. As far as our knowledge is concerned, this study was first in its kind to report the susceptibility status of *Ae. aegypti* to public health chemical insecticides in the sSouthern Afar rRegional State and there are limited data to compare our findings to others in the country.

Aedes. aegypti is generally considered as exophillic, exophagic and day time bitters. They usually feed on human and other vertebrate hosts as described in previous studies [37, 38]. However, they may tend to be endophilic and endophagic in which they rest and fed indoors [39]. This makes controlling of the *Ae.des*aegypti mosquitoes and the viral diseases they transmitted more difficult [40]. As a result, different intervention tools have been adopted to control these mosquitoes including indoor residual insecticide spraying and insecticide impregnated long lasting insecticide treated nets in addition to environmental managingement by removing *Aedes* their breeding habitats [41].

The present study revealed that the *Ae. aegypti* KDT$_{50}$ and KDT$_{95}$ for deltamethrin, ranged from 11.25-22.5 minutes and 15.25-26.5 minutes recorded in the study towns. This knock down results were highest as compared to other pyrethroids in the study areas. On the other hand, In the present study *Ae. aegypti* were susceptible to deltamethrin in Sudan and Tanzania but suspected resistance in Nigeria. Similarly, in the current study, *Ae. aegypti* showed the higher lowest KDT$_{50}$ and KDT$_{95}$ as compared to the study conducted in Sudan to deltamethrin insecticide. For instance, the KDT$_{50}$ and KDT$_{95}$ recorded for deltamethrin in Sudan were 13 and 19 minutes respectively which were slightly lower than the current study in Awash Sebat [42]. However, permethrin as compared study conducted in Nigeria revealed that the KDT$_{50}$ of deltamethrin was two times higher than the KDT$_{50}$ results observed in Awash Sebat[43]. Relatively similar permethrin KDT$_{50}$ results were observed in Awash Arba town and Mlabani, Tanzania. However, higher deltamethrin and permethrin KDT$_{95}$ results were observed in Mlabani than all the present study towns [44]. to delthamethrin and alphacypermethrin in Awah town. Relatively high tolerance of deltamethrin observed in the present study areas may be attributable to its frequent use in the insecticide treated nets. However, the findings observed in the present study were lower than results reported in Sudan, Tanzania and Nigeria [34, 42, 43]. Overall, the tube test revealed that *Ae. aegypti* were susceptible to deltamethrin in Sudan and Tanzania but suspected resistance in Nigeria.

Commented [u14]: Reviewer 3 comments

Ae. aegypti varied in their response to public health insecticides. In the present study, *Ae. aegypti* were observed to be phenotypic resistant to bendiocarb where the mortality rates were less than 90%. *Ae. aegypti* resistance to bendiocarb was also reported in a previous

study in Tanzania [34]. However, these finding were in contrary to the finding reported from Sudan, Nigeria and Cambodia [42-44]. World health organization in 2022 has recommended that the insecticide discriminating dose of bendiocarb for *Ae. aegypti* is changed from its initial 0.1% to 0.2% [45]. Thus, future efficacy studies of endiocarb should target the 0.2% concentration. The variation in the findings from the present study may be due to ecological factors in addition to the concentration difference of the insecticides.

In the present study, *Ae. aegypti* mortally against propoxur insecticide was assessed. It was observed that *Ae. aegypti* mortality when exposed to propoxur showed insecticide resistance in all the study towns. Similar findings were reported in Thailand where the efficacy of *Ae. aegypti* mortality to propoxur was 83% which indicated the phenotypic resistance of *Ae. aegypti* to propoxur in this area [46].

The possible contributing factors to the emergences of phenotypic resistance of *Ae. aegypti* Mosquitoes in the present study areas could be as a disease vector emerge insecticide resistance to commonly used public health insecticides possibly one of the following ways. First, frequent uses of insecticide chemicals such as insecticide treated bed net and indoor residual insecticide spraying to control mosquito borne diseases may lead to mosquitoes to develop insecticide resistance gene as previously indicated [47]. On the other hand, Ethiopia has long history in utilizing pesticides to increase agricultural production and to improve human health since 1964 [48, 49]. Unfortunately however, agro-pesticides induce insecticides resistance selection pressure in different ways. For instance, insecticides used to control mosquitoes can counteract to agro-pesticides used which have the same mode of action and bio-active ingredients might drive a selection pressure to chemical based insecticide control tools [27]. Similarly, agro-pesticide used in agriculture may ease insecticide tolerance as the pesticides washed to mosquito breeding habitats which confer resistance mechanism at the adult stages [50]. Thus, the emergence of insecticide resistance in bendiocarb and propoxur insecticides in the present study could potentially arise from the frequent exposer of the mosquito to carbamate insecticides in agriculture used as an agro-pesticides or herbicides. now a days agro-pesticide are now widely used to improve agricultural crop production. Unfortunately however, agro-pesticides induce insecticides resistance selection pressure in different ways. For instance, insecticides used to control mosquitoes can counteract to agro-pesticides used which have the same mode of action may drive a selection pressure to chemical based insecticide control tools [27]. Similarly, agro-pesticide used in agriculture

Commented [u15]: Reviewer 2 comment

Commented [u16]: Reviewer 3 comment

Formatted: Font: Italic

Commented [u17]: Based on reviewer 3's comment

may ease insecticide tolerance as the pesticides washed to mosquito breeding habitats which confer resistance mechanism at the adult stages [50].

Ethiopia has long history in utilizing pesticides to increase agricultural production and to improve human health since 1964 [48, 49]. Thus, the emergence of insecticide resistance in bendiocarb and propoxur insecticides in the present study could potentially arise from the frequent exposer of the mosquito to carbamate insecticides in agriculture used as an agro-pesticides or herbicides. *Ae. aegpti* resistance to propoxur and bendiocarb may occur as a result of mutation in the insecticide target site enzyme Acetylcholinesterase (AChE) as reported in Cuba [51].

Commented [u18]: Reviewer 3 comment

The pyerethroids such as deltamethrin are chemical insecticides commonly used to control malaria and other mosquito borne diseases in Ethiopia. In the present study, the pyrethroid alpha-cypermethrin showed less than 97% of mortality of adult *Ae. aegypti* populations in the Awash Arba town which revealed the emergence of possible resistance in *Ae. aegypti* mosquito population in this area. The emergence of possible resistance of *Ae. aegypti* mosquitoes in Awash Arba may be the frequent use of pyrethroids in the insecticide treated nets to control malaria in this area and agro-pesticides as discussed above. This may lead failer to vector control interventions programs in the study area and elsewhere in the country. Similar findings were reported in Ghana [23].

In the present study, Ccomplete susceptibility of *Ae. aegypti* mosquito populations were recorded to the organophosphate chemical insecticide i.e. pirimiphos--methyl in all the study areas. Likewise, the study conducted in Cambodia [44] reported similar findings in that the adult *Ae. aegypti* populations were completely susceptible for the pirimiphos--methyl even in its lower concentration than in the present study. However, this finding was in contrary to the findings reported in Nigeria [43]. Deltamethrin and perimethrin of the pyrethroid insecticides are also showed 100% mortality to adult *Ae. aegypti* mosquito populations in the study areas. These findings were in contrary to the findings reported in Malaysia [52] where resistance phenotypes were observed in permethrin and deltamethrin. However, sSimilar findings was reported in Tanzania [34] and in Peru [53] where deltamethrin showed greater than 99% of mortality in adult *Ae. aegypti*. Thus, this findings could serve as a base line to the Ethiopian ministry of health and the Afar region health bureau to implement the chemical interventions particularly the pyrethroids deltamethrin, permethrin and the organophosphate pirimiphos-methyl either in the form of aerial spray or surface treatment to reduce the burden of arboviral diseases during diseases outbreak in different parts of the country.

Conclusion

The present study reported that *Ae. aegypti* populations showed insecticide resistance to bendiocarb and propoxur and possible resistance to alpha-cypermethrin and complete susceptibility to deltamethrin, per~~i~~methrin and pirimiphos-~~-~~methyl insecticides. Thus, ~~From the finding of the present study,~~ based on the findings, the following points would be forwarded. Continuous surveillances of *Aedes* species susceptibility to insecticides ~~Further study~~ should be ~~inspired on insecticide resistance of *Aedes* mosquitoes~~ carried out in the study areas and elsewhere in Ethiopia including molecular aspects to mark and evaluate resistance pattern. Moreover, proper uses of existing insecticides are mandatory to hinder *Ae. aegypti* mosquito resistance to insecticides in the study areas. In addition, ~~integrated vector control strategies should be developed in the study areas and other arboviral endemic areas of Ethiopia. Finally,~~ the organophosphate insecticide pirimiphos-~~-~~methyl can be used in addition to deltamethrin and permethrin in~~for~~ the control of adult *Ae. aegypti* mosquitoes in Ethiopia.

Acknowledgement

The authors would like to acknowledge Aklilu Lemma Institute of Pathobiology Addis Ababa University, Ethiopia for providing us field and laboratory materials and insecticide impregnated papers. We also like to gratitude the vector biology and control units staffs, ~~Mr.~~Mr Wosen Sisay for his technical assistance in the insectary. Finally, we appreciated all the communities and data collectors from Awash Sebat, Awash Arba and Werer towns for their collaboration during the study period.

Author contributions

Conceptualization: Mohammed Seid, Esayas Aklilu and Abebe Animut

Data curation: Mohammed Seid

Formal Analysis: Mohammed Seid

Investigations: Mohammed Seid, Esayas Aklilu and Abebe Animut

Methodology: Mohammed Seid, Esayas Aklilu and Abebe Animut

Supper-vision: Mohammed Seid, Esayas Aklilu and Abebe Animut

Validation: Mohammed Seid, Esayas Aklilu and Abebe Animut

Visualization: Mohammed Seid, Esayas Aklilu and Abebe Animut

Writing original draft: Mohammed Seid

Writing, reviewing and editing: Mohammed Seid, Esayas Aklilu and Abebe Animut

References

1. Braack L, et al. Mosquito-borne arboviruses of African origin: review of key viruses and vectors. Parasit~~es & ~~vectors. 2018; 11(1): 1-26. doi: 10.1186/s13071-017-2559-9 PMID: 29316963
2. Langat SK, et al. Origin and evolution of dengue virus type 2 causing outbreaks in Kenya: Evidence of circulation of two cosmopolitan genotype lineages. Virus Evolution. 2020; 6(1):1-9. doi: 10.1093/ve/veaa026 PMID: 32523778
3. Gutu MA, et al. Another dengue fever outbreak in Eastern Ethiopia-An emerging public health threat. PLoS Negl~~ected~~ Trop~~ical~~ Dis.~~eases~~ 2021; 15(1):1-16. doi:10.1371/journal.pntd.0008992 PMID: 33465086
4. Usman A, et al. Dengue fever outbreaks in Eritrea, 2005-2015. Global health research and policy 2016; 1(1): p. 1-8. doi: 10.1186/s41256-016-0016-5 PMID: 29202065
5. Serie C, et al. Studies on yellow fever in Ethiopia. Epidemiologic study. Bulletin of the World Health Organization. 1968; 38(6):879-884. PMID: 53036636
6. Lilay A, et al. Re-emergence of yellow fever in Ethiopia after 50 years, 2013: epidemiological and entomological investigations. BMC ~~i~~Infect~~ious~~ ~~d~~Dis.~~eases~~ 2017; 17(1):1-6. doi: 10.1186/s12879-017-2435-4 PMID: 28506254
7. Woyessa AB, et al. The first acute febrile illness investigation associated with dengue fever in Ethiopia, 2013: a descriptive analysis. The Ethiop~~ian~~ J~~ournal~~ ~~of~~ Health Develop.~~ment~~ 2014; 28(3):155-161.
8. Mekuriaw W, et al. Epidemiological, Entomological~~,~~ and Climatological Investigation of the 2019 Dengue Fever Outbreak in Gewane District, Afar Region, North-East Ethiopia. Insects. 2022; 13(11):1-13. doi: 10.3390/insects13111066 PMID: 36421969
9. Geleta D, et al. Epidemiological Description of Chikungunya Virus Outbreak in Dire Dawa Administrative City, Western Ethiopia, 2019. Int J Clin Exp Med Sci. 2020; 6(3): 41-45. doi: 10.11648/j.ijcems.20200603.13
10. Alayu M, et al. Risk factors for Chikungunya outbreak in Kebridhar City, Somali Ethiopia, 2019. Unmatched case-control study. bioRxiv. 2020:1-20.
11. Gubler DJ. Dengue, urbanization and globalization: the unholy trinity of the 21st century. Trop~~ical~~ ~~m~~Med~~icine~~ ~~and~~ ~~h~~Health. 2011; 39(4SUPPLEMENT):1-12. doi: 10.2149/tmh.2011-S05 PMID: 22500131.12.

12. Li Y, et al. Urbanization increases *Aedes albopictus* larval habitats and accelerates mosquito development and survivorship. PLoS nNeglected tTropical Ddis.eases 2014; 8(11): 1-12. doi: 10.1371/journal.pntd.0003301 PMID: 25393814
13. WHO. Dengue: guidelines for diagnosis, treatment, prevention and control. World Health Organization. 2009;1-148. PMID: 2376296314
14. Powell JR. Mosquito-borne human viral diseases: why *Aedes aegypti*? The American Jjournal of tTropical mMedicine and Hhyg.iene 2018; 98(6):1563–1565. doi: 10.4269/ajtmh.17-0866 PMID: 29557341
15. Mattingly P. Taxonomy of *Aedes aegypti* and related species. Bulletin of the World Health Organization. 1967;36(4): 552-554. PMID: 438354416
16. Rose NH, et al. Climate and urbanization drive mosquito preference for humans. Current Biol.ogy, 2020; 30(18): 1-29. doi: 10.1016/j.cub.2020.06.092 PMID: 32707056

17. Getachew D, et al. Breeding sites of *Aedes aegypti*: potential dengue vectors in Dire Dawa, East Ethiopia. Interdisciplinary pPerspectives on iInfectious Ddis.eases 2015; 2015:1-8. doi: 10.1155/2015/706276 PMID: 2643571218
18. Ferede G, et al. Distribution and larval breeding habitats of *Aedes* mosquito species in residential areas of northwest Ethiopia. Epidemiology and Hhealth. 2018; 40: 1-7. doi: 10.4178/epih.e2018015 PMID: 29748457
19. Woyessa A, et al. *Aedes* Mosquitoes Distribution and Risk of Yellow fever transmission in Gurage Zone South-west Ethiopia. Ethiopian Journal of Public Health and Nutrition. 4(1):1-10.
20. WHO. Monitoring and managing insecticide resistance in *Aedes* mosquito populations: interim guidance for entomologists. 2016;1-11.
21. Moyes CL, et al. Contemporary status of insecticide resistance in the major *Aedes* vectors of arboviruses infecting humans. PLoS Nneglected tTropical dDis.eases, 2017. 11(7): 1-20. doi: 10.1371/journal.pntd.0005625 PMID: 28727779
22. WHO. Global plan for insecticide resistance management in malaria vectors. 2012;13-132.
23. Owusu-Asenso CM, et al. Spatiotemporal distribution and insecticide resistance status of *Aedes aegypti* in Ghana. Parasites & Vectors. 2022; 15(1):1-14. doi: 10.1186/s13071-022-05179-w PMID: 35183249

24. Mathias L, et al. Habitat productivity and pyrethroid susceptibility status of *Aedes aegypti* mosquitoes in Dar es Salaam, Tanzania. Infect~~ious~~ Dis~~eases~~ ~~of~~ Poverty. 2017; 6(1):1-10. doi: 10.1186/s40249-017-0316-0 PMID: 28595653

25. Kenea O, et al. Impact of combining indoor residual spraying and long-lasting insecticidal nets on *Anopheles arabiensis* in Ethiopia: results from a cluster randomized controlled trial. Malaria J.~~ournal~~ 2019; 18(1):1-11. doi: 10.1186/s12936-019-2811-1 PMID: 31126286

26. Messenger LA, et al. Insecticide resistance in Anopheles arabiensis from Ethiopia (2012–2016): a nationwide study for insecticide resistance monitoring. Malaria J.~~journal~~ 2017; 16(1):1-14. doi: 10.1186/s12936-017-2115-2 PMID: 29151024

27. Nkya TE, et al. Impact of environment on mosquito response to pyrethroid insecticides: facts, evidences and prospects. Insect biochemistry and molecular biol.~~ogy~~ 2013; 43(4):407-416. DOI: 10.1016/j.ibmb.2012.10.006 PMID: 23123179

28. Mekuyie M, Jordaan A and Melka Y. Understanding resilience of pastoralists to climate change and variability in the Southern Afar Region, Ethiopia. Climate Risk Managt.~~ement~~ 2018; 20: 64-77.

29. Gebru H, Ahmed J. Water quality assessments as implication to human health: a case study in awash sebat and its surrounding areas, Afar, Ethiopia. Inter~~national~~ J~~ournal~~ ~~of~~ Earth Sci~~ences~~ and Engi.~~neering~~ 2016; 9(5):1999-2008.

30. Goshu D, et al. Socioeconomic Development in Afar Region: Achievements, Gaps and Priorities. 2021.

31. Ministry of Health Ethiopia. Vector control operational manual for malaria elimination in Ethiopia. 2017. 1-56.

32. Sileshi G, Zemedkun A, Nurhussien S. Assessment of the pesticide use, practice and hazards on cotton growing farmers in the Awash Valley Ethiopia. AgriRxiv.~~,~~ 2021(2021): 1-22. doi: https://cabidigitallibrary.org

33. Rueda LM. Pictorial keys for the identification of mosquitoes (Diptera: Culicidae) associated with dengue virus transmission. 2004, Walter Reed Army Inst Of Research Washington Dc Department of Entomology. 1-60. doi: 10.11646/zootaxa.589.1.1

34. Kahamba NF, et al. Habitat characteristics and insecticide susceptibility of *Aedes aegypti* in the Ifakara area, south-eastern Tanzania. Parasit~~es & ~~Vectors. 2020; 13(1) 1-15. doi: 10.1186/s13071-020-3920-y PMID: 32033619

35. Ahmed YM, Salah AA. Epidemiology of dengue fever in Ethiopian Somali region: retrospective health facility based study. Cent Afr J Public Health. 2016; 2(2):51-6.

36. Waldetensai A, et al. *Aedes* mosquito responses to control interventions against the Chikungunya outbreak of Dire Dawa, Eastern Ethiopia. Inter~~national~~ J~~ournal of~~ Trop~~ical~~ Insect Sci.~~ence~~ 2021;1-10.

37. Diouf B, et al. Resting behavior of blood-fed females and host feeding preferences of *Aedes aegypti* (Diptera: Culicidae) morphological forms in Senegal. J~~ournal of~~ Med~~ical~~ Entomol.~~ogy~~ 2021; 58(6):2467-2473. doi: 10.1093/jme/tjab111 PMID: 34165556

38. Ponlawat A and Harrington LC. Blood feeding patterns of *Aedes aegypti* and *Aedes albopictus* in Thailand. J~~ournal of~~ M~~m~~ed~~ical~~ E~~e~~ntomol.~~ogy~~ 2005; 42(5):844-849. doi: 10.1093/jmedent/42.5.844 PMID: 16363170

39. Diallo D and Diallo M. Resting behavior of Aedes aegypti in southeastern Senegal. Parasit ~~es~~Vectors. 2020; 13(1):1-7. doi: 10.1186/s13071-020-04223-x PMID: 32682436

40. Bartlow AW, et al. Forecasting zoonotic infectious disease response to climate change: mosquito vectors and a changing environment. Veter~~inary~~ S~~s~~ci.~~ences,~~ 2019; 6(2): 1.20. doi: 10.3390/vetsci6020040 PMID: 31064099

41. Lima EP, MOF Goulart and Rolim Neto ML. Meta-analysis of studies on chemical, physical and biological agents in the control of *Aedes aegypti*. BMC P~~p~~ublic H~~h~~ealth. 2015; 15:1-14. doi: 10.1186/s12889-015-2199-y PMID: 26341708

42. Husham A, Abdalmagid M and Brair M. Status susceptibility of dengue vector; *Aedes aegypti* to different groups of insecticides in Port Sudan City-Red Sea State. Sudanese J~~ournal of~~ Public Health. 2010; 5(4):199-202.

43. Ukpai OM, Ekedo CM. Insecticide susceptibility status of *Aedes aegypti* in umudike, Ikwuano lga Abia State, Nigeria. Animal Research Internationa. l2018; 15(3):3082-3089.

44. Boyer S, et al. Monitoring insecticide resistance of adult and larval Aedes aegypti (Diptera: Culicidae) in Phnom Penh, Cambodia. Parasit~~es &~~ Vectors. 2022; 15(1): 1-7. DOI: 10.1186/s13071-022-05156-3 PMID: 35101104

45. WHO. Manual for monitoring insecticide resistance in mosquito vectors and selecting appropriate interventions. World Health Organization. 2022; 1-55.

46. Pethuan S, et al. Biochemical studies of insecticide resistance in *Aedes* (Stegomyia) *aegypti* and *Aedes* (Stegomyia) *albopictus* (Diptera: Culicidae) in Thailand. Trop Biomed.~~,~~ 2007; 24(1):7-15. PMID: 17568372

47. Barbosa S, et al. Modelling the impact of insecticide-based control interventions on the evolution of insecticide resistance and disease transmission. Parasit~~es & V~~V~~v~~ectors. 2018; 11:1-21. doi: 10.1186/s13071-018-3025-z PMID: 30153869

Field Code Changed

48. Mengistie B. Policy-practice nexus: pesticide registration, distribution and use in Ethiopia. SM J. Environ~~.~~ Toxicol. 2016; 2(1):1006-1019.

49. Negatu B, Dugassa S and Mekonnen Y. Environmental and health risks of pesticide use in Ethiopia. J~~ournal of~~ Health Pollution. 2021; 11(30):1-12. doi: 10.5696/2156-9614-11.30.210601 PMID: 34267988 ~~p. 210601.~~

Field Code Changed

50. Poupardin R, et al. Cross-induction of detoxification genes by environmental xenobiotics and insecticides in the mosquito Aedes aegypti: impact on larval tolerance to chemical insecticides. Insect ~~b~~Biochemistry and ~~m~~Molecular ~~b~~Biol.~~ogy~~ 2008; 38(5): 540-551. doi: 10.1016/j.ibmb.2008.01.004 PMID: 18405832

Field Code Changed

51. Bisset J, Rodríguez MM and Fernández D. Selection of insensitive acetylcholinesterase as a resistance mechanism in *Aedes aegypti* (Diptera: Culicidae) from Santiago de Cuba. J~~ournal of~~ Med~~ical~~ Entomol.~~ogy,~~ 2006; 43(6):1185-1189. PMID: 17162951

52. Ishak IH, et al. Contrasting patterns of insecticide resistance and knockdown resistance (kdr) in the dengue vectors *Aedes aegypti* and *Aedes albopictus* from Malaysia. Parasit~~es & v~~Vectors. 2015; 8(1): 1-13. doi: 10.1186/s13071-015-0797-2 PMID: 25888775

Field Code Changed

53. Pinto J, et al. Susceptibility to insecticides and resistance mechanisms in three populations of *Aedes aegypti* from Peru. Parasites & vectors 2019; 12(1):1-11. doi: 10.1186/s13071-019-3739-6 PMID: 31640810

Field Code Changed

Supporting information

S1 Table. Observed knockdown times (in minutes) and number of knocked-down female *Ae. aegypti* exposed to insecticides during the 60 minutes in Awash Sebat, Awash Arba and Werer towns of Southern Afar, July to October 2022.

S2 Table. Female *Ae. aegypti* mortalities exposed to insecticides observed after 24 hrs of recovery period from Awash Sebat, Awash Arba and Werer towns, Southern Afar Regional State, Ethiopia, July to October, 2022.

S3 Table. Representative GPS points of the Awash Sebat, Awash Arba and Werer towns of Southern Afar Regional State, Ethiopia recorded during *Ae. aegypti* larvae and pupae collection.

Date 29/04/2024

To: James Colborn

Academic editor

PLOS ONE Journal

Sub: Providing point-by-point responses to the Editor's and reviewers' comments, questions and suggestions

Dear James Colborn (Dr./Prof), we are grateful to the journal **PLOS ONE** for considering our original research manuscript entitled **Susceptibility status of *Aedes aegypti* (Diptera: Culicidae) to public health insecticides in Southern Afar region, Ethiopia** with the manuscript number of PONE-D-24-03915 for possible publication. We also appreciated the academic editor and reviewers to their relevant comments, questions and suggestions. The comments, questions and suggestions forwarded/given are highly helpful and valid towards the improvement of the manuscript. We present point-by-point responses to the editor and reviewers' questions, comments and suggestions below.

Comments on journal requirements

1. Follow the PLOS ONE Journal submission guidelines.

   ***Response*: We have followed PLOS ONE Journal submission guidelines throughout the revised manuscript.**

2. Funding information:

   ***Response*: we have provided the funding information. The statement "the study was financially supported by Addis Ababa University the Vice President for Research and Technology Transfer Office through its thematic research grant" has been included. Unfortunately, we did not include the funding statement in the submission system before. Thus, we kindly request you to incorporate the funding statement on the online submission system.**

3. Non-author contact information:

   ***Response*: To ensure guarantee for the long term stability and availability of the data, we provided the data used as supporting information file 1, supporting information file 2 and supporting information file 3. Supporting information file 1 shows measure ment used to estimate duration in minutes in which KDT_{50} and KDT_{95} *Aedes aegypti* knocked-down and the 95% confidence interval were analyzed (Table 1 in the**

manuscript). Supporting information file 2 presents mortality values of *Ae. aegypti* used to build the percentage mortality graphs (graphs 2-4 in the manuscript). Supporting information file 3 reveals representative GPS points of the study sites recorded during *Ae. aegypti* larvae and pupae collection. We mapped the study areas using Ethio-region GIS shape files to show the location of the study sites. We did not use map image for analysis in the result section of the manuscript.

4. Figure 1 copy-right:

   Response: **Authors have mapped the study area (named as Figure 1 in the manuscript) using arc GIS software version 10.8 from GIS Shape files of Ethiopia freely and no individual or organization involved. Thus, there is no copy-right issue for this figure.**

Additional Editor's comments

All reviewers in felt that the manuscript lacked sufficient detail to evaluate. In particular they requested more detail on the methodology including the sampling methods and statistical analyses performed. They have also listed additional specific changes that should be addressed prior to resubmission.

Response**: We have provided details on each section as per the comments given by the reviewers. We have made slight modification where necessary in the revised manuscript.**

Reviewer #1

Major comments

1. What criteria underlie the selection of insecticides in this study, specifically the inclusion of three pyrethroids and two carbamates? Additionally, are these insecticides currently in use for public health purposes?

   Response: **Viral diseases outbreaks are continuously reported in Ethiopia including the present study areas. Insecticide based viral diseases transmitting mosquito control is a quick responses during outbreaks. Thus, "the insecticides were selected for the study because they have been used for the control of mosquitoes of public health importance in Ethiopia and the study area". "The insecticides are also among the insecticide classes used in the country". The statements have been included in the methods (phenotypic**

susceptibility of *Aedes aegypti*) sub-section of revised manuscript. Yes, most are being used depending on the local transmission situation.

2. According to the WHO guideline cited in the methodology section, the recommended discriminating doses for susceptibility tests of *Aedes* mosquitoes using pyrethroids are 0.25% Permethrin, 0.03% alpha-cypermethrin, and 0.03% deltamethrin. However, it appears that different discriminating doses of pyrethroids were used for the study. Why it is different?

   ***Response*: Thank you for the important question. The insecticides are used for public health in the study areas in particular and in Ethiopia in general with the same concentration of the present study tests. Thus, we used concentrations of the insecticides, WHO criteria, used to evaluate insecticide susceptibility status of *Anopheles arabiensis.* Furthermore, studies from Tanzania and Burkina Faso were used similar concentration of insecticides to test *Aedes aegypti* susceptibility to insecticides** (Kahamba et al., 2020; Namountougou et al., 2020)**.**

3. Why only phenotypic resistance studied? Was there a specific reason for not investigating the resistance mechanisms at play in the mosquito populations?

   ***Responses*: We share the comment as a limitation. We were not able to detect resistant genes to bendiocarb and propoxur due to lack of the reagents. We recommend further molecular investigations regarding the resistant genes.**

4. How did you identified the *Aedes* mosquitoes used for the susceptibility tests were all *Ae. Aegypti*?

   ***Responses*: Experienced personnel were carried out the collection and insecticide susceptibility tests. Selected numbers of the mosquitoes were identified before the test as *Ae. aegypti*. All the mosquitoes were checked for being *Ae. aegypti* at the end of each experiment. All the mosquitoes were identified as *Ae. aegypti*.**

5. How and why was the association between pyrethroid resistance in the study area and agricultural practices established?

   ***Responses*: Pyrethroids are mainly used in the control of mosquitoes that transmit viral diseases and malaria. They are also used in the control of agricultural pests (Shafer et al., 2008; Chrustek et al., 2018). It is known that insecticides used in agricultural practices induce public health insects to develop resistance against public health insecticides. Because some insecticides used in agricultural insects share a similar**

mechanism of action and bio-active ingredients with public health insecticides. In the study areas, farmers used the pyrethroids to control pests of crops. As a result, uses of the insecticides in agriculture might potentially contribute to resistant mosquitoes.

Miner comments

1. Are *Ae. aegypti* and *Ae. bromeliae* the only two species these the only two *Aedes* species existing in the country? For example *Ae. vittaus* and *Ae. africanus* also exist in the country.

   ***Responses*: Thank you for your relevant comment. The statement "In Ethiopia, *Ae. aegypti, Ae. bromeliae, Ae. vittatus, Ae. hirsutus, Ae. simpsoni* complex and *Ae. africanus* are implicated as the potential vectors of viral diseases" has been included in the revised manuscript as suggested.**
2. Why the Knockdown effect of permethrin and alpha-cypermethrin not elaborated like that of deltamethrin?

   ***Responses*: The statement "On the other, *Ae. aegypti* exposed to permethrin showed the lowest KDT_{50} in Awash Arba", has been included in the revised manuscript as commented.**
3. The vertical axis maximum value should not exceed 100 (Fig 2). This works for all graphs.

   ***Responses*: We have corrected the vertical axis of all the graphs to 100 in the revised manuscript as suggested.**
4. The susceptibility test of alpha-cypermethrin in Awash Arba town repeated or not?

   ***Response*: Yes, the test was repeated. The statement "*Ae. aegypti* susceptibility test was repeated for alpha-cypermethrin in Awash Arba town since *Ae. aegypti* populations were appeared to be suspected phenotypic resistance to this insecticide" has been included in the revised manuscript.**
5. Why is integrated vector control strategies recommended without supporting data from the study findings?

   ***Responses*: Thank you for the relevant question. We modified the conclusion based on the findings of the study in the revised manuscript.**
6. How are pyrethroids currently utilized in the control of vectors?

***Response*: Pyrethroids are utilized currently to control mosquito vectors in the form of long lasting insecticides treated nets or in the insecticide residual spray. Moreover, recently the mosquito vector emerge resistance to pyrethroids. Thus, potential new approaches existing pyrethroids are emerging such as pyrethroids plus PBO (pyrethroid synergist piperonyl butoxide) which are used to break the pyrethroid resistance by blocking metabolic resistance. The PBO are not acting as insecticides but they facilitate the action of the pyrethroids.**

Suggestions:

***Response*: All the suggestions given have been included in the revised manuscript.**

Reviewer #2

I suggest improvement in the manuscript and offer the following comments and suggestions:

Overall: correct numerous typos and improve English language; ensure the references cited in the text match with those in the References list.

***Response*: The grammar and typos have been checked and corrected in the revised the manuscript. Similarly, all references cited in the text have been checked their correctness in the reference list.**

Abstract:

- Background: In the last sentence of the "Background", mention names of the insecticides instead of stating "public health insecticides".

***Response*: "deltamethrin, permethrin, alpha-cypermethrin, pirimiphos-methyl, bendiocarb and propoxur" have been included in the revised manuscript in the last sentence of Abstract's background.**

-Methodology: Modify the sentence "Mortality was recorded after 24 hours" to "The mortality in mosquitoes was recorded 24 hours after 60 minutes of exposure". Delete the abbreviation "KDT" for knockdown rate since later you have used KDT for the knockdown time.

***Response*: "The abbreviation "KDT" has been deleted. The mortality in mosquitoes was recorded 24 hours after 60 minutes of exposure" has been added in revised man uscript as suggested.**

Results: In this sentence KDT95 implies Knockdown time, so modify the sentence to

"*Aedes aegypti* from Awash Sebat showed high knock down time (KDT_{95} 34.01 minutes) to deltamethrin …."

***Response*: "Deltamethrin in *Ae. aegypti* showed high knockdown time (KDT_{95} 34.01 minutes) in Awash Sebat as compared to other towns has been rephrased.**

-Results: Rename "alphacypermethrin" to "alpha-cypermethrin", "delthamethrin" to "deltamethrin" and "pirimiphos methyl" to "pirimiphos-methyl" throughout the manuscript (in text, figures, and tables).

***Response*: "alphacypermethrin" to "alpha-cypermethrin", "delthamethrin" to "delt amethrin", "pirimiphosmethyl" to "pirimiphos-methyl" have been renamed in the entire revised manuscript sections as suggested.**

- Conclusion: delete "control" at the end of the second sentence. Mention the insecticide discriminating concentration for each insecticide impregnated paper used. Also state that further studies are required to confirm resistance to alpha-cypermethrin.

***Response*: The word control has been deleted from the conclusion. In addition, the statement "However, further studies should be carried out to evaluate the susceptibility of *Ae. aegypti* to alpha-cypermethrin insecticide in the Awash Arba area", has been included in the revised manuscript.**

-Introduction

-Correct typos: change "*Ae. albopictus*[11, 12]." to "*Ae. albopictus* [11, 12].", "*Ae .aegypti*" to "*Ae. aegypti*", "Fenitrothion" to "fenitrothion".

***Response*: "*Ae. albopictus*[11, 12]," has been corrected to "*Ae. albopictus* [11, 12]." *Ae .aegypti*" to "*Ae. aegypti*", "Fenitrothion" to "fenitrothion", in the revised the manuscript as suggested.**

-Material and Methods

Study sites: Correct many typos (e.g. "820masl" to "820 masl" and so on.

***Response*: "820masl" to "820 masl", "22.6ºC to 30.6ºC" to "22.6 ºC to 30.6 ºC" and "606.6mm" to "606.6 mm" have been corrected in the revised manuscript.**

Exactly what insecticide spraying is practiced in the study areas for the control of malaria and arboviral diseases to give an indication of insecticidal interventions used.

***Response*: The statement "Insecticide such as deltamethrin, permethrin, alpha-cypermethrin, pirimiphos-methyl bendiocarb and propoxur are used," has been**

included in the revised version of the manuscript.

Figure 1 is not mentioned in the text under the study sites.

Response**: Fig 1, has been cited in the text in the revised manuscript.**

Insecticide susceptibility:

- Reference # 16 attributed to the WHO procedure is incorrect. Please recheck correctness of all references used in the text.

Response**: All the references have been checked and reference number 16 has been corrected by reference 20 in the revised manuscript.**

- Correct numerous typos such as "delta-methrin" to "deltamethrin".

Response**: delta-methrin" has been corrected to "deltamethrin in the revised manuscript.**

- A correct number of mosquitoes (100) were exposed in the treatments but not in the control (40 instead of 50). This is a slight deviation from the WHO tube test procedure.

Response**: We followed the WHO 2016 insecticide susceptibility test and exposed the control mosquitoes following the procedures in that manual.**

- "fed on 10% sugar solution" is an incorrect statement; instead, state "were offered cotton wool pads soaked in 10% sugar solution".

Response: **the statement "were offered cotton wool pads soaked in 10% sugar solution" has been corrected in the revised manuscript as suggested**.

Data analysis

Correct this statement "suspected for resistance were with a mortality 90-97%" to "suspected of resistance were with a mortality of 90–97%".

Response: **the statement "suspected of resistance were with a mortality of 90–97%" has been added in the revised manuscript.**

Reference # 34 is incorrect; the correct reference should be: WHO (2022). Manual for monitoring insecticide resistance in mosquito vectors and selecting appropriate interventions. https://iris.who.int/handle/10665/356964.

Response**: Since we initially used WHO (2016). Monitoring and managing insecticide resistance in *Aedes* mosquito populations: interim guidance for entomologists. Thus reference # 34 has been corrected by reference 20.**

-Correct the abbreviations “50% (KD50) and 95% (KD95)” to “50% (KDT50) and 95% (KDT95).

Response: **The abbreviations “50% (KD50) and 95% (KD95)” have been corrected to “50% (KDT_{50}) and 95% (KDT_{95}) in the revised manuscript as suggested.**

-Results

-In Table 1 header, change “KDT50±SE” to “KDT50 ± SE” and “KDT95±SE” to “KDT95 ± SE”

Response: **We have used estimated “KDT50 “KDT95 with their 95% confidence interval in the revised manuscript.**

- Correct numerous typos in the text and figures.

Response**: we have edited typos in the text and corrected the vertical axis of the figures in the revised manuscript.**

-Discussion:

-Is there any publication supporting the following statement “*Ae. aegypti* is generally considered as exophilic, exophagic …....”.

Response: **reference number 37 cited in this study support the high exophilic and exophagic-ness of Ae. *aegypti.***

- While KDT95 for deltamethrin in one town was higher, the tube test does confirm full susceptibility of *Ae. aegypti*.

Response: **The KDT_{95} for deltamethrin in Awash Sebat higher as compared to other pyrethroids in the study. Thus, it did not necessarily confirm resistance or suspect resistance since the mortality of *Ae. aegypti* were determined after 24 hrs.**

- Some of the WHO insecticide discriminating concentrations for *Aedes aegypti* adults have changed in 2022 following the publication of the above said WHO manual based on a WHO multi-centre study in which the authors’ institution was a collaborator, as follows:

o deltamethrin 0.05% to 0.03%

o permethrin 0.75% to 0.40%

o bendiocarb 0.1% to 0.2%

o pirimiphos-methyl 0.25% to 60 mg/m2

o alpha-cypermethrin 0.05% : no change

o propoxur 0.1% : no change

-Since *Ae. aegypti* was found resistant to bendiocarb 0.1% concentration while its concentration has been increased to 0.2%, discuss that further tests should be conducted with 0.2% impregnated papers to confirm resistance.

Response: **the statements "World health organization in 2022 has recommended that the insecticide discriminating dose of bendiocarb for *Ae. aegypti* is changed from its initial 0.1% to 0.2% [45]. Thus, future efficacy studies of bendiocarb should target the 0.2% concentration", have been added in the revised manuscript.**

References

- I did not check correctness of each reference cited, but I noticed at least two incorrect references, therefore authors should recheck all references cited.

Response: **All the references cited in the text have been checked to their correctness in the reference list.**

Reviewer #3:

The authors have a good reason for conducting this research, the manuscript is well organized and written clearly enough to be accessible to non-specialists. However, lacks a detailed description of the method used to collect larvae and pupae.

It is understood that they collected in three places in the South of the Afar region of Ethiopia, where vector-transmitted infections occur, and that they did so from July to October 2022. However, they do not specify how many times each of the locations were visited during that 3 to 4 months for the collections to obtain a variety of samples, nor how many breeding sites they found from where they collected the specimens.

Response**: The statements "A cross-sectional study was designed to collect larvae and pupae of *Aedes* species from July to October, 2022 in the Awash Sebat, Awash Arba and Werer towns. The study towns were selected purposively in collaboration with the local district health professionals and on the basis of the recent outbreak reports of dengue fever and chikungunya virus. The towns were surveyed once per month", have been included in the revised manuscript.**

The latter is important because it is not known if the offspring they collected come from the same progeny or from few of them, hence the importance of collecting in different breeding sites and at different collection times in the same place. The material collected

on the same day at various breeding sites of a locality must be combined so that there is mixing of the genes and thus the results of resistance to insecticides may correspond to several parental mosquitoes originated from the collected site. It is important to describe the number of samples collected on the dates in each of the locations. If possible, describe the number of larvae and pupae collected or an approximate number.

Response: The objective of the study was to collect sufficient number of *Ae. aegypti* larvae and pupae for the susceptibility test. We did not record habitat characteristics, counts of larvae and pupae.

By other hand, it is not specified how many experiments were carried out for the tests with each insecticide. It is understood that only 100 mosquitoes were used and 40 for two control tubes, so that makes only a test. However, at least two other replicates of collections separated in time must be carried out for the same collection location. In this way you can work with an average mortality for more precise data on the locality that is being collected, and if differences in mortalities are found in the three collections could be a good motive for discussion in this manuscript to enrich the discussion.

***Response:* Thank you for your relevant suggestion. The tests were performed in replicates (five treatment and two control replicates) for each insecticide as per the World Health Organization 2016 criteria. We believe that the results obtained are sufficiently enough to support the conclusion drawn in the study.**

-Discussion, 3rd paragraph, at the end: referring to the phrase "the findings observed in the present study were lower than results reported in Sudan, Tanzania and Nigeria" it would be good to add if those from Sudan, Tanzania and Nigeria were reported as resistant or only tolerants to deltamethrin, and maybe is good to mention their KDT50 and 95 values, so we can have an idea for comparison.

***Response:* Comparison of KDT_{50} and KDT_{95} values of deltamethrin to the study carried out in Sudan, Nigeria and Tanzania has been made. The statement "Overall the tube test revealed that *Ae. aegypti* were susceptible to deltamethrin in Sudan, Tanzania but suspected resistance in Nigeria," has been included at the end of discussion section 3rd paragraph in the revised manuscript.**

A sentence could be added in this section about the resistance mechanisms that could be present in the mosquitoes detected resistant to propoxur and bendiocarb, but not to

organophosphate. It is known that the two groups of insecticides share the same site of action, and that if resistance to both groups has been diagnosed, such a situation could explain a resistance based on a point mutation in the site of action, however this is not the case.

Response: **The statement "In addition, *Ae. aegpti* resistance to propoxur and bendiocarb may occur as a result of mutation in the insecticide target site enzyme Acetyl-cholinesterase (AChE) as reported in Cuba," has been included in the revised manuscript.**

Minor corrections:

Introduction, 3rd paragraph. "Fenitrothion" change to "fenitrothion"

Response: "Fenitrothion" has been changed to "fenitrothion" in the revised manusc ript.

Material and Methods: 1st paragraph. "Were" change to "Werer"

Response: "Were" has been corrected to "Werer" in the revised manuscript.

Insecticide susceptibility paragraph: "delta-methrin"change to "deltamethrin" and "bendio carb" change to bendiocarb"

Response: delta-methrin" has been changed to "deltamethrin" and "bendio carb" has been changed to bendiocarb" in the revised manuscript.

-Results, 1st paragraph, Table 1. "*Ae. Aegypti*" change to "*Ae. aegypti*"

***Response*:"*Ae. Aegypti*" has been changed to "*Ae. aegypti*" in the revised manuscript**

Discussion, 4th paragraph. "propxur" change to "propoxur"

***Response*: propxur" has been change to "propoxur" in the revised manuscript**

Conclusion. "perimethrin" change to "permethrin"

***Response*: "perimethrin" has been changed to "permethrin" in the revised manuscript.**

Check the double spaces between words throughout the writing and correct them to one space.

***Response*: We have made the manuscript text double space while one space between words in the revised manuscript.**

Reference

Kahamba NF, et al. Habitat characteristics and insecticide susceptibility of *Aedes aegypti* in the Ifakara area, south-eastern Tanzania. Parasit Vectors. 2020; 13(1) 1-15.

Namountougou M, et al. Monitoring Insecticide Susceptibility in Aedes Aegypti Populations from the Two Biggest Cities, Ouagadougou and Bobo-Dioulasso, in Burkina Faso: Implication of Metabolic Resistance. Trop. Med. Infect. Dis. 2020; 5(84):1-9.

Shafer TJ, Rijal SO, Gross GW. Complete inhibition of spontaneous activity in neuronal networks in vitro by deltamethrin and permethrin. *Neuro Toxicology*. 2008;29:20-212.

Chrustek A, Hołyńska-Iwan I, Dziembowska I, Bogusiewicz J, Wróblewski M, Cwynar A. et al. Current Research on the Safety of Pyrethroids Used as Insecticides. 2018; 54(4): 61
