# Supplementary material for: Susceptibility status of Aedes aegypti (Diptera: Culicidae) to public health insecticides in Southern Afar Region, Ethiopia
Source: PLoS One. 2024 Aug 23;19(8):e0309335. doi: 10.1371/journal.pone.0309335 (PMC11343450; doi:10.1371/journal.pone.0309335)
Supplement: Attachment — Submitted filename: a. PONE-D-24-03915_R2_reviewer_comments.pdf [file pone.0309335.s010.pdf]

PLOS ONE

# Susceptibility status of Aedes aegypti (Diptera: Culicidae) to public health insecticides in Southern Afar region, Ethiopia

--Manuscript Draft--

| | |
|---|---|
| **Manuscript Number:** | PONE-D-24-03915R2 |
| **Article Type:** | Research Article |
| **Full Title:** | Susceptibility status of Aedes aegypti (Diptera: Culicidae) to public health insecticides in Southern Afar region, Ethiopia |
| **Short Title:** | Susceptibility status of Aedes aegypti to public health insecticides |
| **Corresponding Author:** | Mohammed Seid<br>Addis Ababa University<br>Addis Ababa, ETHIOPIA |
| **Keywords:** | Aedes aegypti; Afar; Ethiopia; insecticides; Susceptibility |
| **Abstract:** | Mosquito-borne viral diseases such as dengue fever, chikungunya and yellow fever have been documented in Ethiopia since the 1960s. However, efficacy of public health insecticides against Aedes aegypti that transmit these viruses remain poorly understood in the country<br>particularly in the Afar region. Thus, the aim of this study was to assess the susceptibili ty status of Ae. aegypti to deltamethrin, permethrin, alpha-cypermethrin, pirimiphos-me thyl, bendiocarb and propoxur insecticides. Larvae and pupae of Aedes mosquitoes were collected from the Awash Arba, Awash Sebat and Werer towns of the Afar Regional State of Ethiopia during July to October 2022, brought to Aklilu Lemma Institute of Pathobiology, insectary and reared to adults. Non-blood-fed, 3 to 5 days old female Ae. aegypti were exposed to pyrethroid, carbamate and organophosphate insecticides following the World Health Organization standard guidelines. Knockdown rates were noted at 10 min interval until one<br>hour. The mortality in mosquitoes was recorded 24 hours after 60 minutes of exposure. Mortalities of Ae. aegypti exposed to propoxur were 87% in all the study towns. Similar ly, 88% of Ae. aegypti mortality was recorded when treated with bendiocarb in Awash Sebat and Awash Arba towns. Suspected resistance of Ae. aegypti (95% mortality) exp osed to alpha-cypermethrin was recorded from Awash Arba town. However, Ae. aegypti collected from all the three sites was observed to be susceptible to deltamethrin, permethrin, and pirimiphos-methyl. Aedes aegypti was resistant to 0.1% bendiocarb and 0.1% propoxur, and possibly resistant to 0.05% alpha-cypermethrin. On the other hand, it was susceptible to 0.05% deltamethrin,<br>0.75% permethrin and 0.25% pirimiphos-methyl. Thus, deltamethrin, permethrin and pi rimiphos-methyl can be used in the control of adult Ae. aegypti in the Afar Region of Ethiopia. However, further studies should be carried out to evaluate the susceptibility of Ae. aegypti to alpha-cypermethrin insecticide in the Awash Arba area. |
| **Order of Authors:** | Mohammed Seid |
| | Esayas Aklilu |
| | Abebe Animut |
| **Response to Reviewers:** | Comments on journal requirements<br>1.Please review your reference list to ensure that it is complete and correct. If you have cited papers that have been retracted, please include the rationale for doing so in the manuscript text, or remove these references and replace them with relevant current references. Any changes to the reference list should be mentioned in the rebuttal letter that accompanies your revised manuscript. If you need to cite a retracted article, indicate the article's retracted status in the References list and also include a citation and full reference for the retraction notice.<br>Response: Dear Editor, thank you very much once again for your relevant suggestion. We have reviewed the references cited in the text to their correctness in the references list. We have also corrected a few typos of existed references particularly the volume and issue numbers in the revised manuscript. Retracted references have not been used. We have cited the WHO, 2022 of reference number[45] on future |

Powered by Editorial Manager® and ProduXion Manager® from Aries Systems Corporation

recommendation for efficacy studies of bendiocarb that should target to the 0.2% concentration.

2. While revising your submission, please upload your figure files to the Preflight Analysis and Conversion Engine (PACE) digital diagnostic tool.
Response: We have uploaded the figures to PACE to ensure that the figures meet PLOS requirements.
Reviewer #1
The authors have responded to numerous comments, but there is still a gap in explaining why the recommended discriminating doses for susceptibility tests of Aedes mosquitoes, as outlined in the WHO guideline cited in the methodology section, were not followed. This discrepancy must be addressed transparently in the methodology, providing genuine explanations for the deviation. The authors may suggest that the failure to adhere strictly to the WHO-recommended discriminating doses might be due to logistical constraints, such as the unavailability of insecticide-impregnated papers at the time of the trial. This practical impediment necessitated a pragmatic approach where alternative doses were explored. Therefore, the methodology section should clearly state the reasons for the deviation from the recommended doses.
Response: Dear, Reviewer, thank you very much again for your relevant suggestion. The statements “However, we did not follow the World Health Organization recommended discriminating doses for Aedes species due to unavailability of insecticide-impregnated papers at the time of the experiment. This practical obstacle required a strategy that looked into the alternative doses for the insecticides” have been added in the revised manuscript on page 6, Line 14-18.
Reviewer #2
I still noticed many grammatical errors and some typos, and have mentioned them in red fonts at some places in the PDF file to draw attention of the authors to carefully check the text.
1.P1, L18 Typos correction to Non-blood fed
Response: Dear Reviewer, thank you very much again for your important indication on grammatical and typing errors. Thus, “Non-blood fed” has been changed to “Non-blood-fed” in the revised manuscript.
2.P1, L19 “pyrethroids”, “carbamates”
Response: “pyrethroids”, “carbamates” have been corrected to “pyrethroid” and carbamate in the revised manuscript.
3.P1, L25 “were” and L26, “were”
Response: were, were, have been corrected to “was”, “was” in the revised manuscript, because the statement explained about a single species i.e. Ae. aegypti.
4.Residual P3, L19-20
Response: “residual” has been corrected to “residual insecticide surface” in the revised manuscript.
5.P 4, L2, “base line”
Response: “base line” has been corrected to “baseline” in the revised manuscript.
6.P7, delete the statement “The knockdown effect of pyrethroids against the Ae. aegypti were recorded during the 60 minutes exposure at an interval of 10 minutes from the start.”
Response: “The knockdown effect of pyrethroids against the Ae. aegypti were recorded during the 60 minutes exposure at an interval of 10 minutes from the start” has been removed because of redundancy with the phenotypic susceptibility test section.
7.P8, L3, KDT50 95% [CI] and KTD95 95%, include the 95% inside the close bracket
Response: KDT50 95% [CI] and KDT95 95% [CI] have been corrected to KDT50 [95%CI] and KDT95 [95%CI] in the revised manuscript.
8.P8, L8, “Correct (figs 2-4) to Fig.”
Response: (figs 2-4) has been corrected to (Figs 2-4). Only the first letter should be capitalized and no dot was needed as PLOS ONE Journal guideline for in text citation of multiple figures.
9.P9, L4, “resistance”
Response: “resistance” has been corrected to “to be resistant” in the revised manuscript.
10.P11, L7, “World health organization”
Response: “World health organization” has been corrected to “World Health Organization” in the revised manuscript.
11.P11, L10, “Add reference”:

Powered by Editorial Manager® and ProduXion Manager® from Aries Systems Corporation

| | |
|---|---|
| | Response: The reference WHO, 2022 has been added in the revised manuscript.<br>12.P11, L11, “Mortally”<br>Response: “mortally” has been corrected to “mortality” in the revised manuscript.<br>13.P Add “%” to the insecticides in all figures 2-4<br>Response: “%” has been added to the insecticides in all figures of 2-4 in the revised manuscript. |
| Additional Information: | |
| Question | Response |
| **Financial Disclosure**<br><br>Enter a financial disclosure statement that describes the sources of funding for the work included in this submission. Review the submission guidelines for detailed requirements. View published research articles from *PLOS ONE* for specific examples.<br><br>This statement is required for submission and **will appear in the published article** if the submission is accepted. Please make sure it is accurate.<br><br>**Funded studies**<br>Enter a statement with the following details:<br>• Initials of the authors who received each award<br>• Grant numbers awarded to each author<br>• The full name of each funder<br>• URL of each funder website<br>• Did the sponsors or funders play any role in the study design, data collection and analysis, decision to publish, or preparation of the manuscript?<br><br>Did you receive funding for this work? | Yes |
| Please add funding details.<br>as follow-up to "**Financial Disclosure**<br><br>Enter a financial disclosure statement that describes the sources of funding for the work included in this submission. Review the submission guidelines for detailed requirements. View published research articles from *PLOS ONE* for specific examples.<br><br>This statement is required for submission | The study was financially supported by Addis Ababa University the Vice President for Research and Technology Transfer Office through its thematic research grant. |

| | |
|---|---|
| and **will appear in the published article** if the submission is accepted. Please make sure it is accurate.<br><br>**Funded studies**<br>Enter a statement with the following details:<br>• Initials of the authors who received each award<br>• Grant numbers awarded to each author<br>• The full name of each funder<br>• URL of each funder website<br>• Did the sponsors or funders play any role in the study design, data collection and analysis, decision to publish, or preparation of the manuscript?<br><br>Did you receive funding for this work?" | |
| Please select the country of your main research funder (please select carefully as in some cases this is used in fee calculation).<br>as follow-up to "**Financial Disclosure**<br><br>Enter a financial disclosure statement that describes the sources of funding for the work included in this submission. Review the submission guidelines for detailed requirements. View published research articles from *PLOS ONE* for specific examples.<br><br>This statement is required for submission and **will appear in the published article** if the submission is accepted. Please make sure it is accurate.<br><br>**Funded studies**<br>Enter a statement with the following details:<br>• Initials of the authors who received each award<br>• Grant numbers awarded to each author<br>• The full name of each funder<br>• URL of each funder website<br>• Did the sponsors or funders play any role in the study design, data collection and analysis, decision to publish, or preparation of the manuscript? | |

| | |
|---|---|
| Did you receive funding for this work?" | |
| **Competing Interests**<br><br>Use the instructions below to enter a competing interest statement for this submission. On behalf of all authors, disclose any competing interests that could be perceived to bias this work—acknowledging all financial support and any other relevant financial or non-financial competing interests.<br><br>This statement is required for submission and **will appear in the published article** if the submission is accepted. Please make sure it is accurate and that any funding sources listed in your Funding Information later in the submission form are also declared in your Financial Disclosure statement.<br><br>View published research articles from *PLOS ONE* for specific examples.<br><br>**NO authors have competing interests**<br><br>Enter: *The authors have declared that no competing interests exist.*<br><br>**Authors with competing interests**<br><br>Enter competing interest details beginning with this statement:<br><br>*I have read the journal's policy and the authors of this manuscript have the following competing interests: [insert competing interests here]*<br><br>* typeset | The authors declared that there is no conflict of interest. |
| **Ethics Statement**<br><br>Enter an ethics statement for this submission. This statement is required if the study involved: | "N/A" |

- Human participants
- Human specimens or tissue
- Vertebrate animals or cephalopods
- Vertebrate embryos or tissues
- Field research

Write "N/A" if the submission does not require an ethics statement.

General guidance is provided below. Consult the submission guidelines for detailed instructions. **Make sure that all information entered here is included in the Methods section of the manuscript.**

*Powered by Editorial Manager® and ProduXion Manager® from Aries Systems Corporation*

| | |
|---|---|
| **Format for specific study types**<br><br>**Human Subject Research (involving human participants and/or tissue)**<br>• Give the name of the institutional review board or ethics committee that approved the study<br>• Include the approval number and/or a statement indicating approval of this research<br>• Indicate the form of consent obtained (written/oral) or the reason that consent was not obtained (e.g. the data were analyzed anonymously)<br><br>**Animal Research (involving vertebrate animals, embryos or tissues)**<br>• Provide the name of the Institutional Animal Care and Use Committee (IACUC) or other relevant ethics board that reviewed the study protocol, and indicate whether they approved this research or granted a formal waiver of ethical approval<br>• Include an approval number if one was obtained<br>• If the study involved *non-human primates*, add *additional details* about animal welfare and steps taken to ameliorate suffering<br>• If anesthesia, euthanasia, or any kind of animal sacrifice is part of the study, include briefly which substances and/or methods were applied<br><br>**Field Research**<br><br>Include the following details if this study involves the collection of plant, animal, or other materials from a natural setting:<br>• Field permit number<br>• Name of the institution or relevant body that granted permission | |
| **Data Availability**<br><br>Authors are required to make all data underlying the findings described fully available, without restriction, and from the time of publication. PLOS allows rare exceptions to address legal and ethical concerns. See the PLOS Data Policy and FAQ for detailed information. | Yes - all data are fully available without restriction |

| | |
|---|---|
| A Data Availability Statement describing where the data can be found is required at submission. Your answers to this question constitute the Data Availability Statement and **will be published in the article**, if accepted.<br><br>**Important:** Stating 'data available on request from the author' is not sufficient. If your data are only available upon request, select 'No' for the first question and explain your exceptional situation in the text box.<br><br>Do the authors confirm that all data underlying the findings described in their manuscript are fully available without restriction? | |
| **Describe where the data may be found in full sentences. If you are copying our sample text, replace any instances of XXX with the appropriate details.**<br><br>• If the data are **held or will be held in a public repository**, include URLs, accession numbers or DOIs. If this information will only be available after acceptance, indicate this by ticking the box below. For example: *All XXX files are available from the XXX database (accession number(s) XXX, XXX.)*.<br>• If the data are all contained **within the manuscript and/or Supporting Information files**, enter the following: *All relevant data are within the manuscript and its Supporting Information files.*<br>• If neither of these applies but you are able to provide **details of access elsewhere**, with or without limitations, please do so. For example:<br><br>*Data cannot be shared publicly because of [XXX]. Data are available from the XXX Institutional Data Access / Ethics Committee (contact via XXX) for researchers who meet the criteria for access to confidential data.*<br><br>*The data underlying the results presented in the study are available from (include the name of the third party* | All relevant data are within the manuscript and its Supporting Information files. |

| | |
|---|---|
| *and contact information or URL).*<br>• This text is appropriate if the data are owned by a third party and authors do not have permission to share the data.<br><br>* typeset | |
| Additional data availability information: | |

Powered by Editorial Manager® and ProduXion Manager® from Aries Systems Corporation

Manuscript
Click here to access/download;Manuscript;Manuscript.doc

# Susceptibility status of *Aedes aegypti* (Diptera: Culicidae) to public health insecticides in Southern Afar region, Ethiopia

**Mohammed Seid[1, 2*], Esayas Aklilu[1], Abebe Animut[1]**

[1] Aklilu Lemma Institute of Pathobiology, Addis Ababa University, Addis Ababa, Ethiopia

[2] Department of Biology, College of Natural and Computational Sciences, Mattu University, Mattu, Ethiopia

*Corresponding author

Email: mohammed.seid.legas@gmail.com (MS)

# Abstract

Mosquito-borne viral diseases such as dengue fever, chikungunya and yellow fever have been documented in Ethiopia since the 1960s. However, efficacy of public health insecticides against *Aedes aegypti* that transmit these viruses remain poorly understood in the country particularly in the Afar region. Thus, the aim of this study was to assess the susceptibility ~~stat~~ status ~~us~~ of *Ae. aegypti* to deltamethrin, permethrin, alpha-cypermethrin, pirimiphos-methyl, bendi ocarb and propoxur insecticides. Larvae and pupae of *Aedes* mosquitoes were collected from the Awash Arba, Awash Sebat and Werer towns of the Afar Regional State of Ethiopia during July to October 2022, brought to Aklilu Lemma Institute of Pathobiology, insectary and reared to adults. Non-blood-fed, 3 to 5 days old female *Ae. aegypti* were exposed to pyrethroid, carbamate and organophosphate insecticide~~s~~/ impregnated papers in tube tests following the World Health Organization standard guidelines. Knockdown rates were noted at 10 min interval until one hour. The mortality in mosquitoes was recorded 24 hours after 60 minutes of exposure. Morta lities of *Ae. aegypti* exposed to propoxur were 87% in all the study towns. Similarly, 88% ~~of~~ mortality in *Ae. aegypti* ~~mortality~~ was recorded when ~~treated~~ tested with bendiocarb in Awash Sebat and Awash Arba towns. Suspected resistance of *Ae. aegypti* (95% mortality) ~~exposed~~ to alpha-cypermeth rin was recorded from Awash Arba town. However, *Ae. aegypti* collected from all the three sites was observed to be susceptible to deltamethrin, permethrin, and pirimiphos-methyl.

*Aedes aegypti* was resistant to 0.1% bendiocarb and 0.1% propoxur, and possibly resistant to 0.05% alpha-cypermethrin. On the other hand, it was susceptible to 0.05% deltamethrin, 0.75% permethrin and 0.25% pirimiphos-methyl. Thus, deltamethrin, permethrin and pirimiphos-methyl can be used in the control of adult *Ae. aegypti* in the Afar Region of Ethiopia. However, further studies should be carried out to evaluate the susceptibility of *Ae. aegypti* to alpha-cypermethrin insecticide in the Awash Arba area.

---

# Introduction

The ever increasing outbreaks of arboviral diseases drew public attention in several countries of the world [1]. The East African countries including Kenya, Somalia, Djibouti, Eritrea and Ethiopia are epicentres of outbreaks of arboviral diseases such as dengue fever, yellow fever and chikungunya virus [2-4]. In Ethiopia, yellow fever caused about 30,000 deaths from over 200,000 infections during the 1960 and 1962 [5]. Recently, there have been yellow fever outbreaks in South Omo [6], dengue fever outbreaks in Dire Dawa city administration, Somali Regional State, Gewane and Amibara districts of Afar Regional State [7, 8]. In addition, chikungunya outbreaks were also reported in Dire Dawa city administration, Afar and Somali Regional States [9, 10]. All these outbreaks with potentially unrecognized and/or under reported deaths owing to the highly limited laboratory facilities in the country.

Mosquitoes transmit over 90% of the viral pathogens through their bites among which *Ae. aegypti* is the leading vector followed by *Ae. albopictus* [11, 12]. *Ae. aegypti* is anthropophilic in its behaviour and occurs in close proximity to human habitations [13, 14]. *Ae. aegypti* has two morphological subspecies (ecotypes) the domestic *Ae. aegypti aegypti* and sylvan *Ae. aegypti formosus* [15, 16]. In Ethiopia, *Ae. aegypti*, *Ae. bromeliae*, *Ae. vittatus*, *Ae. hirsutus*, *Ae. simpsoni* complex and *Ae. africanus* are implicated as the potential vectors of viral diseases [6, 8, 17-19].

In the absence of effective therapeutic drugs and vaccines to treat and control mosquito borne viral diseases, prevention~~s~~ largely depend on vector control ~~tools~~ with mainly public health insecticides [13]. The pyrethroids such as deltamethrin, alpha-cypermethrin, permethrin and organophosphates pirimiphos-methyl and fenitrothion are among the public health insecticides used against *Aedes* mosquitoes [20]. However, the potential resistance to the insecticides may adversely affect implementation of the control strategy in endemic areas [21]. Mosquitoes could resist insecticides through their inherent ability to detoxify and avoid contacts either behaviourally or physiologically [22].

Evidences from many African countries indicate that the *Anopheles* and *Aedes* mosquitoes developed resistance to insecticides [23-25]. For instance in Ethiopia, malaria transmitting *Anopheles arabiensis* has developed resistance to insecticides in several areas of the country including in the Amibara district of the Afar Regional State [25, 26]. The causes for the resistance could be frequent use of the ~~insecticide chemicals~~ insecticides in indoor residual spraying and long-lasting insecticidal ~~treated~~ nets for the control of mosquito borne diseases in the area [21] or in ~~argro-pesticides~~ agriculture pest control [27]. However, the susceptibility status of *Aedes* mosquitoes to insecticides currently used is lacking in the country.

Recently, the Ethiopian Public Health Institute prepared arboviral disease vectors, surveillance and control guideline in 2021 ~~through~~ emphasizing the use of chemical and environmental management methods. Vector control by chemical method such as fumigation, repellents and residual insecticides surface treatments were implemented in Dire Dawa city administration and Somalia Regional State during dengue fever and chikungunya outbreaks (EPHI, 2021 unpublished). Unfortunately, there is a scarcity of evidences on the efficacy of the insecticides for the control of virus transmitting *Aedes* species in the Southern Afar Region in the presence of increased reports of viral diseases incidence and prevalence. Therefore, assessing the effectiveness of the existing public health insecticides to control *Aedes* species

is crucial to strengthen the arboviral diseases prevention and control programs in the country. This inspired us to generate baseline data ~~in relation to the applicability of insecticide control tools in the prevention and control of virus transmitting *Aedes* mosquitoes in the area. Therefore, the aim of the study was to assess~~ on the susceptibility status of *Ae. aegypti* to public health insecticides in the Southern part of the Afar Regional State of Ethiopia.

# Materials and Methods

### Study sites

The study was carried out in Awash Arba, Werer and Awash Sebat towns, Gabi-Rasu Zone (Zone 3), of Afar Regional State, Ethiopia. The Awash Arba and Werer towns are located about 250 km to the North-east of Addis Ababa at 9.33453°N latitude, 40.181385°E longitude and an altitude range of 720 metres above sea level (masl) to 1100 masl (Central statistics Agency, 2019 unpublished). Awash Arba and Werer sites were selected from Amibara district which are about 30 km distance far apart (Fig 1). Agro-ecologically, these two towns are semi-arid with temperature ranging from 25 °C to 35 °C, and average annual rainfall of 530 mm. The communities ~~livelihoods are characterized as~~ are pastoralist and agro-pastoralist in which they mainly obtained income ~~on livestock and from livestock~~ from livestock rearing and other agricultural crop production [28].

Awash Sebat town administration is located 214 km North-east of Addis Ababa at 8.98810°N latitude and 40.163936°E longitude (Central statistics Agency, 2019 unpublished). Its altitude ranges from 820 masl to 1120 masl and the temperature varies from 22.6 °C to 30.6 °C. The town and its surroundings receive a mean annual rainfall of 606.6 mm.

The study areas are found in the middle Awash Valley, where infectious disease such as malaria, chikungunya and dengue fever are common [29]. Considerable attention has been given by the regional government to achieve better health in the region including the three towns [30]. The region has hospitals, health centres, clinics and health posts in different

zones and districts. There are one hospital, one clinic and five health centres and few private clinics. Insecticide such as deltamethrin, permethrin, alpha-cypermethrin, pirimiphos-methyl bendiocarb and propoxur are used ~~as insecticide residual spraying and/or long lasting~~ for indoor residual spraying, or pyrethroids in long-lasting insecticide ~~treated~~ nets for the control of malaria and arboviral diseases in these towns [26, 31]. Similarly, agro-chemicals and agro-pesticides are used since maize and sorghum productions are common in these areas which is used to improve agricultural crop production [32].

**Fig 1. Map of the study areas.** A map showing the locations of the three study areas**:** Awash Sebat, Awash Arba and Werer Towns, in the Southern Afar Region, Ethiopia.

### Larval collection and rearing

A cross-sectional study was designed to collect larvae and pupae of *Aedes* species from July to October, 2022 in the Awash Sebat, Awash Arba and Werer towns. The study towns were selected purposively in collaboration with the local district health professionals and on the basis of the recent outbreak reports of dengue fever and chikungunya virus (Awash Sebat town and Amibara district health bureaus, 2022 unpublished). The towns were surveyed once per month. Larvae and pupae were collected from different positive habitats including tyres, water storage drums, discarded plastics and metallic containers using standard dipper (350 ml), ladles and ~~pipets/nets. The collected larvae and pupae,~~ pippetes and nets. The larvae and pupae collected from each habitat, were transferred into with labelled plastic jars, transported to the insectary of Aklilu Lemma Institute of Pathobiology, Addis Ababa University, Ethiopia and reared to adults under standard laboratory conditions. Tetra cichlid floating fish food (®/TM/©2019 Germany) was provided as food to the larvae. The emergence of larvae into in to pupae was checked each morning and pupae were collected in glass flasks and transferred in to (30cm x 30cm x 30cm) netted cages. Emerged adult *Ae. aegypti* were kept at 27 ± 2 °C temperature, 75 ± 10% relative

humidity and provided acccess to feed ~~fed~~ on 10% sucrose solution until the bioassay tests. Emergent adults were identified to species using morphological taxonomic keys for *Aedes* species [33].

**Phenotypic susceptibility status of *Aedes aegypti***

Prior to insecticide susceptibility tests, sugar fed female *Ae. aegypti* were starved for 2hrs ~~in order to prevent them from having high weight which leads them to be knocked-down~~. Non-blood-fed, 3-5 days old female *Ae. aegypti* were tested for their susceptibility to insecticides in tube tests following the World Health Organization insecticide testing procedures [20]. They were exposed to pyrethroids (0.05% deltamethrin, 0.05% alpha-cypermethrin, 0.75% permethrin), carbamates (0.1% bendiocarb and 0.1% propoxur) and organophosphate (0.25% pirimiphos-methyl) which are commonly used for preventing disease transmitting mosquitoes [20]. The insecticides were selected for the study because they have been used for the control of public health important mosquitoes in Ethiopia and the study area. The insecticides are also among the insecticide classes used in the country. Similar concentrations of insecticides were used to test the susceptibility of *Ae. aegypti* in Tanzania [34]. However, we did not follow the World Health Organization recommended discriminating concentrations ~~doses~~ for *Aedes* species due to unavailability of insecticide-impregnated papers at the time of the experiment. This practical obstacle required a strategy that looked into the alternative doses for the insecticides. In each test, 20 female *Ae. aegypti* were exposed to each five treatment replicates and two control test tubes making a total of 140 mosquitoes in a single test. The exposed mosquitoes were observed for 60 minutes at an interval of 10 minutes to observe their knockdown. After an hour, the exposed females were transferred into holding tubes and kept for 24 hrs at 27.0 ± 2.0 °C and 75 ± 10% relative humidity and were offered cotton wool pads soaked in 10% sugar solution. The mortality in mosquitoes was recorded 24 hrs after 60 minutes of exposure. *Ae. aegypti* susceptibility test was repeated for alpha-cypermethrin in Awash Arba town since *Ae. aegypti* populations were appeared to be suspect phenotypic resistance to this

insecticide. However, in case of Werer town the susceptibility status of *Ae. aegypti* to bendiocarb was not conducted due to insufficient mosquito samples.

**Ethical Issues**

The study protocol We obtained ethical approval from the institutional review board (IRB) of Aklilu Lemma Institute of Pathobiology, Addis Ababa University, Ethiopia (June 13, 2022; ALIPB IRB/80/2022). Written permission letters were obtained from the district health bureau and health centres of the towns. The households were informed about the objective of the study and their agreements were sought prior to the larvae/pupae collection.

**Data analysis**

Mortality was determined on the basis of the dead and alive mosquitoes after the 24 hours recovery period. Susceptible were those with a mortality in the range of 98-100%, suspected for resistance were with a mortality 90-97% and resistant if mortality was <90% [20]. Probit analysis was used to determine estimated duration at which 50% ($KDT_{50}$) and 95% ($KDT_{95}$) of the exposed *Ae. aegypti* mosquitoes were knocked-down. Overall adult mortalities and knockdown times were computed using SPSS Software version 20.

# Results

**Knockdown effect of insecticides against *Aedes aegypti***

Knockdown time of *Ae. aegypti* exposed to insecticides is given was depicted in Table 1. The time to knockdown 50% knockdown effect of 50% ($KDT_{50}$) of *Ae. aegypti* exposed to deltamethrin ranged from 12.15 to 17.06 minutes whereas $KDT_{95}$ of the insecticide ranged from 18.74 to 34.01 minutes. Deltamethrin in *Ae. aegypti* showed high The knockdown time ($KDT_{95}$, 34.01 minutes) for Ae. aegypti collected from Awash Sebat was found to be longer than for in Awash Sebat as compared to other towns (Table 1). On the other hand, *Ae. aegypti* exposed to permethrin showed the lowest $KDT_{50}$ in Awash Arba town ($KDT_{50}$, 5.11). Overall, the $KDT_{50}$ and $KDT_{95}$ of *Ae. aegypti* varied among *Ae. aegypti* in knockdown to insecticides across the study localities.

**Table 1** The time (minutes) to knockdown ~~Knockdown times (in minute)~~ **of 50% and 95% of *Ae. aegypti* exposed to commonly used** pyrethroids at ~~insecticides in~~ **three localities** ~~from July to October 2022~~ **in Southern Afar region Ethiopia** during July-October 2022

| Insecticides | Localities | $KDT_{50}$ [95%CI] | $KDT_{95}$ [95%CI] |
|---|---|---|---|
| Deltamethrin | Awash sebat | 17.06 [15.81-18.28] | 34.01 [31.02-38.12] |
| | Awash Arba | 12.15 [11.41-12.97] | 18.74 [16.98-21.56] |
| | Werer | 16.53 [15.37-17.67] | 30.92 [28.24-34.62] |
| Permethrin | Awash Sebat | 8.92 [7.34-10.26] | 24.21 [21.23-28.93] |
| | Awash Arba | 5.11 [2.91-6.94] | 19.23 [16.23-24.09] |
| | Werer | 15.12 [12.45-17.66] | 29.89 [24.64-41.53] |
| Alpha-cypermethrin | Awash Sebat | 14.35 [13.35-15.35] | 24.93 [22.77-27.97] |
| | Awash Arba | 13.47 [12.64-14.36] | 20.78 [19.04-23.32] |
| | Werer | 16.71 [13.85-19.46] | 32.90 [27.26-44.98] |

CI, Confidence interval; $KDT_{50}$, time taken for 50% of the tested Ae. *aegypti* to be knocked-down; $KDT_{95}$ time taken for 95% of the tested *Ae. aegypti* to be knocked-down

**Susceptibility status of *Aedes aegypti* to insecticides**

The percentage mortalities of *Ae. aegypti* mosquito populations against insecticides ~~were~~ are presented in ~~figures (~~Figs 2-4). Accordingly, *Ae. aegypti* mosquitoes showed phenotypic resistance to bendiocarb and propoxur insecticides with mortality of ~~in which the percentage mortalities were recorded to be~~ 88% and 87% respectively in Awash Sebat town. There was 100% mortality of Ae. aegypti with ~~*Ae. aegypti* mortalities to~~ deltamethrin, permethrin alpha-cypermethrin ~~alph-acypermthrin~~ and pirimiphos-methyl ~~were 100%~~ in Awash Sebat town (Fig 2). All mosquitoes in the control group showed 0% mortality in all study sites.

**Fig 2. Insecticide susceptibility status of *Ae. aegypti* populations from Awash Sebat town, Southern Afar region, Ethiopia, July to October 2022.** Error bars represent 95% confidence intervals.

~~In addition,~~ *Ae. aegypti* ~~mosquitoes~~ from Awash Arba area were also observed to be resistant to bendiocarb and propoxur ~~from Awash Arba. Moreover, *Ae. aegypti* mosquitoes were observed to be suspecte~~ but were possibly resistant d resistant to alpha-cypermethrin ~~in which the mortality of *Ae. aegypti* mosquitoes due to alpha-cypermethrin was 95%~~ (mortality 95%) (Fig 3). *Ae. eagypti* ~~mortalities were observed to be 100% when exposed~~ was fully susceptible to deltamethrin, permethrin and pirimiphos-methyl insecticides in this town.

**Fig 3. Insecticide susceptibility status of *Ae. aegypti* populations from Awash Arba town, Southern Afar region, Ethiopia, July to October 2022.** Error bars represent 95% confidence intervals.

Mortality in Ae. aegypti from Werer town was 87% with propoxur and 99% with alpha-cypermethrin ~~*Ae. aegypti* mortalities when exposed to propoxur insecticide were 87% whereas 99% mortalities were observed among the *Ae. aegypti* mosquitoes from Werer town when treated with alpha-cypermethrin insecticide~~ (Fig 4). However, ~~100% mortalities of~~ *Ae. aegypti* from this site ~~were recorded~~ was found to be fully susceptible to deltamethrin, permethrin and pirimiphos-methyl.

**Fig 4. Insecticide susceptibility status of *Ae. aegypti* populations in Werer town, Southern Afar region, Ethiopia, July to October 2022.** Error bars represent 95% confidence intervals.

# Discussion

Recent outbreaks of dengue fever and chikungunya virus fever in the Eastern and the North-eastern parts of Ethiopia especially in Dire Dawa city administration, Somali Regional State and Amibara and Gewane districts of Afar regional State requires the need for the control of the *Aedes* species that transmit the viruses. The insecticide based mosquito control is underway in Ethiopia in general and in Afar regional State in particular [7, 35]. In so doing, there have been continuous evaluation of efficacy of the insecticides against malaria transmitting *Anopheles* species but there is scarcity of research findings conducted to control *Aedes* species either chemically, biologically or other means of vector control strategies in the

country [36]. Thus, the control of viral disease transmitting *Aedes aegypti* vector is poorly investigated and needs detailed research. As far as our knowledge is concerned, this study was first in its kind to report the susceptibility status of *Ae. aegypti* to public health insecticides in the Southern Afar Regional State and there are limited data to compare our findings to others in the country.

*Aedes aegypti* is generally ~~considered as exophillic, exophagic~~ endophilic and endophagic? and day time biters. They usually feed on human and other vertebrate hosts as described in previous studies [37, 38]. However, they may tend to be endophilic and endophagic in which they rest and fed indoors [39]. This makes ~~controlling of the~~ control of *Ae. aegypti* and the ~~viral diseases~~ Aedes-borne diseases they transmit more difficult [40]. As a result, different intervention tools have been adopted to control these mosquitoes including indoor residual insecticide spraying and long lasting insecticide treated nets in addition to managing their breeding habitats [41].

In the present study, *Ae. aegypti* showed higher $KDT_{50}$ and $KDT_{95}$ as compared to the study conducted in Sudan to deltamethrin insecticide. For instance, the $KDT_{50}$ and $KDT_{95}$ recorded for deltamethrin in Sudan were 13 and 19 minutes respectively which were lower than the current study in Awash Sebat and Werer towns [42]. However, study conducted in Nigeria revealed that the $KDT_{50}$ of deltamethrin was almost ~~two times higher~~ twice than the $KDT_{50}$ ~~results~~ observed in Awash Sebat [43]. A Text Relatively similar permethrin $KDT_{50}$ ~~results were~~ was observed in Awash Sebat town and Mlabani, Tanzania. However, higher KTD95 with deltamethrin and permethrin ~~$KDT_{95}$ results were observed in~~ was found for Mlabani than all the present study towns [34]. Overall, the tube test revealed that *Ae. aegypti* were susceptible to deltamethrin in Sudan and Tanzania but ~~suspected resistance~~ possibly resistant in Nigeria.

*Aedes aegypti* ~~varied in response to public health insecticides~~ was found to show varying susceptibility to diffeent insecticides. *Ae. aegypti* populations were observed to be phenotypic resistant to bendiocarb ~~where the mortality rates were less than 90%~~ with < 90% mortality. *Ae. aegypti* resistance to bendiocarb was also reported in a previous study in

Tanzania during the rainy season [34]. However, these finding were in contrary to the finding reported from Sudan, Nigeria and Cambodia [42-44]. World Health Organization in 2022 has recommended that the insecticide discriminating dose of bendiocarb for *Ae. aegypti* is changed from its initial 0.1% to 0.2% [45]. Thus, future efficacy studies of bendiocarb should target the 0.2% concentration [45].

In the present study, *Ae. aegypti* ~~mortality against propoxur insecticide was assessed. It was observed that *Ae. aegypti* mortality when exposed to propoxur showed insecticide resistance~~ was found to be resistant to bendiocarb 0.1% concentration in all the study towns. Similar finding was reported in Thailand where the efficacy of *Ae. aegypti* mortality to propoxur was 83% which indicated the phenotypic resistance of *Ae. aegypti* to propoxur in this area [46].

The possible contributing factors to the emergences of phenotypic resistance of *Ae. aegypti* in the present study areas could be frequent uses of ~~insecticide chemicals such as~~ insecticide treated bed net and indoor residual insecticide spraying to control mosquito borne diseases as previously indicated [47]. On the other hand, Ethiopia has long history in utilizing pesticides to increase agricultural production and to improve human health since 1964 [48, 49]. Unfortunately however, agro-pesticides induce insecticides resistance selection pressure in different ways. For instance, ~~insecticides used to control mosquitoes can counteract to agro -pesticides used which have the same~~ pesticides used for agriculture pest control can select resistance to vector control insecticides because of a similar mode of action and bio-active ingredients might drive a selection pressure to chemical based insecticide control tools [27]. Similarly, agro-pesticide ~~used in agriculture~~ may ~~ease~~ increase insecticide tolerance as the pesticides washed to mosquito breeding habitats which confer resistance mechanism at the adult stages [50]. Thus, the emergence of insecticide resistance in bendiocarb and propoxur insecticides in the present study could potentially arise from the frequent ~~exposer~~ exposure of the mosquito to carbamate insecticides in agriculture used as an agro-pesticides or herbicides. In addition, *Ae. aegpti*

resistance to propoxur and bendiocarb may occur as a result of mutation in the insecticide target site enzyme Acetyl-cholinesterase (AChE) as reported in Cuba [51].

The pyerethroids such as deltamethrin are ~~chemical insecticides~~ commonly used to control malaria and other mosquito borne diseases in Ethiopia. In the present study, the pyrethroid alpha-cypermethrin showed less than 97% of mortality of adult *Ae. aegypti* populations in the Awash Arba town which revealed the emergence of possible resistance in *Ae. aegypti* in this area. , which may be due to ~~The emergence of possible resistance of *Ae. aegypti* mosquitoes in Awash Arba may be~~ the frequent use of pyrethroids in the insecticide treated nets to control malaria in this area and agro-pesticides as discussed above. This may lead to failure of ~~failer to~~ vector control interventions programs in the study area and elsewhere in the country if resistance management was not undertaken. Similar findings were reported in Ghana [23].

Complete susceptibility of *Ae. aegypti* populations were recorded to the organophosphate ~~chemical insecticide~~ i.e. pirimiphos-methyl in all the study areas. Likewise, the study conducted in Cambodia [44] reported similar findings in that the adult *Ae. aegypti* populations were completely susceptible for the pirimiphos-methyl even in its lower concentration than in the present study. However, this finding was in contrary to the findings reported in Nigeria [43]. Aedes aegypti were fully susceptible to deltamethrin and permethrin ~~Deltamethrin and permethrin of the pyrethroid insecticides were also showed 100% mortality to adult *Ae. aegypti* mosquito populations~~ in the study areas. These findings were in contrary to the findings reported in Malaysia [52] where resistance phenotyp e were observed in permethrin and deltamethrin. However, similar findings were reported in Tanzania [34] and in Peru [53] where deltamethrin showed greater than 99% of mortality in adult *Ae. aegypti*. Thus, these findings could serve as a baseline to the Ethiopian ministry of health and the Afar region health bureau to implement the chemical interventions particularly the pyrethroids deltamethrin, permethrin and the organophosphate pirimiphos-methyl either

in the form of aerial spray or surface treatment to reduce the burden of arboviral diseases during diseases outbreak in different parts of the country.

# Conclusion

The present study ~~reported~~ showed that *Ae. aegypti* populations ~~showed insecticide resistance~~ resistant to bendiocarb and propoxur and ~~possible resistance~~ possibly resistant to alpha-cypermethrin and ~~complete susceptibility~~ fully susceptible to deltamethrin, permethrin and pirimiphos-methyl insecticides. Thus, based on the findings, ~~the following points would be forwarded. Continuous surveillance~~ regular monitoring of insecticide resistance s of *Aedes* ~~species susceptibility to insecticides~~ should be carried out in the study areas and elsewhere in Ethiopia including molecular aspects to mark and evaluate resistance pattern. Moreover, proper uses of existing insecticides are mandatory to ~~hinder~~ prevent *Ae. aegypti* resistance to insecticides in the study areas. In addition, the organophosphate insecticide pirimiphos-methyl can be used in addition to deltamethrin and permethrin in the control of adult *Ae. aegypti* mosquitoes in Ethiopia.

# Acknowledgement

The authors would like to acknowledge Aklilu Lemma Institute of Pathobiology Addis Ababa University, Ethiopia for providing us field and laboratory materials and insecticide impregnated papers. We also like to gratitude the vector biology and control units staffs, Mr Wosen Sisay for his technical assistance in the insectary. Finally, we appreciated all the communities and data collectors from Awash Sebat, Awash Arba and Werer towns for their collaboration during the study period.

**Author contributions**

**Conceptualization:** Mohammed Seid, Esayas Aklilu and Abebe Animut

**Formal Analysis:** Mohammed Seid

**Investigations:** Mohammed Seid, Esayas Aklilu and Abebe Animut

**Methodology:** Mohammed Seid, Esayas Aklilu and Abebe Animut

**Supper-vision**: Mohammed Seid, Esayas Aklilu and Abebe Animut

**Validation:** Mohammed Seid, Esayas Aklilu and Abebe Animut
**Visualization:** Mohammed Seid, Esayas Aklilu and Abebe Animut
**Writing original draft:** Mohammed Seid
**Writing, reviewing and editing:** Mohammed Seid, Esayas Aklilu and Abebe Animut

# References

1. Braack L, et al. Mosquito-borne arboviruses of African origin: review of key viruses and vectors. Parasit vectors. 2018; 11(29):1-26. doi: 10.1186/s13071-017-2559-9 PMID: 29316963
2. Langat SK, et al. Origin and evolution of dengue virus type 2 causing outbreaks in Kenya: Evidence of circulation of two cosmopolitan genotype lineages. Virus Evolution. 2020; 6(1):1-9. doi: 10.1093/ve/veaa026 PMID: 32523778
3. Gutu MA, et al. Another dengue fever outbreak in Eastern Ethiopia-An emerging public health threat. PLoS Negl Trop Dis. 2021; 15(1):1-16. doi:10.1371/journal.pntd.0008992 PMID: 33465086
4. Usman A, et al. Dengue fever outbreaks in Eritrea, 2005-2015. Glob health research policy. 2016; 1(17):1-8. doi: 10.1186/s41256-016-0016-5 PMID: 29202065
5. Serie C, et al. Studies on yellow fever in Ethiopia. Epidemiologic study. Bulletin of the World Health Organization. 1968; 38(6):879-884. PMID: 53036636
6. Lilay A, et al. Re-emergence of yellow fever in Ethiopia after 50 years, 2013: epidemiological and entomological investigations. BMC Infect Dis. 2017; 17(343):1-6. doi: 10.1186/s12879-017-2435-4 PMID: 28506254
7. Woyessa AB, et al. The first acute febrile illness investigation associated with dengue fever in Ethiopia, 2013: a descriptive analysis. The Ethio J Health Dev. 2014; 28(3):155-161.
8. Mekuriaw W, et al. Epidemiological, entomological and climatological investigation of the 2019 dengue fever outbreak in Gewane district, Afar Region, North-east

Ethiopia. Insects. 2022;13(1066):1-13. doi: 10.3390/insects13111066 PMID: 36421969

9. Geleta D, et al. Epidemiological description of chikungunya virus Outbreak in Dire Dawa Administrative City, Western Ethiopia, 2019. Int J Clin Exp Med Sci. 2020; 6(3): 41-45. doi: 10.11648/j.ijcems.20200603.13

10. Alayu M, et al. Risk factors for Chikungunya outbreak in Kebridhar City, Somali Ethiopia, 2019. Unmatched case-control study. bioRxiv. 2020:1-20.

11. Gubler DJ. Dengue, urbanization and globalization: the unholy trinity of the 21st century. Trop Med and health. 2011; 39(4SUPPLEMENT):3-11. doi: 10.2149/tmh.2011-S05 PMID: 22500131.12.

12. Li Y, et al. Urbanization increases *Aedes albopictus* larval habitats and accelerates mosquito development and survivorship. PLoS Negl Trop Dis. 2014; 8(11): 1-12. doi: 10.1371/journal.pntd.0003301 PMID: 25393814

13. WHO. Dengue: guidelines for diagnosis, treatment, prevention and control. World Health Organization. 2009;1-148. PMID: 2376296314

14. Powell JR. Mosquito-borne human viral diseases: why *Aedes aegypti*? The Am J Tro Med and Hyg. 2018; 98(6):1563-1565.doi: 10.4269/ajtmh.17-0866 PMID: 29557341

15. Mattingly P. Taxonomy of *Aedes aegypti* and related species. Bulletin of the World Health Organization. 1967;36:552-554. PMID: 43835441616.

16. Rose NH, et al. Climate and urbanization drive mosquito preference for humans. Current Biol. 2020; 30(18): 1-29. doi: 10.1016/j.cub.2020.06.092 PMID: 32707056

17. Getachew D, et al. Breeding sites of *Aedes aegypti*: potential dengue vectors in Dire Dawa, East Ethiopia. Interd Perspectives Infect Dis. 2015; 2015:1-8. doi: 10.1155/2015/706276 PMID: 2643571218

18. Ferede G, et al. Distribution and larval breeding habitats of *Aedes* mosquito species in residential areas of northwest Ethiopia. Epidemiol and health. 2018; 40: 1-7. doi: 10.4178/epih.e2018015 PMID: 29748457

19. Woyessa A, et al. *Aedes* Mosquitoes Distribution and Risk of Yellow fever transmission in Gurage Zone South-west Ethiopia. Ethio J Publ Health and Nutrition. 4(1):1-10.

20. WHO. Monitoring and managing insecticide resistance in *Aedes* mosquito populations: interim guidance for entomologists. 2016;1-11.

21. Moyes CL, et al. Contemporary status of insecticide resistance in the major *Aedes* vectors of arboviruses infecting humans. PLoS Negl Trop Dis. 2017; 11(7): 1-20. doi: 10.1371/journal.pntd.0005625 PMID: 28727779

22. WHO. Global plan for insecticide resistance management in malaria vectors 2012;13-132.

23. Owusu-Asenso CM, et al. Spatiotemporal distribution and insecticide resistance status of *Aedes aegypti* in Ghana. Parasit Vectors. 2022; 15(61):1-14. doi: 10.1186/s13071-022-05179-w PMID: 35183249

24. Mathias L, et al. Habitat productivity and pyrethroid susceptibility status of *Aedes aegypti* mosquitoes in Dar es Salaam, Tanzania. Infect Dis Poverty. 2017; 6(102):1-10. doi: 10.1186/s40249-017-0316-0 PMID: 2859565325.

25. Kenea O, et al. Impact of combining indoor residual spraying and long-lasting insecticidal nets on *Anopheles arabiensis* in Ethiopia: results from a cluster randomized controlled trial. Malaria J. 2019; 18(182):1-11. doi: 10.1186/s12936-019-2811-1 PMID: 31126286

26. Messenger LA, et al. Insecticide resistance in *Anopheles arabiensis* from Ethiopia (2012–2016): a nationwide study for insecticide resistance monitoring. Malaria J. 2017; 16(469):1-14. doi: 10.1186/s12936-017-2115-2 PMID: 2915102427.

27. Nkya TE, et al. Impact of environment on mosquito response to pyrethroid insecticides: facts, evidences and prospects. Insect biochemistry and molecular biol. 2013; 43(2013):407-416. doi: 10.1016/j.ibmb.2012.10.006 PMID: 23123179

28. Mekuyie M, Jordaan A and Melka Y. Understanding resilience of pastoralists to climate change and variability in the Southern Afar Region, Ethiopia. Climate Risk Managt. 2018; 20: 64-77.

29. Gebru H, Ahmed J. Water quality assessments as implication to human health: a case study in awash sebat and its surrounding areas, Afar, Ethiopia. Inter J Earth Sci and Engi. 2016; 9(5):1999-2008.

30. Goshu D, et al. Socioeconomic Development in Afar Region: Achievements, Gaps and Priorities. 2021.

31. Ministry of Health Ethiopia. Vector control operational manual for malaria elimination in Ethiopia. 2017; 1-56.

32. Sileshi G, Zemedkun A and Nurhussien S. Assessment of the pesticide use, practice and hazards on cotton growing farmers in the Awash Valley Ethiopia. AgriRxiv. 2021(2021): 1-22. doi: https://cabidigitallibrary.org

33. Rueda LM. Pictorial keys for the identification of mosquitoes (Diptera: Culicidae) associated with dengue virus transmission. Walter Reed Army Inst of Research Washington Dc Department of Entomology. 2004;1-60.doi: 10.11646/zootaxa.589.1.1

34. Kahamba NF, et al. Habitat characteristics and insecticide susceptibility of *Aedes aegypti* in the Ifakara area, south-eastern Tanzania. Parasit Vectors. 2020; 13(53) 1-15. doi: 10.1186/s13071-020-3920-y PMID: 32033619

35. Ahmed YM, Salah AA. Epidemiology of dengue fever in Ethiopian Somali region: retrospective health facility based study. Cent Afr J Public Health. 2016; 2(2):51-6.

36. Waldetensai A, et al. *Aedes* mosquito responses to control interventions against the Chikungunya outbreak of Dire Dawa, Eastern Ethiopia. Inter JTrop Insect Sci. 2021; 41:2511-2520. doi: 10.1007/s42690-021-00430-w

37. Diouf B, et al. Resting behavior of blood-fed females and host feeding preferences of *Aedes aegypti* (Diptera: Culicidae) morphological forms in Senegal. J Med Entomol. 2021; 58(6):2467-2473. doi: 10.1093/jme/tjab111 PMID: 34165556

38. Ponlawat A, Harrington LC. Blood feeding patterns of *Aedes aegypti* and *Aedes albopictus* in Thailand. J Med Entomol. 2005; 42(5):844-849. doi: 10.1093/jmedent/42.5.844 PMID: 16363170

39. Diallo D, Diallo M. Resting behavior of *Aedes aegypti* in southeastern Senegal. Parasit Vectors. 2020; 13(356):1-7. doi: 10.1186/s13071-020-04223-x PMID: 32682436

40. Bartlow AW, et al. Forecasting zoonotic infectious disease response to climate change: mosquito vectors and a changing environment. Veter sci .2019; 6(40): 1.20. doi: 10.3390/vetsci6020040 PMID: 31064099

41. Lima EP, MOF Goulart and Rolim Neto ML. Meta-analysis of studies on chemical, physical and biological agents in the control of *Aedes aegypti*. BMC public health. 2015; 15(858):1-14. doi:10.1186/s12889-015-2199-y PMID: 26341708

42. Husham A, Abdalmagid M and Brair M. Status susceptibility of dengue vector; *Aedes aegypti* to different groups of insecticides in Port Sudan City-Red Sea State. Sudanese J Public Health. 2010; 5(4):199-202.

43. Ukpai OM, Ekedo CM. Insecticide susceptibility status of *Aedes aegypti* in umudike, Ikwuano lga Abia State, Nigeria. Animal Research Inter. 2018; 15(3):3082-3089.

44. Boyer S, et al. Monitoring insecticide resistance of adult and larval Aedes aegypti (Diptera: Culicidae) in Phnom Penh, Cambodia. Parasit Vectors. 2022; 15(44): 1-7. doi: 10.1186/s13071-022-05156-3 PMID: 35101104

45. WHO. Manual for monitoring insecticide resistance in mosquito vectors and selecting appropriate interventions. World Health Organization. 2022; 1-55.

46. Pethuan S, et al. Biochemical studies of insecticide resistance in *Aedes* (Stegomyia) *aegypti* and *Aedes* (Stegomyia) *albopictus* (Diptera: Culicidae) in Thailand. Trop Biomed. 2007; 24(1):7-15. PMID: 17568372

47. Barbosa S, et al. Modelling the impact of insecticide-based control interventions on the evolution of insecticide resistance and disease transmission. Parasit vectors. 2018; 11(482):1-21. doi: 10.1186/s13071-018-3025-z PMID: 30153869

48. Mengistie B. Policy-practice nexus: pesticide registration, distribution and use in Ethiopia. SM J Environ Toxicol. 2016; 2(1):1006-1019.

49. Negatu B, Dugassa S, Mekonnen Y. Environmental and health risks of pesticide use in Ethiopia. J Health Pollution. 2021;11(30):1-12. doi: 10.5696/2156-9614-11.30.210601 PMID: 34267988

50. Poupardin R, et al. Cross-induction of detoxification genes by environmental xenobiotics and insecticides in the mosquito *Aedes aegypti*: impact on larval tolerance to chemical insecticides. Insect biochemistry molecular biolo. 2008; 38(2008):540-551. doi: 10.1016/j.ibmb.2008.01.004 PMID: 18405832

51. Bisset J, Rodríguez MM, Fernández D. Selection of insensitive acetylcholinesterase as a resistance mechanism in *Aedes aegypti* (Diptera: Culicidae) from Santiago de Cuba. J Med Entomol. 2006; 43(6):1185-1189. PMID: 17162951

52. Ishak IH, et al. Contrasting patterns of insecticide resistance and knockdown resistance (kdr) in the dengue vectors *Aedes aegypti* and *Aedes albopictus* from Malaysia. Parasit vectors. 2015; 8(181):1-13. doi:10.1186/s13071-015-0797-2 PMID: 25888775

53. Pinto J, et al. Susceptibility to insecticides and resistance mechanisms in three populations of *Aedes aegypti* from Peru. Parasit vectors. 2019; 12(494):1-11. doi: 10.1186/s13071-019-3739-6 PMID: 31640810

## Supporting information

**S1 Table. Observed knockdown times (in minutes) and number of knocked-down female *Ae. aegypti* exposed to insecticides during the 60 minutes in Awash Sebat, Awash Arba and Werer towns of Southern Afar, July to October 2022.**

**S2 Table. ~~Female *Ae. aegypti* mortalities exposed to insecticides observed after 24 hrs of recovery period from~~** Mortality of Ae. aegypti 24 h after 1 h exposure in **Awash Sebat, Awash Arba and Werer towns, Southern Afar Regional State, Ethiopia, July to October, 2022.**

**S3 Table. Representative GPS points of the Awash Sebat, Awash Arba and Werer towns of Southern Afar Regional State, Ethiopia recorded during *Ae. aegypti* larvae and pupae collection.**

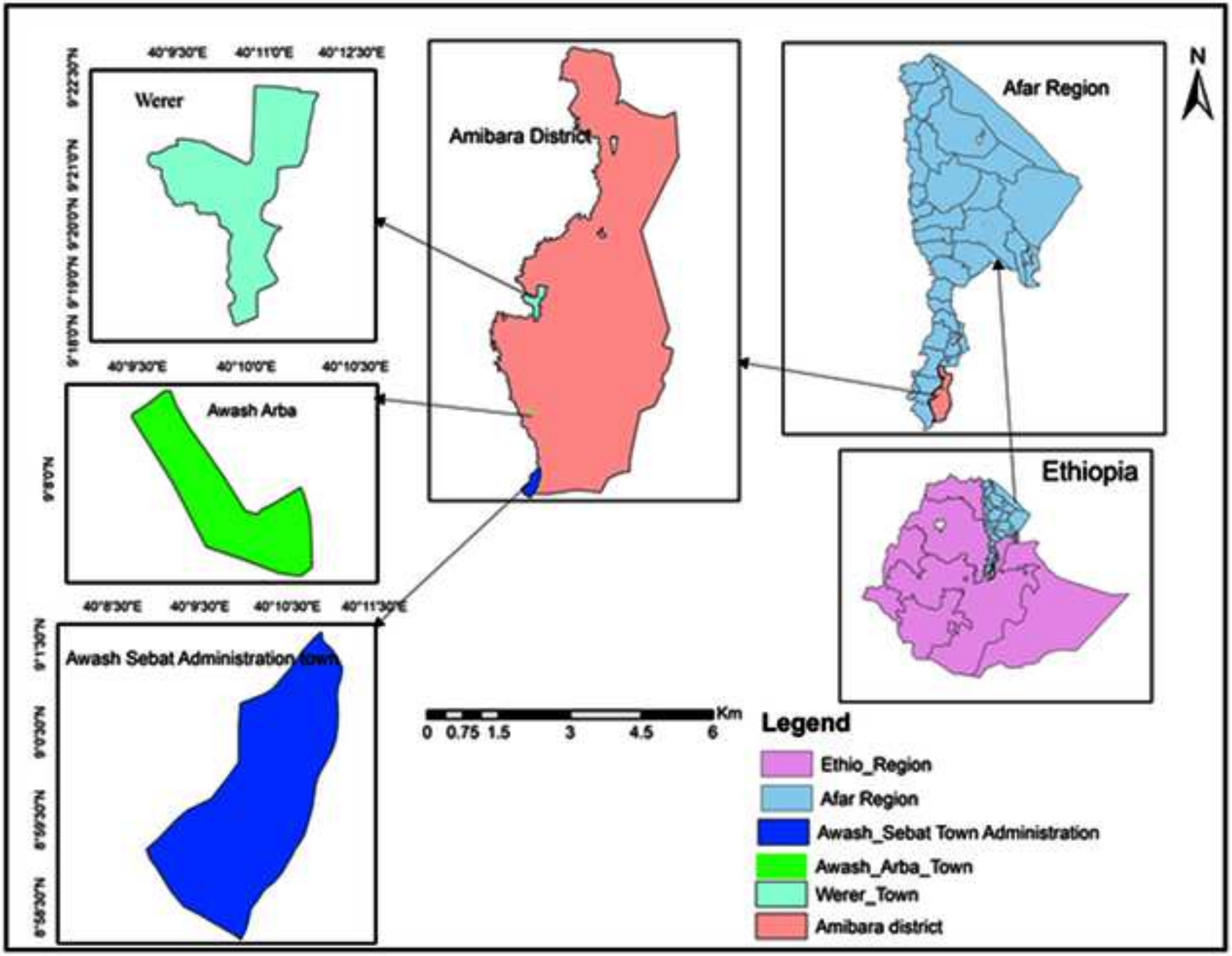

Werer
Awash Arba
Awash Sebat Administration town
Amibara District
Afar Region
Ethiopia
N
Km
0 0.75 1.5 3 4.5 6
Legend
Ethio_Region
Afar Region
Awash_Sebat Town Administration
Awash_Arba_Town
Werer_Town
Amibara district

Click here to access/download;Figure;Fig 2. tiff.tif

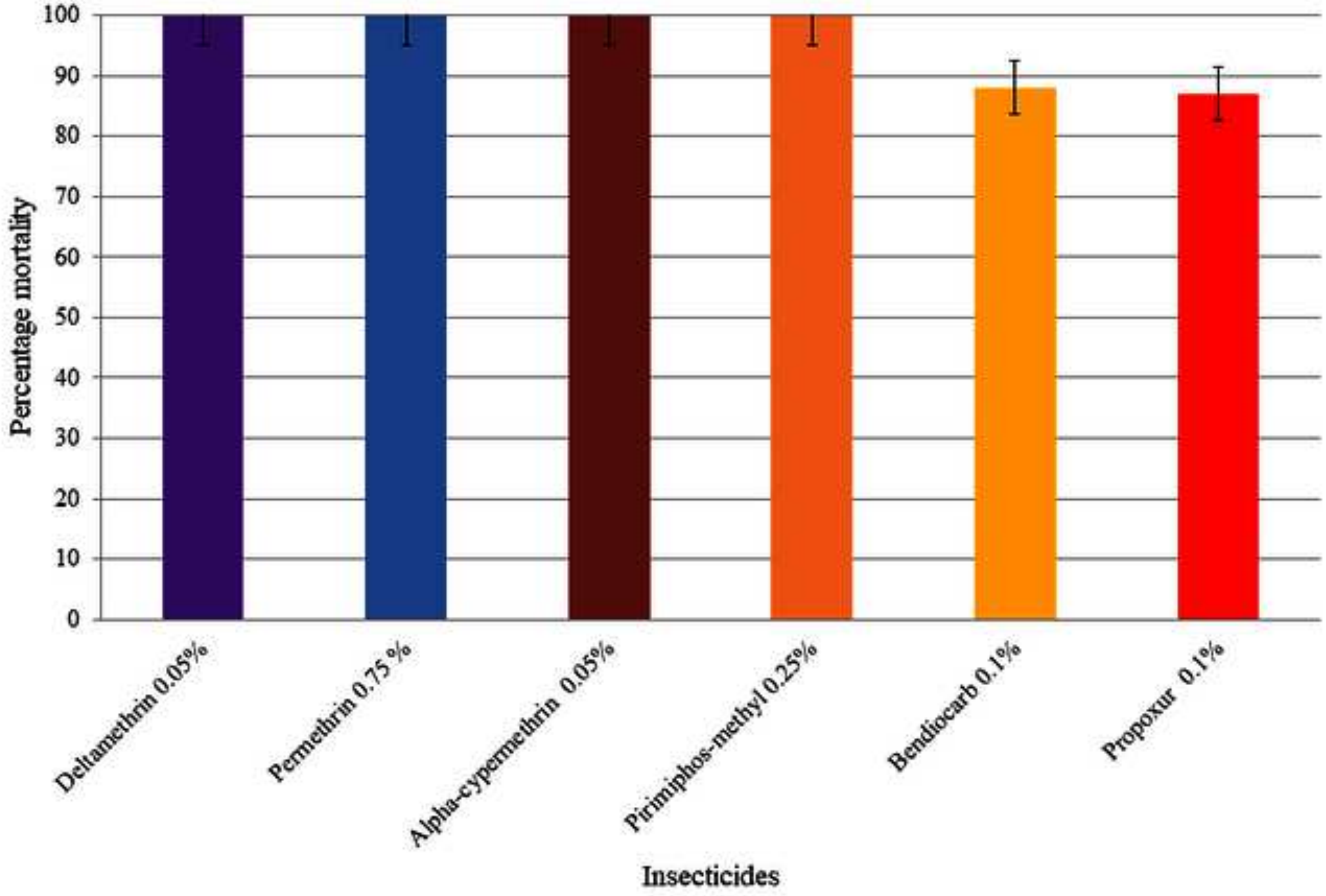

Click here to access/download;Figure;Fig 3. tiff.tif

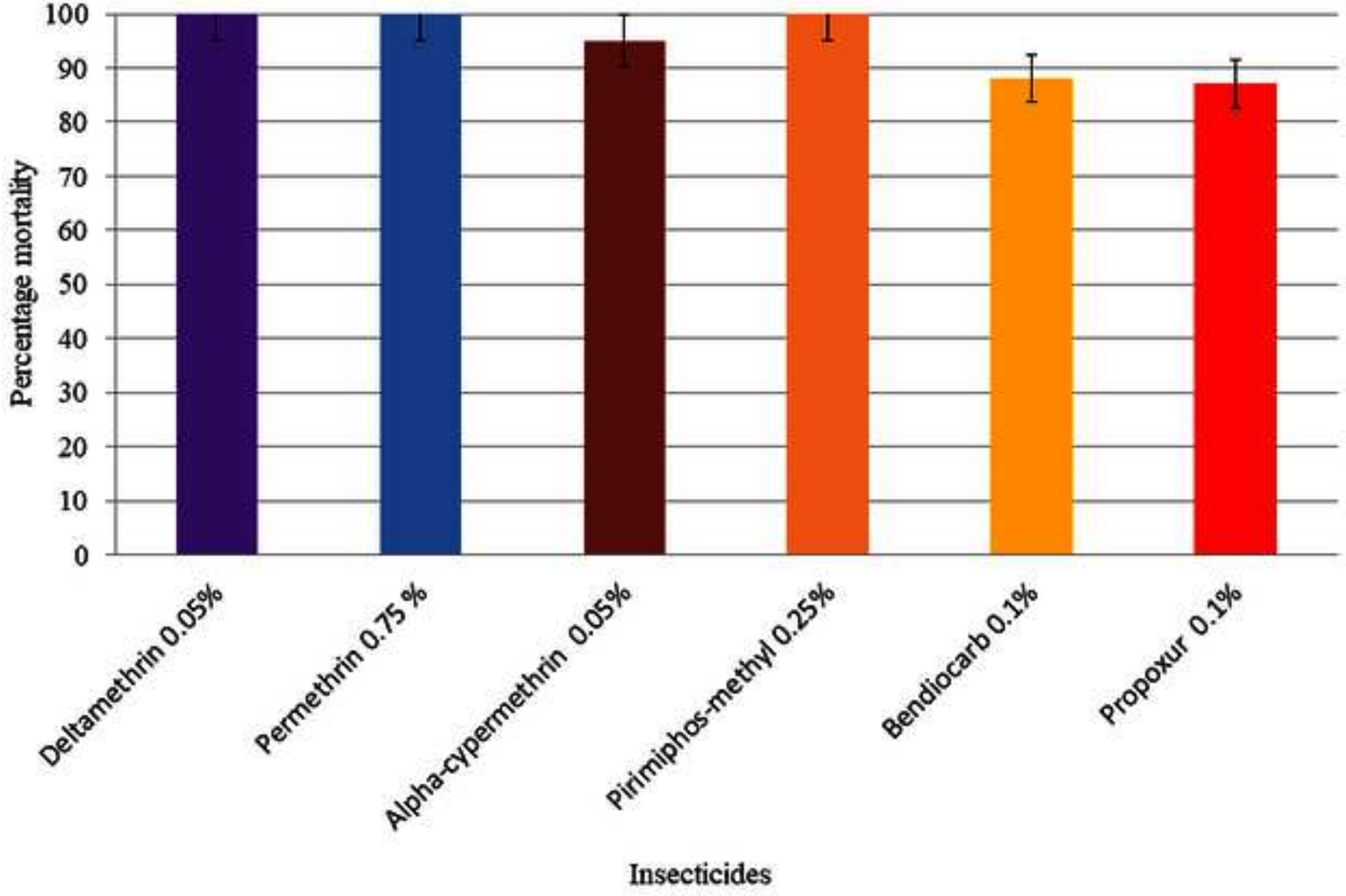

Click here to access/download;Figure;Fig 4. tiff.tif

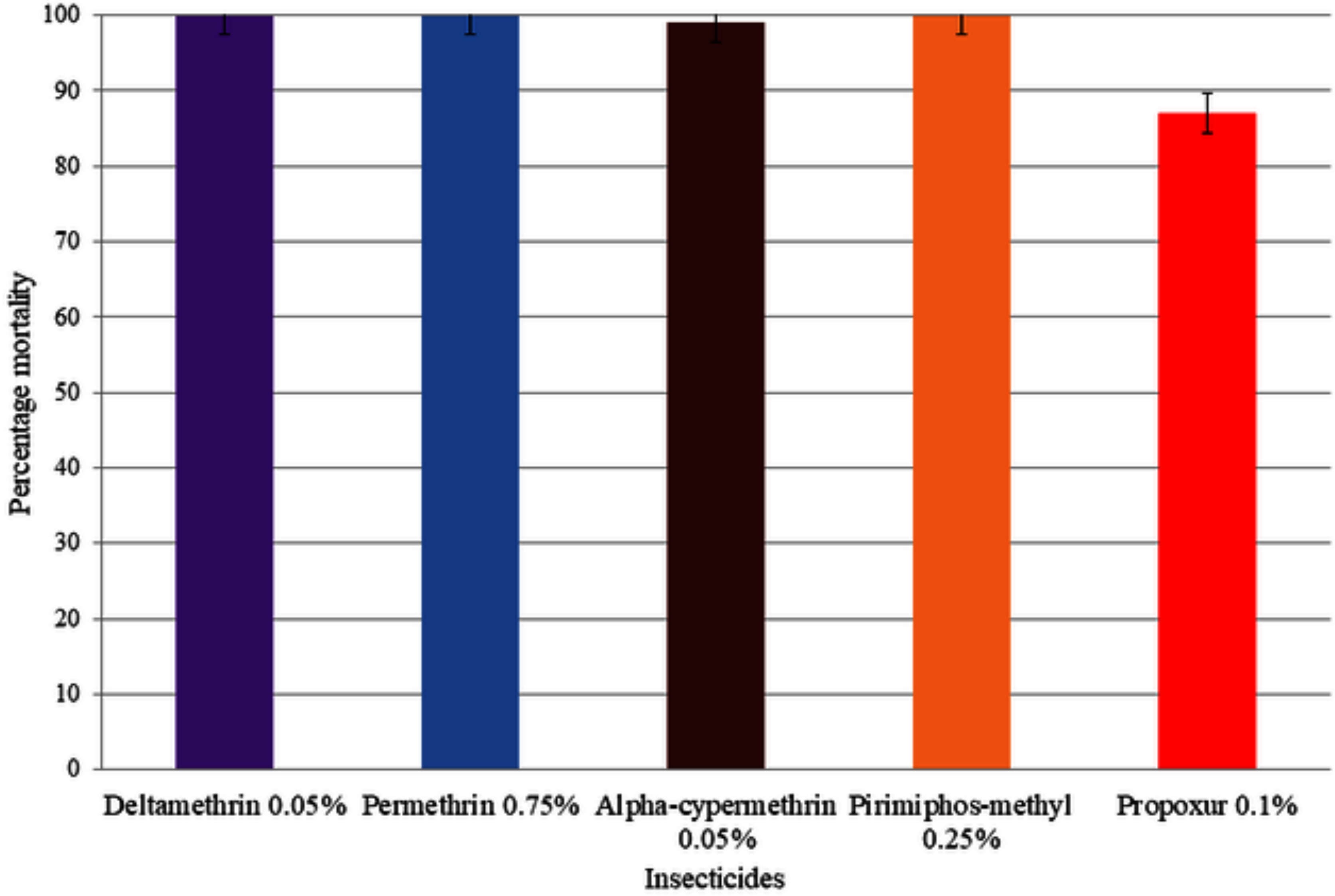

Click here to access/download
**Supporting Information**
S1_Table DOC.docx

Click here to access/download
**Supporting Information**
S2_Table DOC.docx

Click here to access/download
**Supporting Information**
S3_Table DOC.docx

**Susceptibility status of *Aedes aegypti* (Diptera: Culicidae) to public health insecticides in Southern Afar region, Ethiopia**

Mohammed Seid[1, 2*], Esayas Aklilu[1], Abebe Animut[1]

[1] Aklilu Lemma Institute of Pathobiology, Addis Ababa University, Addis Ababa, Ethiopia

[2] Department of Biology, College of Natural and Computational Sciences, Mattu University, Mattu, Ethiopia

*Corresponding author

Email: mohammed.seid.legas@gmail.com (MS)

# Abstract

Mosquito-borne viral diseases such as dengue fever, chikungunya and yellow fever have been documented in Ethiopia since the 1960s. However, efficacy of public health insecticides against *Aedes aegypti* that transmit these viruses remain poorly understood in the country particularly in the Afar region. Thus, the aim of this study was to assess the susceptibility status of *Ae. aegypti* to deltamethrin, permethrin, alpha-cypermethrin, pirimiph os-methyl, bendiocarb and propoxur insecticides. Larvae and pupae of *Aedes* mosquitoes were collected from the Awash Arba, Awash Sebat and Werer towns of the Afar Regional State of Ethiopia during July to October 2022, brought to Aklilu Lemma Institute of Pathobiology, insectary and reared to adults. Non-blood-fed 3 to 5 days old female *Ae. aegypti* were exposed to pyrethroids, carbamates and organophosphate insecticides following the World Health Organization standard guidelines. Knockdown rates were noted at 10 min interval until one hour. The mortality in mosquitoes was recorded 24 hours after 60 minutes of exposure. Mortalities of *Ae. aegypti* exposed to propoxur were 87% in all the study towns. Similarly, 88% of *Ae. aegypti* mortality was recorded when treated with bendiocarb in Awash Sebat and Awash Arba towns. Suspected resistance of *Ae. aegypti* (95% mortality) exposed t o alpha-cypermethrin ~~were~~ was recorded from Awash Arba town. However, *Ae. aegypti* collected from all the three sites ~~were~~ was observed to be susceptible to deltamethrin,

Commented [u1]: Reviewer 2 comment

Commented [u2]: Reviewer 2 comment

Commented [u3]: Reviewer 2 comment

permethrin, and pirimiphos-methyl. *Aedes aegypti* was resistant to 0.1% bendiocarb and 0.1% propoxur, and possibly resistant to 0.05% alpha-cypermethrin. On the other hand, it was susceptible to 0.05% deltamethrin, 0.75% permethrin and 0.25% pirimiphos-methyl. Thus, deltamethrin, permethrin and pirimiphos-methyl can be used in the control of adult *Ae. aegypti* in the Afar Region of Ethiopia. However, further studies should be carried out to evaluate the susceptibility of *Ae. aegypti* to alpha-cypermethrin insecticide in the Awash Arba area.

---

# Introduction

The ever increasing outbreaks of arboviral diseases drew public attention in several countries of the world [1]. The East African countries including Kenya, Somalia, Djibouti, Eritrea and Ethiopia are epicentres of outbreaks of arboviral diseases such as dengue fever, yellow fever and chikungunya virus [2-4]. In Ethiopia, yellow fever caused about 30,000 deaths from over 200,000 infections during the 1960 and 1962 [5]. Recently, there have been yellow fever outbreaks in South Omo [6], dengue fever outbreaks in Dire Dawa city administration, Somali Regional State, Gewane and Amibara districts of Afar Regional State [7, 8]. In addition, chikungunya outbreaks were also reported in Dire Dawa city administration, Afar and Somali Regional States [9, 10]. All these outbreaks with potentially unrecognized and/or under reported deaths owing to the highly limited laboratory facilities in the country.

Mosquitoes transmit over 90% of the viral pathogens through their bites among which *Ae. aegypti* is the leading vector followed by *Ae. albopictus* [11, 12]. *Ae. aegypti* is anthropophilic in its behaviour and occurs in close proximity to human habitations [13, 14]. *Ae. aegypti* has two morphological subspecies (ecotypes) the domestic *Ae. aegypti aegypti* and sylvan *Ae. aegypti formosus* [15, 16]. In Ethiopia, *Ae. aegypti, Ae. bromeliae, Ae.*

*vittatus*, *Ae. hirsutus*, *Ae. simpsoni* complex and *Ae. africanus* are implicated as the potential vectors of viral diseases [6, 8, 17-19].

In the absence of effective therapeutic drugs and vaccines to treat and control mosquito borne viral diseases, preventions largely depend on vector control tools mainly public health insecticides [13]. The pyrethroids such as deltamethrin, alpha-cypermethrin, permethrin and organophosphates pirimiphos-methyl and fenitrothion are among the public health insecticides used against *Aedes* mosquitoes [20]. However, the potential resistance to the insecticides may adversely affect implementation of the control strategy in endemic areas [21]. Mosquitoes could resist insecticides through their inherent ability to detoxify and avoid contacts either behaviourally or physiologically [22].

Evidences from many African countries indicate that the *Anopheles* and *Aedes* mosquitoes developed resistance to insecticides [23-25]. For instance in Ethiopia, malaria transmitting *Anopheles arabiensis* has developed resistance to insecticides in several areas of the country including in the Amibara district of the Afar Regional State [25, 26]. The causes for the resistance could be frequent use of the insecticide chemicals in indoor residual spraying and long-lasting insecticidal treated nets for the control of mosquito borne diseases in the area [21] or in argro-pesticides [27]. However, the susceptibility status of *Aedes* mosquitoes to insecticides currently used is lacking in the country.

Recently, the Ethiopian Public Health Institute prepared arboviral disease vectors, surveillance and control guideline in 2021 through chemical and environmental management methods. Vector control by chemical method such as fumigation, repellents and residual insecticides surface treatments were implemented in Dire Dawa city administration and Somalia Regional State during dengue fever and chikungunya outbreaks (EPHI, 2021 unpublished). Unfortunately, there is a scarcity of evidences on the efficacy of the insecticides for the control of virus transmitting *Aedes* species in the Southern Afar Region in

**Commented [u4]:** Reviewer 2 comment

the presence of increased reports of viral diseases incidence and prevalence. Therefore, assessing the effectiveness of the existing public health insecticides to control *Aedes* species is crucial to strengthen the arboviral diseases prevention and control programs in the country. This inspired us to generate ~~base line~~ baseline data in relation to the applicability of insecticide control tools in the prevention and control of virus transmitting *Aedes* mosquitoes in the area. Therefore, the aim of the study was to assess the susceptibility status of *Ae. aegypti* to public health insecticides in the Southern part of the Afar Regional State of Ethiopia.

**Commented [u5]:** Reviewer 2 comment

# Materials and Methods

**Study sites**

The study was carried out in Awash Arba, Werer and Awash Sebat towns, Gabi-Rasu Zone (Zone 3), of Afar Regional State, Ethiopia. The Awash Arba and Werer towns are located about 250 km to the North-east of Addis Ababa at 9.33453$^{o}$N latitude, 40.181385$^{o}$E longitude and an altitude range of 720 metres above sea level (masl) to 1100 masl (Central statistics Agency, 2019 unpublished). Awash Arba and Werer sites were selected from Amibara district which are about 30 km distance far apart (Fig 1). Agro-ecologically, these two towns are semi-arid with temperature ranging from 25 °C to 35 °C, and average annual rainfall of 530 mm. The communities livelihoods are characterized as pastoralist and agro-pastoralist in which they mainly obtained income on livestock and from livestock and other agricultural crop production [28].

Awash Sebat town administration is located 214 km North-east of Addis Ababa at 8.98810$^{o}$N latitude and 40.163936$^{o}$E longitude (Central statistics Agency, 2019 unpublished). Its altitude ranges from 820 masl to 1120 masl and the temperature varies from 22.6 $^{o}$C to 30.6 $^{o}$C. The town and its surroundings receive a mean annual rainfall of 606.6 mm.

The study areas are found in the middle Awash Valley, where infectious disease such as malaria, chikungunya and dengue fever are common [29]. Considerable attention has been given by the regional government to achieve better health in the region including the three towns [30]. The region has hospitals, health centres, clinics and health posts in different zones and districts. There are one hospital, one clinic and five health centres and few private clinics. Insecticide such as deltamethrin, permethrin, alpha-cypermethrin, pirimiphos-methyl bendiocarb and propoxur are used as insecticide residual spraying and/or long lasting insecticide treated nets for the control of malaria and arboviral diseases in these towns [26, 31]. Similarly, agro-chemicals and agro-pesticides are used since maize and sorghum productions are common in these areas which is used to improve agricultural crop production [32].

**Fig 1. Map of the study areas.** A map showing the locations of the three study areas**:** Awash Sebat, Awash Arba and Werer Towns, in the Southern Afar Region, Ethiopia.

**Larval collection and rearing**

A cross-sectional study was designed to collect larvae and pupae of *Aedes* species from July to October, 2022 in the Awash Sebat, Awash Arba and Werer towns. The study towns were selected purposively in collaboration with the local district health professionals and on the basis of the recent outbreak reports of dengue fever and chikungunya virus (Awash Sebat town and Amibara district health bureaus, 2022 unpublished). The towns were surveyed once per month. Larvae and pupae were collected from different positive habitats including tyres, water storage drums, discarded plastics and metallic containers using standard dipper (350 ml), ladles and pipets/nets. The collected larvae and pupae, from each habitat, were transferred with labelled plastic jars, transported to the insectary of Aklilu Lemma Institute of Pathobiology, Addis Ababa University, Ethiopia and reared to adults under standard laboratory conditions. Tetra cichlid floating fish food (®/TM/©2019 Germany) was provided

as food to the larvae. The emergence of larvae in to pupae was checked each morning and pupae were collected in glass flasks and transferred in to (30cm x 30cm x 30cm) netted cages. Emerged adult *Ae. aegypti* were kept at 27 ± 2 °C temperature, 75 ± 10% relative humidity and fed on 10% sucrose solution until the bioassay tests. Emergent adults were identified to species using morphological taxonomic keys for *Aedes* species [33].

**Phenotypic susceptibility status of *Aedes aegypti***

Prior to insecticide susceptibility tests, sugar fed female *Ae. aegypti* were starved for 2hrs in order to prevent them from having high weight which leads them to be knocked-down. Non-blood-fed, 3-5 days old female *Ae. aegypti* were tested for their susceptibility to insecticides following the World Health Organization insecticide testing procedures [20]. They were exposed to pyrethroids (0.05% deltamethrin, 0.05% alpha-cypermethrin, 0.75% permethrin), carbamates (0.1% bendiocarb and 0.1% propoxur) and organophosphates (0.25% pirimiphos -methyl) which are commonly used for preventing disease transmitting mosquitoes [20]. The insecticides were selected for the study because they have been used for the control of public health important mosquitoes in Ethiopia and the study area. The insecticides are also among the insecticide classes used in the country. Similar concentrations of insecticides were used to test the susceptibility of *Ae. aegypti* in Tanzania [34]. However, we did not follow the World Health Organization recommended discriminating doses for *Aedes* species due to unavailability of insecticide-impregnated papers at the time of the experiment. This practical obstacle required a strategy that looked into the alternative doses for the insecticides,.

**Commented [u6]:** Reviewer 1 comment

In each test, 20 female *Ae. aegypti* were exposed to each five treatment replicates and two control test tubes making a total of 140 mosquitoes in a single test. The exposed mosquitoes were observed for 60 minutes at an interval of 10 minutes to observe their knockdown. After an hour, the exposed females were transferred into holding tubes and kept for 24 hrs at 27.0 ± 2.0 °C and 75 ± 10% relative humidity and were offered cotton wool pads soaked in 10%

sugar solution. The mortality in mosquitoes was recorded 24 hours after 60 minutes of exposure. *Ae. aegypti* susceptibility test was repeated for alpha-cypermethrin in Awash Arba town since *Ae. aegypti* populations were appeared to be suspect phenotypic resistance to this insecticide. However, in case of Werer town the susceptibility status of *Ae. aegypti* to bendiocarb was not conducted due to insufficient mosquito samples.

**Ethical Issues**

The study protocol obtained ethical approval from the institutional review board (IRB) of Aklilu Lemma Institute of Pathobiology, Addis Ababa University, Ethiopia (June 13, 2022; ALIPB IRB/80/2022). Written permission letters were obtained from the district health bureau and health centres of the towns. The households were informed about the objective of the study and their agreements were sought prior to the larvae/pupae collection.

**Data analysis**

Mortality was determined on the basis of the dead and alive mosquitoes after the 24 hours recovery period. Susceptible were those with a mortality in the range of 98-100%, suspected for resistance were with a mortality 90-97% and resistant if mortality was <90% [20]. ~~The knockdown effect of pyrethroids against the *Ae. aegypti* were recorded during the 60 minutes exposure at an interval of 10 minutes from the start.~~ Probit analysis was used to determine estimated duration at which 50% ($KDT_{50}$) and 95% ($KDT_{95}$) of the exposed *Ae. aegypti* mosquitoes were knocked-down. Overall adult mortalities and knockdown times were computed using SPSS Software version 20.

**Commented [u7]:** Reviewer 2 comment

# Results

**Knockdown effect of insecticides against *Aedes aegypti***

Knockdown time of *Ae. aegypti* exposed to insecticides was depicted in Table 1. The knockdown effect of 50% ($KDT_{50}$) of *Ae. aegypti* exposed to deltamethrin ranged from 12.15 to 17.06 minutes whereas $KDT_{95}$ of the insecticide ranged from 18.74 to 34.01 minutes.

Deltamethrin in *Ae. aegypti* showed high knockdown time ($KDT_{95}$, 34.01 minutes) in Awash Sebat as compared to other towns (Table 1). On the other hand, *Ae. aegypti* exposed to permethrin showed the lowest $KDT_{50}$ in Awash Arba town ($KDT_{50}$, 5.11). Overall, the $KDT_{50}$ and $KDT_{95}$ of *Ae. aegypti* varied among *Ae. aegypti* in knockdown to insecticides across the study localities.

**Table 1 Knockdown times (in minute) of 50% and 95% of *Ae. aegypti* exposed to commonly used insecticides in three localities from July to October 2022 in Southern Afar region Ethiopia**

| Insecticides | Localities | $KDT_{50}$ ~~95%~~[95%CI] | $KDT_{95}$ ~~95%~~ [95%CI] |
|---|---|---|---|
| Deltamethrin | Awash sebat | 17.06 [15.81-18.28] | 34.01 [31.02-38.12] |
| | Awash Arba | 12.15 [11.41-12.97] | 18.74 [16.98-21.56] |
| | Werer | 16.53 [15.37-17.67] | 30.92 [28.24-34.62] |
| Permethrin | Awash Sebat | 8.92 [7.34-10.26] | 24.21 [21.23-28.93] |
| | Awash Arba | 5.11 [2.91-6.94] | 19.23 [16.23-24.09] |
| | Werer | 15.12 [12.45-17.66] | 29.89 [24.64-41.53] |
| Alpha-cypermethrin | Awash Sebat | 14.35 [13.35-15.35] | 24.93 [22.77-27.97] |
| | Awash Arba | 13.47 [12.64-14.36] | 20.78 [19.04-23.32] |
| | Werer | 16.71 [13.85-19.46] | 32.90 [27.26-44.98] |

CI, Confidence interval; $KDT_{50}$, time taken for 50% of the tested Ae. *aegypti* to be knocked-down; $KDT_{95}$ time taken for 95% of the tested *Ae. aegypti* to be knocked-down

**Susceptibility status of *Aedes aegypti* to insecticides**

The percentage mortalities of *Ae. aegypti* mosquito populations against insecticides were~~as~~ presented in figures (F~~f~~igs 2-4). Accordingly, *Ae. aegypti* mosquitoes showed phenotypic resistance to bendiocarb and propoxur insecticides in which the percentage mortalities were recorded to be 88% and 87% respectively in Awash Sebat town. *Ae. aegypti* mortalities to

Commented [u8]: Reviewer 2 comment

Commented [u9]: Only the first letter should be capitalized and no dot was needed as PLOS ONE Journal guideline.

deltamethrin, permethrin alph-acypermthrin and pirimiphos-methyl were 100% in Awash Sebat town (Fig 2). All mosquitoes in the control group showed 0% mortality in all study sites.

**Fig 2. Insecticide susceptibility status of *Ae. aegypti* populations from Awash Sebat town, Southern Afar region, Ethiopia, July to October 2022.** Error bars represent 95% confidence intervals.

In addition, *Ae. aegypti* mosquitoes were also observed to be resistantantce to bendiocarb and propoxur from Awash Arba. Moreover, *Ae. aegypti* mosquitoes were observed to be suspected resistant to alpha-cypermethrin in which the mortality of *Ae. aegypti* mosquitoes due to alpha-cypermethrin was 95% (Fig 3). *Ae. eagypti* mortalities were observed to be 100% when exposed to deltamethrin, permethrin and pirimiphos-methyl insecticides in this town.

**Fig 3. Insecticide susceptibility status of *Ae. aegypti* populations from Awash Arba town, Southern Afar region, Ethiopia, July to October 2022.** Error bars represent 95% confidence intervals.

*Ae. aegypti* mortalities when exposed to propoxur insecticide wereas 87% whereas 99% mortalities were observed among the *Ae. aegypti* mosquitoes from Werer town when treated with alpha-cypermethrin insecticide (Ffig 4). However, 100% mortalities of *Ae. aegypti* from this site were recorded to deltamethrin, permethrin and pirimiphos-methyl.

**Fig 4. Insecticide susceptibility status of *Ae. aegypti* populations in Werer town, Southern Afar region, Ethiopia, July to October 2022.** Error bars represent 95% confidence intervals.

# Discussion

**Commented [u10]:** Reviewer 2 comment

Recent outbreaks of dengue fever and chikungunya virus fever in the Eastern and the North-eastern parts of Ethiopia especially in Dire Dawa city administration, Somali Regional State and Amibara and Gewane districts of Afar regional State requires the need for the control of the *Aedes* species that transmit the viruses. The insecticide based mosquito control is underway in Ethiopia in general and in Afar regional State in particular [7, 35]. In so doing, there have been continuous evaluation of efficacy of the insecticides against malaria transmitting *Anopheles* species but there is scarcity of research findings conducted to control *Aedes* species either chemically, biologically or other means of vector control strategies in the country [36]. Thus, the control of viral disease transmitting *Aedes aegypti* vector is poorly investigated and needs detailed research. As far as our knowledge is concerned, this study was first in its kind to report the susceptibility status of *Ae. aegypti* to public health insecticides in the Southern Afar Regional State and there are limited data to compare our findings to others in the country.

*Aedes aegypti* is generally considered as exophillic, exophagic and day time biters. They usually feed on human and other vertebrate hosts as described in previous studies [37, 38]. However, they may tend to be endophilic and endophagic in which they rest and fed indoors [39]. This makes controlling of the *Ae. aegypti* and the viral diseases they transmit more difficult [40]. As a result, different intervention tools have been adopted to control these mosquitoes including indoor residual insecticide spraying and long lasting insecticide treated nets in addition to managing their breeding habitats [41].

In the present study, *Ae. aegypti* showed higher $KDT_{50}$ and $KDT_{95}$ as compared to the study conducted in Sudan to deltamethrin insecticide. For instance, the $KDT_{50}$ and $KDT_{95}$ recorded for deltamethrin in Sudan were 13 and 19 minutes respectively which were lower than the current study in Awash Sebat and Werer towns [42]. However, study conducted in Nigeria revealed that the $KDT_{50}$ of deltamethrin was almost two times higher than the $KDT_{50}$

results observed in Awash Sebat [43]. Relatively similar permethrin $KDT_{50}$ results were observed in Awash Sebat town and Mlabani, Tanzania. However, higher deltamethrin and permethrin $KDT_{95}$ results were observed in Mlabani than all the present study towns [34]. Overall, the tube test revealed that *Ae. aegypti* were susceptible to deltamethrin in Sudan and Tanzania but suspected resistance in Nigeria.

*Aedes aegypti* varied in response to public health insecticides. *Ae. aegypti* populations were observed to be phenotypic resistant to bendiocarb where the mortality rates were less than 90%. *Ae. aegypti* resistance to bendiocarb was also reported in a previous study in Tanzania during the rainy season [34]. However, these finding were in contrary to the finding reported from Sudan, Nigeria and Cambodia [42-44]. World hHealth oOrganization in 2022 has recommended that the insecticide discriminating dose of bendiocarb for *Ae. aegypti* is changed from its initial 0.1% to 0.2% [45]. Thus, future efficacy studies of bendiocarb should target the 0.2% concentration [45].

In the present study, *Ae. aegypti* mortallymortality against propoxur insecticide was assessed. It was observed that *Ae. aegypti* mortality when exposed to propoxur showed insecticide resistance in all the study towns. Similar findings wasere reported in Thailand where the efficacy of *Ae. aegypti* mortality to propoxur was 83% which indicated the phenotypic resistance of *Ae. aegypti* to propoxur in this area [46].

The possible contributing factors to the emergences of phenotypic resistance of *Ae. aegypti* in the present study areas could be frequent uses of insecticide chemicals such as insecticide treated bed net and indoor residual insecticide spraying to control mosquito borne diseases as previously indicated [47]. On the other hand, Ethiopia has long history in utilizing pesticides to increase agricultural production and to improve human health since 1964 [48, 49]. Unfortunately however, agro-pesticides induce insecticides resistance selection pressure in different ways. For instance, insecticides used to control mosquitoes can counteract to agro

**Commented [u11]:** Reviewer 2 comment

-pesticides used which have the same mode of action and bio-active ingredients might drive a selection pressure to chemical based insecticide control tools [27]. Similarly, agro-pesticide used in agriculture may ease insecticide tolerance as the pesticides washed to mosquito breeding habitats which confer resistance mechanism at the adult stages [50]. Thus, the emergence of insecticide resistance in bendiocarb and propoxur insecticides in the present study could potentially arise from the frequent exposer of the mosquito to carbamate insecticides in agriculture used as an agro-pesticides or herbicides. In addition, *Ae. aegpti* resistance to propoxur and bendiocarb may occur as a result of mutation in the insecticide target site enzyme Acetyl-cholinesterase (AChE) as reported in Cuba [51].

The pyerethroids such as deltamethrin are chemical insecticides commonly used to control malaria and other mosquito borne diseases in Ethiopia. In the present study, the pyrethroid alpha-cypermethrin showed less than 97% of mortality of adult *Ae. aegypti* populations in the Awash Arba town which revealed the emergence of possible resistance in *Ae. aegypti* in this area. The emergence of possible resistance of *Ae. aegypti* mosquitoes in Awash Arba may be the frequent use of pyrethroids in the insecticide treated nets to control malaria in this area and agro-pesticides as discussed above. This may lead failer to vector control interventions programs in the study area and elsewhere in the country. Similar findings were reported in Ghana [23].

Complete susceptibility of *Ae. aegypti* populations were recorded to the organophosphate chemical insecticide i.e. pirimiphos-methyl in all the study areas. Likewise, the study conducted in Cambodia [44] reported similar findings in that the adult *Ae. aegypti* populations were completely susceptible for the pirimiphos-methyl even in its lower concentration than in the present study. However, this finding was in contrary to the findings reported in Nigeria [43]. Deltamethrin and permethrin of the pyrethroid insecticides wereare also showed 100% mortality to adult *Ae. aegypti* mosquito populations in the study

areas. These

findings were in contrary to the findings reported in Malaysia [52] where resistance phenotyp e were observed in permethrin and deltamethrin. However, similar findings wereas reported in Tanzania [34] and in Peru [53] where deltamethrin showed greater than 99% of mortality in adult *Ae. aegypti*. Thus, theise findings could serve as a base-line to the Ethiopian ministry of health and the Afar region health bureau to implement the chemical interventions particularly the pyrethroids deltamethrin, permethrin and the organophosphate pirimiphos-methyl either in the form of aerial spray or surface treatment to reduce the burden of arboviral diseases during diseases outbreak in different parts of the country.

# Conclusion

The present study reported that *Ae. aegypti* populations showed insecticide resistance to bendiocarb and propoxur and possible resistance to alpha-cypermethrin and complete susceptibility to deltamethrin, permethrin and pirimiphos-methyl insecticides. Thus, based on the findings, the following points would be forwarded. Continuous surveillances of *Aedes* species susceptibility to insecticides should be carried out in the study areas and elsewhere in Ethiopia including molecular aspects to mark and evaluate resistance pattern. Moreover, proper uses of existing insecticides are mandatory to hinder *Ae. aegypti* resistance to insecticides in the study areas. In addition, the organophosphate insecticide pirimiphos-methyl can be used in addition to deltamethrin and permethrin in the control of adult *Ae. aegypti* mosquitoes in Ethiopia.

# Acknowledgement

The authors would like to acknowledge Aklilu Lemma Institute of Pathobiology Addis Ababa University, Ethiopia for providing us field and laboratory materials and insecticide impregnated papers. We also like to gratitude the vector biology and control units staffs, Mr Wosen Sisay for his technical assistance in the insectary. Finally, we appreciated all the

communities and data collectors from Awash Sebat, Awash Arba and Werer towns for their collaboration during the study period.

**Author contributions**

**Conceptualization:** Mohammed Seid, Esayas Aklilu and Abebe Animut

**Formal Analysis:** Mohammed Seid

**Investigations:** Mohammed Seid, Esayas Aklilu and Abebe Animut

**Methodology:** Mohammed Seid, Esayas Aklilu and Abebe Animut

**Supper-vision**: Mohammed Seid, Esayas Aklilu and Abebe Animut

**Validation:** Mohammed Seid, Esayas Aklilu and Abebe Animut

**Visualization:** Mohammed Seid, Esayas Aklilu and Abebe Animut

**Writing original draft:** Mohammed Seid

**Writing, reviewing and editing:** Mohammed Seid, Esayas Aklilu and Abebe Animut

## References

1. Braack L, et al. Mosquito-borne arboviruses of African origin: review of key viruses and vectors. Parasit vectors. 2018; 11(291):1-26. doi: 10.1186/s13071-017-2559-9 PMID: 29316963
2. Langat SK, et al. Origin and evolution of dengue virus type 2 causing outbreaks in Kenya: Evidence of circulation of two cosmopolitan genotype lineages. Virus Evolution. 2020; 6(1):1-9. doi: 10.1093/ve/veaa026 PMID: 32523778
3. Gutu MA, et al. Another dengue fever outbreak in Eastern Ethiopia-An emerging public health threat. PLoS Negl Trop Dis. 2021; 15(1):1-16. doi:10.1371/journal.pntd.0008992 PMID: 33465086
4. Usman A, et al. Dengue fever outbreaks in Eritrea, 2005-2015. Glob health research policy. 2016; 1(17):1-8. doi: 10.1186/s41256-016-0016-5 PMID: 29202065
5. Serie C, et al. Studies on yellow fever in Ethiopia. Epidemiologic study. Bulletin of the World Health Organization. 1968; 38(6):879-884. PMID: 53036636

6. Lilay A, et al. Re-emergence of yellow fever in Ethiopia after 50 years, 2013: epidemiological and entomological investigations. BMC Infect Dis. 2017; 17(1):1-6. doi: 10.1186/s12879-017-2435-4 PMID: 28506254

7. Woyessa AB, et al. The first acute febrile illness investigation associated with dengue fever in Ethiopia, 2013: a descriptive analysis. The Ethio J Health Develop. 2014; 28(3):155-161.

8. Mekuriaw W, et al. Epidemiological, entomological and climatological investigation of the 2019 dengue fever outbreak in Gewane district, Afar Region, North-east Ethiopia. Insects. 2022; 13(10661):1-13. doi: 10.3390/insects13111066 PMID: 36421969

9. Geleta D, et al. Epidemiological description of chikungunya virus Outbreak in Dire Dawa Administrative City, Western Ethiopia, 2019. Int J Clin Exp Med Sci. 2020; 6(3): 41-45. doi: 10.11648/j.ijcems.20200603.13

10. Alayu M, et al. Risk factors for Chikungunya outbreak in Kebridhar City, Somali Ethiopia, 2019. Unmatched case-control study. bioRxiv. 2020:1-20.

11. Gubler DJ. Dengue, urbanization and globalization: the unholy trinity of the 21st century. Trop Med and health. 2011; 39(4SUPPLEMENT):31-112. doi: 10.2149/tmh.2011-S05 PMID: 22500131.12

12. Li Y, et al. Urbanization increases *Aedes albopictus* larval habitats and accelerates mosquito development and survivorship. PLoS Negl Trop Dis. 2014; 8(11): 1-12. doi: 10.1371/journal.pntd.0003301 PMID: 25393814

13. WHO. Dengue: guidelines for diagnosis, treatment, prevention and control. World Health Organization. 2009;1-148. PMID: 2376296314

14. Powell JR. Mosquito-borne human viral diseases: why *Aedes aegypti*? The Am J Tro Med and Hyg. 2018; 98(6):1563-1565.doi: 10.4269/ajtmh.17-0866 PMID: 29557341

15. Mattingly P. Taxonomy of *Aedes aegypti* and related species. Bulletin of the World Health Organization. 1967;36(4):552-554. PMID: 43835441616.

16. Rose NH, et al. Climate and urbanization drive mosquito preference for humans. Current Biol. 2020; 30(18): 1-29. doi: 10.1016/j.cub.2020.06.092 PMID: 32707056

17. Getachew D, et al. Breeding sites of *Aedes aegypti*: potential dengue vectors in Dire Dawa, East Ethiopia. Interd Perspectives Infect Dis. 2015; 2015:1-8. doi: 10.1155/2015/706276 PMID: 2643571218

18. Ferede G, et al. Distribution and larval breeding habitats of *Aedes* mosquito species in residential areas of northwest Ethiopia. Epidemiol and health. 2018; 40: 1-7. doi: 10.4178/epih.e2018015 PMID: 29748457

19. Woyessa A, et al. *Aedes* Mosquitoes Distribution and Risk of Yellow fever transmission in Gurage Zone South-west Ethiopia. Ethio J Publ Health and Nutrition. 4(1):1-10.

20. WHO. Monitoring and managing insecticide resistance in *Aedes* mosquito populations: interim guidance for entomologists. 2016;1-11.21.

21. Moyes CL, et al. Contemporary status of insecticide resistance in the major *Aedes* vectors of arboviruses infecting humans. PLoS Negl Trop Dis. 2017; 11(7): 1-20. doi: 10.1371/journal.pntd.0005625 PMID: 28727779

22. WHO. Global plan for insecticide resistance management in malaria vectors 2012;13-132.

23. Owusu-Asenso CM, et al. Spatiotemporal distribution and insecticide resistance status of *Aedes aegypti* in Ghana. Parasit Vectors. 2022; 15(61):1-14. doi: 10.1186/s13071-022-05179-w PMID: 35183249

24. Mathias L, et al. Habitat productivity and pyrethroid susceptibility status of *Aedes aegypti* mosquitoes in Dar es Salaam, Tanzania. Infect Dis Poverty. 2017; 6(102):1-10. doi: 10.1186/s40249-017-0316-0 PMID: 2859565325.

25. Kenea O, et al. Impact of combining indoor residual spraying and long-lasting insecticidal nets on *Anopheles arabiensis* in Ethiopia: results from a cluster randomized controlled trial. Malaria J. 2019; 18(182):1-11. doi: 10.1186/s12936-019-2811-1 PMID: 31126286

26. Messenger LA, et al. Insecticide resistance in *Anopheles arabiensis* from Ethiopia (2012–2016): a nationwide study for insecticide resistance monitoring. Malaria J. 2017; 16(4691):1-14. doi: 10.1186/s12936-017-2115-2 PMID: 2915102427.

27. Nkya TE, et al. Impact of environment on mosquito response to pyrethroid insecticides: facts, evidences and prospects. Insect biochemistry and molecular biol. 2013; 43(20134):407-416. DOI: 10.1016/j.ibmb.2012.10.006 PMID: 23123179

28. Mekuyie M, Jordaan A and Melka Y. Understanding resilience of pastoralists to climate change and variability in the Southern Afar Region, Ethiopia. Climate Risk Managt. 2018; 20: 64-77.

29. Gebru H, Ahmed J. Water quality assessments as implication to human health: a case study in awash sebat and its surrounding areas, Afar, Ethiopia. Inter J Earth Sci and Engi. 2016; 9(5):1999-2008.

30. Goshu D, et al. Socioeconomic Development in Afar Region: Achievements, Gaps and Priorities. 2021.

31. Ministry of Health Ethiopia. Vector control operational manual for malaria elimination in Ethiopia. 2017; 1-56.

32. Sileshi G, Zemedkun A and Nurhussien S. Assessment of the pesticide use, practice and hazards on cotton growing farmers in the Awash Valley Ethiopia. AgriRxiv. 2021(2021): 1-22. doi: https://cabidigitallibrary.org

33. Rueda LM. Pictorial keys for the identification of mosquitoes (Diptera: Culicidae) associated with dengue virus transmission. Walter Reed Army Inst of Research Washington Dc Department of Entomology. 2004;1-60.doi: 10.11646/zootaxa.589.1.1

34. Kahamba NF, et al. Habitat characteristics and insecticide susceptibility of *Aedes aegypti* in the Ifakara area, south-eastern Tanzania. Parasit Vectors. 2020; 13(531) 1-15. doi: 10.1186/s13071-020-3920-y PMID: 32033619

35. Ahmed YM, Salah AA. Epidemiology of dengue fever in Ethiopian Somali region: retrospective health facility based study. Cent Afr J Public Health. 2016; 2(2):51-6.

36. Waldetensai A, et al. *Aedes* mosquito responses to control interventions against the Chikungunya outbreak of Dire Dawa, Eastern Ethiopia. Inter JTrop Insect Sci. 2021; 41:2511-2520. doi: 10.1007/s42690-021-00430-w

37. Diouf B, et al. Resting behavior of blood-fed females and host feeding preferences of *Aedes aegypti* (Diptera: Culicidae) morphological forms in Senegal. J Med Entomol. 2021; 58(6):2467-2473. doi: 10.1093/jme/tjab111 PMID: 34165556

38. Ponlawat A, Harrington LC. Blood feeding patterns of *Aedes aegypti* and *Aedes albopictus* in Thailand. J Med Entomol. 2005; 42(5):844-849. doi: 10.1093/jmedent/42.5.844 PMID: 16363170

39. Diallo D, Diallo M. Resting behavior of *Aedes aegypti* in southeastern Senegal. Parasit Vectors. 2020; 13(3561):1-7. doi: 10.1186/s13071-020-04223-x PMID: 32682436

40. Bartlow AW, et al. Forecasting zoonotic infectious disease response to climate change: mosquito vectors and a changing environment. Veter sci .2019; 6(402): 1.20. doi: 10.3390/vetsci6020040 PMID: 31064099

41. Lima EP, MOF Goulart and Rolim Neto ML. Meta-analysis of studies on chemical, physical and biological agents in the control of *Aedes aegypti*. BMC public health. 2015; 15(858):1-14. doi:10.1186/s12889-015-2199-y PMID: 26341708

42. Husham A, Abdalmagid M and Brair M. Status susceptibility of dengue vector; *Aedes aegypti* to different groups of insecticides in Port Sudan City-Red Sea State. Sudanese J Public Health. 2010; 5(4):199-202.

43. Ukpai OM, Ekedo CM. Insecticide susceptibility status of *Aedes aegypti* in umudike, Ikwuano lga Abia State, Nigeria. Animal Research Inter. 2018; 15(3):3082-3089.

44. Boyer S, et al. Monitoring insecticide resistance of adult and larval Aedes aegypti (Diptera: Culicidae) in Phnom Penh, Cambodia. Parasit Vectors. 2022; 15(441): 1-7. doi: 10.1186/s13071-022-05156-3 PMID: 35101104

45. WHO. Manual for monitoring insecticide resistance in mosquito vectors and selecting appropriate interventions. World Health Organization. 2022; 1-55.

46. Pethuan S, et al. Biochemical studies of insecticide resistance in *Aedes* (Stegomyia) *aegypti* and *Aedes* (Stegomyia) *albopictus* (Diptera: Culicidae) in Thailand. Trop Biomed. 2007; 24(1):7-15. PMID: 17568372

47. Barbosa S, et al. Modelling the impact of insecticide-based control interventions on the evolution of insecticide resistance and disease transmission. Parasit vectors. 2018; 11(482):1-21. doi: 10.1186/s13071-018-3025-z PMID: 30153869

48. Mengistie B. Policy-practice nexus: pesticide registration, distribution and use in Ethiopia. SM J Environ Toxicol. 2016; 2(1):1006-1019.

49. Negatu B, Dugassa S, Mekonnen Y. Environmental and health risks of pesticide use in Ethiopia. J Health Pollution. 2021; 11(30):1-12. doi: 10.5696/2156-9614-11.30.210601 PMID: 34267988

50. Poupardin R, et al. Cross-induction of detoxification genes by environmental xenobiotics and insecticides in the mosquito Aedes aegypti: impact on larval tolerance to chemical insecticides. Insect biochemistry molecular biolo. 2008; 38(20085): 540-551. doi: 10.1016/j.ibmb.2008.01.004 PMID: 18405832

51. Bisset J, Rodríguez MM, Fernández D. Selection of insensitive acetylcholinesterase as a resistance mechanism in *Aedes aegypti* (Diptera: Culicidae) from Santiago de Cuba. J Med Entomol. 2006; 43(6):1185-1189. PMID: 17162951

52. Ishak IH, et al. Contrasting patterns of insecticide resistance and knockdown resistance (kdr) in the dengue vectors *Aedes aegypti* and *Aedes albopictus* from Malaysia. Parasit vectors. 2015; 8(181): 1-13. doi:10.1186/s13071-015-0797-2 PMID: 25888775

53. Pinto J, et al. Susceptibility to insecticides and resistance mechanisms in three populations of *Aedes aegypti* from Peru. Parasit vectors. 2019; 12(4941):1-11. doi: 10.1186/s13071-019-3739-6 PMID: 31640810

## Supporting information

**S1 Table. Observed knockdown times (in minutes) and number of knocked-down female *Ae. aegypti* exposed to insecticides during the 60 minutes in Awash Sebat, Awash Arba and Werer towns of Southern Afar, July to October 2022.**

**S2 Table. Female *Ae. aegypti* mortalities exposed to insecticides observed after 24 hrs of recovery period from Awash Sebat, Awash Arba and Werer towns, Southern Afar Regional State, Ethiopia, July to October, 2022.**

**S3 Table. Representative GPS points of the Awash Sebat, Awash Arba and Werer towns of Southern Afar Regional State, Ethiopia recorded during *Ae. aegypti* larvae and pupae collection.**

**Date 28/05/2024**

**To: James Colborn**

**Academic editor**

**PLOS ONE Journal**

**Sub: Providing point-by-point responses to the Editor’s and reviewers’ comments, questions and suggestions**

Dear James Colborn (Dr./Prof), we are grateful again to the journal **PLOS ONE** for considering our original research manuscript entitled **Susceptibility status of *Aedes aegypti* (Diptera: Culicidae) to public health insecticides in Southern Afar region, Ethiopia** with the manuscript number of PONE-D-24-03915R1 for possible publication. We also appreciated the academic editor and reviewers to their relevant comments and suggestions in the second reviews of the manuscript. The comments and suggestions forwarded are highly valuable to improve the manuscript. We present point-by-point responses to the editor and reviewers’ questions, comments and suggestions below.

**Comments on journal requirements**

1.Please review your reference list to ensure that it is complete and correct. If you have cited papers that have been retracted, please include the rationale for doing so in the manuscript text, or remove these references and replace them with relevant current references. Any changes to the reference list should be mentioned in the rebuttal letter that accompanies your revised manuscript. If you need to cite a retracted article, indicate the article’s retracted status in the References list and also include a citation and full reference for the retraction notice.

***Response:*** **Dear Editor, thank you very much once again for your relevant suggestion. We have reviewed the references cited in the text to their correctness in the references list. We have also corrected a few typos of existed references particularly the volume and issue numbers in the revised manuscript. Retracted references have not been used. We have cited the WHO, 2022 of reference number[45] on future recommendation for efficacy studies of bendiocarb that should target to the 0.2% concentration.**

2. While revising your submission, please upload your figure files to the Preflight Analysis and Conversion Engine (PACE) digital diagnostic tool.

***Response*: We have uploaded the figures to PACE to ensure that the figures meet PLOS requirements.**

**Reviewer #1**

The authors have responded to numerous comments, but there is still a gap in explaining why the recommended discriminating doses for susceptibility tests of *Aedes* mosquitoes, as outlined in the WHO guideline cited in the methodology section, were not followed. This discrepancy must be addressed transparently in the methodology, providing genuine explanations for the deviation. The authors may suggest that the failure to adhere strictly to the WHO-recommended discriminating doses might be due to logistical constraints, such as the unavailability of insecticide-impregnated papers at the time of the trial. This practical impediment necessitated a pragmatic approach where alternative doses were explored. Therefore, the methodology section should clearly state the reasons for the deviation from the recommended doses.

***Response:* Dear, Reviewer, thank you very much again for your relevant suggestion. The statements "However, we did not follow the World Health Organization recommended discriminating doses for *Aedes* species due to unavailability of insecticide-impregnated papers at the time of the experiment. This practical obstacle required a strategy that looked into the alternative doses for the insecticides" have been added in the revised manuscript on page 6, Line 14-18.**

**Reviewer #2**

I still noticed many grammatical errors and some typos, and have mentioned them in red fonts at some places in the PDF file to draw attention of the authors to carefully check the text.

1. P1, L18 Typos correction to Non-blood fed

   ***Response:* Dear Reviewer, thank you very much again for your important indication on grammatical and typing errors. Thus, "Non-blood fed" has been changed to "Non-blood-fed" in the revised manuscript.**

2. P1, L19 "pyrethroids", "carbamates"

***Response:*** **"pyrethroids", "carbamates" have been corrected to "pyrethroid" and ca rbamate in the revised manuscript.**

**3.** P1, L25 "were" and L26, "were"

***Response*****: were, were, have been corrected to "was", "was" in the revised manuscript, because the statement explained about a single species i.e. *Ae. aegypti*.**

**4.** Residual P3, L19-20

***Response*****: "residual" has been corrected to "residual insecticide surface" in the revised manuscript.**

**5.** P 4, L2, "base line"

***Response*****: "base line" has been corrected to "baseline" in the revised manuscript.**

**6.** P7, delete the statement "The knockdown effect of pyrethroids against the *Ae. aegypti* were recorded during the 60 minutes exposure at an interval of 10 minutes from the start."

***Response*****: "The knockdown effect of pyrethroids against the *Ae. aegypti* were recorded during the 60 minutes exposure at an interval of 10 minutes from the start" has been removed because of redundancy with the phenotypic susceptibility test section.**

7. P8, L3, $KDT_{50}$ 95% [CI] and $KTD_{95}$ 95%, include the 95% inside the close bracket

***Response*****: $KDT_{50}$ 95% [CI] and $KDT9_{5}$ 95% [CI] have been corrected to $KDT_{50}$ [95%CI] and $KDT9_{5}$ [95%CI] in the revised manuscript.**

8. P8, L8, "Correct (figs 2-4) to Fig."

***Response*****: (figs 2-4) has been corrected to (Figs 2-4). Only the first letter should be capitalized and no dot was needed as PLOS ONE Journal guideline for in text citation of multiple figures.**

9. P9, L4, "resistance"

***Response*****: "resistance" has been corrected to "to be resistant" in the revised manuscript.**

10. P11, L7, "World health organization"

***Response*****: "World health organization" has been corrected to "World Health Organization" in the revised manuscript.**

11. P11, L10, "Add reference":

***Response*: The reference WHO, 2022 has been added in the revised manuscript.**

12. P11, L11, “Mortally”

    ***Response*: “mortally” has been corrected to “mortality” in the revised manuscript.**

13. P Add “%” to the insecticides in all figures 2-4

    ***Response*: “%” has been added to the insecticides in all figures of 2-4 in the revised manuscript.**
