# Supplementary material for: Susceptibility status of Aedes aegypti (Diptera: Culicidae) to public health insecticides in Southern Afar Region, Ethiopia
Source: PLoS One. 2024 Aug 23;19(8):e0309335. doi: 10.1371/journal.pone.0309335 (PMC11343450; doi:10.1371/journal.pone.0309335)
Supplement: Attachment — Submitted filename: Reponses to the reviewers.docx [file pone.0309335.s006.docx]

**Date 29/04/2024**

**To: James Colborn**

**Academic editor**

**PLOS ONE Journal**

**Sub: Providing point-by-point responses to the Editor’s and reviewers’ comments, questions and suggestions**

Dear James Colborn (Dr./Prof), we are grateful to the journal **PLOS ONE** for considering our original research manuscript entitled **Susceptibility status of *Aedes aegypti* (Diptera: Culicidae) to public health insecticides in Southern Afar region, Ethiopia** with the manuscript number of PONE-D-24-03915 for possible publication. We also appreciated the academic editor and reviewers to their relevant comments, questions and suggestions. The comments, questions and suggestions forwarded/given are highly helpful and valid towards the improvement of the manuscript. We present point-by-point responses to the editor and reviewers’ questions, comments and suggestions below.

**Comments on journal requirements**

1. Follow the PLOS ONE Journal submission guidelines.

***Response***: **We have followed PLOS ONE Journal** **submission guidelines throughout the revised manuscript.**

2. Funding information:

***Response*: we have provided the funding information. The statement “the study was financially supported by Addis Ababa University the Vice President for Research and Technology Transfer Office through its thematic research grant” has been included. Unfortunately, we did not include the funding statement in the submission system before. Thus, we kindly request you to incorporate the funding statement on the online submission system.**

3. Non-author contact information:

***Response*: To ensure guarantee for the long term stability and availability of the data, we provided the data used as supporting information file 1, supporting information file 2 and supporting information file 3. Supporting information file 1 shows measurement used to estimate duration in minutes in which KDT_50_ and KDT_95_ *Aedes aegypti* knocked-down and the 95% confidence interval were analyzed (Table 1 in the manuscript). Supporting information file 2 presents mortality values of *Ae. aegypti* used to build the percentage mortality graphs (graphs 2-4 in the manuscript). Supporting information file 3 reveals representative GPS points of the study sites recorded during *Ae. aegypti* larvae and pupae collection. We mapped the study areas using Ethio-region GIS shape files to show the location of the study sites. We did not use map image for analysis in the result section of the manuscript.**

4. Figure 1 copy-right:

***Response:* Authors have mapped the study area (named as Figure 1 in the manuscript) using arc GIS software version 10.8 from GIS Shape files of Ethiopia freely and no individual or organization involved. Thus, there is no copy-right issue for this figure.**

**Additional Editor’s comments**

All reviewers in felt that the manuscript lacked sufficient detail to evaluate. In particular they requested more detail on the methodology including the sampling methods and statistical analyses performed. They have also listed additional specific changes that should be addressed prior to resubmission.

***Response*: We have provided details on each section as per the comments given by the reviewers. We have made slight modification where necessary in the revised manuscript.**

**Reviewer #1**

**Major comments**

1. What criteria underlie the selection of insecticides in this study, specifically the inclusion of three pyrethroids and two carbamates? Additionally, are these insecticides currently in use for public health purposes?

***Response:* Viral diseases outbreaks are continuously reported in Ethiopia including the present study areas. Insecticide based viral diseases transmitting mosquito control is a quick responses during outbreaks. Thus, “the insecticides were selected for the study because they have been used for the control of mosquitoes of public health importance in Ethiopia and the study area”. “The insecticides are also among the insecticide classes used in the country”. The statements have been included in the methods (phenotypic susceptibility of *Aedes aegypti*) sub-section of revised manuscript. Yes, most are being used depending on the local transmission situation.**

2. According to the WHO guideline cited in the methodology section, the recommended discriminating doses for susceptibility tests of *Aedes* mosquitoes using pyrethroids are 0.25% Permethrin, 0.03% alpha-cypermethrin, and 0.03% deltamethrin. However, it appears that different discriminating doses of pyrethroids were used for the study. Why it is different? ***Response*: Thank you for the important question. The insecticides are used for public health in the study areas in particular and in Ethiopia in general with the same concentration of the present study tests. Thus, we used concentrations of the insecticides, WHO criteria, used to evaluate insecticide susceptibility status of *Anopheles arabiensis.* Furthermore, studies from Tanzania and Burkina Faso were used similar concentration of insecticides to test *Aedes aegypti* susceptibility to insecticides** (Kahamba et al., 2020; Namountougou et al., 2020)**.**
3. Why only phenotypic resistance studied? Was there a specific reason for not investigating the resistance mechanisms at play in the mosquito populations?

***Responses*: We share the comment as a limitation. We were not able to detect resistant genes to bendiocarb and propoxur due to lack of the reagents. We recommend further molecular investigations regarding the resistant genes.**

4. How did you identified the *Aedes* mosquitoes used for the susceptibility tests were all *Ae. Aegypti*?

***Responses*: Experienced personnel were carried out the collection and insecticide susceptibility tests. Selected numbers of the mosquitoes were identified before the test as *Ae. aegypti*. All the mosquitoes were checked for being *Ae. aegypti* at the end of each experiment. All the mosquitoes were identified as *Ae. aegypti*.**

5. How and why was the association between pyrethroid resistance in the study area and agricultural practices established?

***Responses*: Pyrethroids are mainly used in the control of mosquitoes that transmit viral diseases and malaria. They are also used in the control of agricultural pests (Shafer et al., 2008; Chrustek et al., 2018). It is known that insecticides used in agricultural practices induce public health insects to develop resistance against public health insecticides. Because some insecticides used in agricultural insects share a similar mechanism of action and bio-active ingredients with public health insecticides. In the study areas, farmers used the pyrethroids to control pests of crops. As a result, uses of the insecticides in agriculture might potentially contribute to resistant mosquitoes.**

**Miner comments**

1. Are *Ae. aegypti* and *Ae. bromeliae* the only two species these the only two *Aedes* species existing in the country? For example *Ae. vittaus* and *Ae. africanus* also exist in the country.

***Responses*: Thank you for your relevant comment. The statement “In Ethiopia, *Ae.* *aegypti, Ae. bromeliae,* *Ae. vittatus*, *Ae. hirsutus, Ae. simpsoni* complex and *Ae. africanus* are implicated as the potential vectors of viral diseases*”* has been included in the revised manuscript as suggested.**

2. Why the Knockdown effect of permethrin and alpha-cypermethrin not elaborated like that of deltamethrin?

***Responses*: The statement “On the other, *Ae. aegypti* exposed to permethrin showed the lowest KDT_50_ in Awash Arba”, has been included in the revised manuscript as commented.**

3. The vertical axis maximum value should not exceed 100 (Fig 2). This works for all graphs.

***Responses*: We have corrected the vertical axis of all the graphs to 100 in the revised manuscript as suggested.**

4. The susceptibility test of alpha-cypermethrin in Awash Arba town repeated or not?

***Response*: Yes, the test was repeated. The statement “*Ae. aegypti* susceptibility test was repeated for alpha-cypermethrin in Awash Arba town since *Ae. aegypti* populations were appeared to be suspected phenotypic resistance to this insecticide” has been included in the revised manuscript.**

5. Why is integrated vector control strategies recommended without supporting data from the study findings?

***Responses*: Thank you for the relevant question. We modified the conclusion based on the findings of the study in the revised manuscript.**

6. How are pyrethroids currently utilized in the control of vectors?

***Response*: Pyrethroids are utilized currently to control mosquito vectors in the form of long lasting insecticides treated nets or in the insecticide residual spray. Moreover, recently the mosquito vector emerge resistance to pyrethroids. Thus, potential new approaches existing pyrethroids are emerging such as pyrethroids plus PBO (pyrethroid synergist piperonyl butoxide) which are used to break the pyrethroid resistance by blocking metabolic resistance. The PBO are not acting as insecticides but they facilitate the action of the pyrethroids.**

Suggestions:

***Response*: All the suggestions given have been included in the revised manuscript.**

**Reviewer #2**

I suggest improvement in the manuscript and offer the following comments and suggestions:
Overall: correct numerous typos and improve English language; ensure the references cited in the text match with those in the References list.

***Response*: The grammar and typos have been checked and corrected in the revised the manuscript. Similarly, all references cited in the text have been checked their correctness in the reference list.**
Abstract:
- Background: In the last sentence of the “Background”, mention names of the insecticides instead of stating “public health insecticides”.

***Response*: “deltamethrin, permethrin, alpha-cypermethrin, pirimiphos-methyl, bendiocarb and propoxur” have been included in the revised manuscript in the last sentence of Abstract’s background.**
 -Methodology: Modify the sentence “Mortality was recorded after 24 hours” to “The mortality in mosquitoes was recorded 24 hours after 60 minutes of exposure”. Delete the abbreviation “KDT” for knockdown rate since later you have used KDT for the knockdown time.

***Response*:** **“The abbreviation “KDT” has been deleted.** **The mortality in mosquitoes was recorded 24 hours after 60 minutes of exposure” has been added in revised manuscript as suggested.**
 Results: In this sentence KDT95 implies Knockdown time, so modify the sentence to “*Aedes aegypti* from Awash Sebat showed high knock down time (KDT_95_ 34.01 minutes) to deltamethrin ….”

***Response*:** **“Deltamethrin in *Ae. aegypti* showed high knockdown time (KDT_95_ 34.01 minutes) in Awash Sebat as compared to other towns has been rephrased.**
-Results: Rename “alphacypermethrin” to “alpha-cypermethrin”, “delthamethrin” to “deltamethrin” and “pirimiphos methyl” to “pirimiphos-methyl” throughout the manuscript (in text, figures, and tables).

***Response*: “alphacypermethrin” to “alpha‑cypermethrin”, “delthamethrin” to “deltamethrin”, “pirimiphosmethyl” to “pirimiphos-methyl” have been renamed in the entire revised manuscript sections as suggested.**
- Conclusion: delete “control” at the end of the second sentence. Mention the insecticide discriminating concentration for each insecticide impregnated paper used. Also state that further studies are required to confirm resistance to alpha-cypermethrin.

***Response*:** **The word control has been deleted from the conclusion. In addition, the statement “However, further studies should be carried out to evaluate the susceptibility of *Ae. aegypti* to alpha-cypermethrin insecticide in the Awash Arba area”, has been included in the revised manuscript.**
-Introduction
-Correct typos: change “*Ae. albopictus*[11, 12].” to “*Ae. albopictus* [11, 12].”, “*Ae .aegypti*” to “*Ae. aegypti*”, “Fenitrothion” to “fenitrothion”.

***Response*: “*Ae. albopictus*[11, 12],” has been corrected to “*Ae. albopictus* [11, 12].” *Ae .aegypti*” to “*Ae. aegypti*”, “Fenitrothion” to “fenitrothion”, in the revised the manuscript as suggested.**
-Material and Methods
Study sites: Correct many typos (e.g. “820masl” to “820 masl” and so on.

***Response*: “820masl” to “820 masl”, “22.6^o^C to 30.6^o^C” to “22.6 ^o^C to 30.6 ^o^C” and “606.6mm” to “606.6 mm” have been corrected in the revised manuscript.**
 Exactly what insecticide spraying is practiced in the study areas for the control of malaria and arboviral diseases to give an indication of insecticidal interventions used.

***Response*: The statement “Insecticide such as deltamethrin, permethrin, alpha-cypermethrin, pirimiphos-methyl bendiocarb and propoxur are used,” has been included in the revised version of the manuscript.**
 Figure 1 is not mentioned in the text under the study sites.

***Response*: Fig 1, has been cited in the text in the revised manuscript.**
Insecticide susceptibility:
- Reference # 16 attributed to the WHO procedure is incorrect. Please recheck correctness of all references used in the text.

***Response*: All the references have been checked and reference number 16 has been corrected by reference 20 in the revised manuscript.**
- Correct numerous typos such as “delta-methrin” to “deltamethrin”.

***Response*: delta-methrin” has been corrected to “deltamethrin in the revised manuscript.**
- A correct number of mosquitoes (100) were exposed in the treatments but not in the control (40 instead of 50). This is a slight deviation from the WHO tube test procedure.

***Response*:** **We followed the WHO 2016 insecticide susceptibility test and exposed the control mosquitoes following the procedures in that manual.**
- “fed on 10% sugar solution” is an incorrect statement; instead, state “were offered cotton wool pads soaked in 10% sugar solution”.

***Response:*** **the statement “were offered cotton wool pads soaked in 10% sugar solution” has been corrected in the revised manuscript as suggested**.
Data analysis
 Correct this statement “suspected for resistance were with a mortality 90-97%” to “suspected of resistance were with a mortality of 90–97%”.

***Response:*** **the statement “suspected of resistance were with a mortality of 90–97%” has been added in the revised manuscript.**
 Reference # 34 is incorrect; the correct reference should be: WHO (2022). Manual for monitoring insecticide resistance in mosquito vectors and selecting appropriate interventions. <https://iris.who.int/handle/10665/356964>.

***Response*: Since we initially used WHO (2016). Monitoring and managing insecticide resistance in *Aedes* mosquito populations: interim guidance for entomologists*.* Thus reference # 34 has been corrected by reference 20.**
 -Correct the abbreviations “50% (KD50) and 95% (KD95)” to “50% (KDT50) and 95% (KDT95).

***Response:*** **The abbreviations “50% (KD50) and 95% (KD95)” have been corrected to “50% (KDT_50_) and 95% (KDT_95_) in the revised manuscript as suggested.**
-Results
-In Table 1 header, change “KDT50±SE” to “KDT50 ± SE” and “KDT95±SE” to “KDT95 ± SE”

***Response:*** **We have used estimated “KDT50 “KDT95 with their 95% confidence interval in the revised manuscript.**
- Correct numerous typos in the text and figures.

***Response*: we have edited typos in the text and corrected the vertical axis of the figures in the revised manuscript.**
-Discussion:
 -Is there any publication supporting the following statement “*Ae. aegypti* is generally considered as exophilic, exophagic …....”.

***Response:* reference number 37 cited in this study support the high exophilic and exophagic-ness of Ae*. aegypti.***
- While KDT95 for deltamethrin in one town was higher, the tube test does confirm full susceptibility of *Ae. aegypti*.

***Response:* The KDT_95_ for deltamethrin in Awash Sebat higher as compared to other pyrethroids in the study. Thus, it did not necessarily confirm resistance or suspect resistance since the mortality of *Ae. aegypti* were determined after 24 hrs.**
- Some of the WHO insecticide discriminating concentrations for *Aedes aegypti* adults have changed in 2022 following the publication of the above said WHO manual based on a WHO multi-centre study in which the authors’ institution was a collaborator, as follows:
o deltamethrin 0.05% to 0.03%
o permethrin 0.75% to 0.40%
o bendiocarb 0.1% to 0.2%
o pirimiphos-methyl 0.25% to 60 mg/m2
o alpha-cypermethrin 0.05% : no change
o propoxur 0.1% : no change
-Since *Ae. aegypti* was found resistant to bendiocarb 0.1% concentration while its concentration has been increased to 0.2%, discuss that further tests should be conducted with 0.2% impregnated papers to confirm resistance.

***Response:*** **the statements “World health organization in 2022 has recommended that the insecticide discriminating dose of bendiocarb for *Ae. aegypti* is changed from its initial 0.1% to 0.2% [45]. Thus, future efficacy studies of bendiocarb should target the 0.2% concentration”, have been added in the revised manuscript.**
References
- I did not check correctness of each reference cited, but I noticed at least two incorrect references, therefore authors should recheck all references cited.

***Response:* All the references cited in the text have been checked to their correctness in the reference list.**

**Reviewer #3:**

The authors have a good reason for conducting this research, the manuscript is well organized and written clearly enough to be accessible to non-specialists. However, lacks a detailed description of the method used to collect larvae and pupae.

It is understood that they collected in three places in the South of the Afar region of Ethiopia, where vector-transmitted infections occur, and that they did so from July to October 2022. However, they do not specify how many times each of the locations were visited during that 3 to 4 months for the collections to obtain a variety of samples, nor how many breeding sites they found from where they collected the specimens.

***Response*: The statements “A cross-sectional study was designed to collect larvae and pupae of *Aedes* species from July to October, 2022 in the Awash Sebat, Awash Arba and Werer towns. The study towns were selected purposively in collaboration with the local district health professionals and on the basis of the recent outbreak reports of dengue fever and chikungunya virus. The towns were surveyed once per month”, have been included in the revised manuscript.**

The latter is important because it is not known if the offspring they collected come from the same progeny or from few of them, hence the importance of collecting in different breeding sites and at different collection times in the same place. The material collected on the same day at various breeding sites of a locality must be combined so that there is mixing of the genes and thus the results of resistance to insecticides may correspond to several parental mosquitoes originated from the collected site. It is important to describe the number of samples collected on the dates in each of the locations. If possible, describe the number of larvae and pupae collected or an approximate number.

**Response: The objective of the study was to collect sufficient number of *Ae. aegypti* larvae and pupae for the susceptibility test. We did not record habitat characteristics, counts of larvae and pupae.**
By other hand, it is not specified how many experiments were carried out for the tests with each insecticide. It is understood that only 100 mosquitoes were used and 40 for two control tubes, so that makes only a test. However, at least two other replicates of collections separated in time must be carried out for the same collection location. In this way you can work with an average mortality for more precise data on the locality that is being collected, and if differences in mortalities are found in the three collections could be a good motive for discussion in this manuscript to enrich the discussion.

***Response:* Thank you for your relevant suggestion. The tests were performed in replicates (five treatment and two control replicates) for each insecticide as per the World Health Organization 2016 criteria. We believe that the results obtained are sufficiently enough to support the conclusion drawn in the study.**
-Discussion, 3rd paragraph, at the end: referring to the phrase "the findings observed in the present study were lower than results reported in Sudan, Tanzania and Nigeria" it would be good to add if those from Sudan, Tanzania and Nigeria were reported as resistant or only tolerants to deltamethrin, and maybe is good to mention their KDT50 and 95 values, so we can have an idea for comparison.

***Response:*** **Comparison of KDT_50_ and KDT_95_ values of deltamethrin to the study carried out in Sudan, Nigeria and Tanzania has been made. The statement “Overall the tube test revealed that *Ae. aegypti* were susceptible to deltamethrin in Sudan, Tanzania but suspected resistance in Nigeria,” has been included at the end of discussion section 3^rd^ paragraph in the revised manuscript.**

A sentence could be added in this section about the resistance mechanisms that could be present in the mosquitoes detected resistant to propoxur and bendiocarb, but not to organophosphate. It is known that the two groups of insecticides share the same site of action, and that if resistance to both groups has been diagnosed, such a situation could explain a resistance based on a point mutation in the site of action, however this is not the case.

***Response:* The statement “In addition, *Ae. aegpti* resistance to propoxur and bendiocarb may occur as a result of mutation in the insecticide target site enzyme Acetyl-cholinesterase (AChE) as reported in Cuba,” has been included in the revised manuscript.**

Minor corrections:
Introduction, 3rd paragraph. "Fenitrothion" change to "fenitrothion"

**Response: “Fenitrothion" has been changed to "fenitrothion" in the revised manuscript.**
Material and Methods: 1st paragraph. "Were" change to "Werer"

**Response:** "**Were" has been corrected to "Werer" in the revised manuscript.**
Insecticide susceptibility paragraph: "delta-methrin"change to "deltamethrin" and "bendio carb" change to bendiocarb"

**Response: delta-methrin" has been changed to "deltamethrin" and "bendio carb" has been changed to bendiocarb" in the revised manuscript.**
-Results, 1st paragraph, Table 1. "*Ae. Aegypti*" change to "*Ae. aegypti*"

***Response***:**"*Ae. Aegypti*" has been changed to "*Ae. aegypti*" in the revised manuscript**
Discussion, 4th paragraph. "propxur" change to "propoxur"

***Response*:** **propxur" has been change to "propoxur" in the revised manuscript**
Conclusion. "perimethrin" change to "permethrin"

***Response***: "**perimethrin" has been changed to "permethrin" in the revised manuscript**.
Check the double spaces between words throughout the writing and correct them to one space.

***Response***: **We have made the manuscript text** **double space while one space between words in the revised manuscript.**

**Reference**

Kahamba NF, et al. Habitat characteristics and insecticide susceptibility of *Aedes aegypti* in the Ifakara area, south-eastern Tanzania. Parasit Vectors. 2020; 13(1) 1-15.

Namountougou M, et al. Monitoring Insecticide Susceptibility in Aedes Aegypti Populations from the Two Biggest Cities, Ouagadougou and Bobo-Dioulasso, in Burkina Faso: Implication of Metabolic Resistance. Trop. Med. Infect. Dis. 2020; 5(84):1-9.

Shafer TJ, Rijal SO, Gross GW. Complete inhibition of spontaneous activity in neuronal networks in vitro by deltamethrin and permethrin. *Neuro Toxicology.* 2008;29:20‑212.

Chrustek A, Hołyńska-Iwan I, Dziembowska I,  Bogusiewicz J,  Wróblewski M, Cwynar A. et al. Current Research on the Safety of Pyrethroids Used as Insecticides. 2018; 54(4): 61
