# Supplementary material for: Susceptibility status of Aedes aegypti (Diptera: Culicidae) to public health insecticides in Southern Afar Region, Ethiopia
Source: PLoS One. 2024 Aug 23;19(8):e0309335. doi: 10.1371/journal.pone.0309335 (PMC11343450; doi:10.1371/journal.pone.0309335)
Supplement: Attachment — Submitted filename: Reponses to the reviewers.docx [file pone.0309335.s008.docx]

**Date 28/05/2024**

**To: James Colborn**

**Academic editor**

**PLOS ONE Journal**

**Sub: Providing point-by-point responses to the Editor’s and reviewers’ comments, questions and suggestions**

Dear James Colborn (Dr./Prof), we are grateful again to the journal **PLOS ONE** for considering our original research manuscript entitled **Susceptibility status of *Aedes aegypti* (Diptera: Culicidae) to public health insecticides in Southern Afar region, Ethiopia** with the manuscript number of PONE-D-24-03915R1 for possible publication. We also appreciated the academic editor and reviewers to their relevant comments and suggestions in the second reviews of the manuscript. The comments and suggestions forwarded are highly valuable to improve the manuscript. We present point-by-point responses to the editor and reviewers’ questions, comments and suggestions below.

**Comments on journal requirements**

1.Please review your reference list to ensure that it is complete and correct. If you have cited papers that have been retracted, please include the rationale for doing so in the manuscript text, or remove these references and replace them with relevant current references. Any changes to the reference list should be mentioned in the rebuttal letter that accompanies your revised manuscript. If you need to cite a retracted article, indicate the article’s retracted status in the References list and also include a citation and full reference for the retraction notice.

***Response:* Dear Editor, thank you very much once again for your relevant suggestion. We have reviewed the references cited in the text to their correctness in the references list. We have also corrected a few typos of existed references particularly the volume and issue numbers in the revised manuscript. Retracted references have not been used. We have cited the WHO, 2022 of reference number[45] on future recommendation for efficacy studies of bendiocarb that should target to the 0.2% concentration.**

2. While revising your submission, please upload your figure files to the Preflight Analysis and Conversion Engine (PACE) digital diagnostic tool.

***Response*: We have uploaded the figures to PACE to ensure that the figures meet PLOS requirements.**

**Reviewer #1**

The authors have responded to numerous comments, but there is still a gap in explaining why the recommended discriminating doses for susceptibility tests of *Aedes* mosquitoes, as outlined in the WHO guideline cited in the methodology section, were not followed. This discrepancy must be addressed transparently in the methodology, providing genuine explanations for the deviation. The authors may suggest that the failure to adhere strictly to the WHO-recommended discriminating doses might be due to logistical constraints, such as the unavailability of insecticide-impregnated papers at the time of the trial. This practical impediment necessitated a pragmatic approach where alternative doses were explored. Therefore, the methodology section should clearly state the reasons for the deviation from the recommended doses.

***Response:* Dear, Reviewer, thank you very much again for your relevant suggestion. The statements “However, we did not follow the World Health Organization recommended discriminating doses for *Aedes* species due to unavailability of insecticide-impregnated papers at the time of the experiment. This practical obstacle required a strategy that looked into the alternative doses for the insecticides” have been added in the revised manuscript on page 6, Line 14-18.**

**Reviewer #2**

 I still noticed many grammatical errors and some typos, and have mentioned them in red fonts at some places in the PDF file to draw attention of the authors to carefully check the text.

1. P1, L18 Typos correction to Non-blood fed

***Response:* Dear Reviewer, thank you very much again for your important indication on grammatical and typing errors. Thus, “Non-blood fed” has been changed to “Non-blood-fed” in the revised manuscript.**

2. P1, L19 “pyrethroids”, “carbamates”

***Response:* “pyrethroids”, “carbamates” have been corrected to “pyrethroid” and carbamate in the revised manuscript.**

3. P1, L25 “were” and L26, “were”

***Response*: were, were, have been corrected to “was”, “was” in the revised manuscript, because the statement explained about a single species i.e. *Ae. aegypti*.**

4. Residual P3, L19-20

***Response*: “residual” has been corrected to “residual insecticide surface” in the revised manuscript.**

5. P 4, L2, “base line”

***Response*: “base line” has been corrected to “baseline” in the revised manuscript.**

6. P7, delete the statement “The knockdown effect of pyrethroids against the *Ae. aegypti* were recorded during the 60 minutes exposure at an interval of 10 minutes from the start.”

***Response*: “The knockdown effect of pyrethroids against the *Ae. aegypti* were recorded during the 60 minutes exposure at an interval of 10 minutes from the start” has been removed because of redundancy with the phenotypic susceptibility test section.**

7. P8, L3, KDT_50_ 95% [CI] and KTD_95_ 95%, include the 95% inside the close bracket

***Response*: KDT_50_ 95% [CI] and KDT9_5_ 95% [CI] have been corrected to KDT_50_ [95%CI] and KDT9_5_ [95%CI] in the revised manuscript.**

8. P8, L8, “Correct (figs 2-4) to Fig.”

***Response*: (figs 2-4) has been corrected to (Figs 2-4). Only the first letter should be capitalized and no dot was needed as PLOS ONE Journal guideline for in text citation of multiple figures.**

9. P9, L4, “resistance”

***Response*: “resistance” has been corrected to “to be resistant” in the revised manuscript.**

10. P11, L7, “World health organization”

***Response*: “World health organization” has been corrected to “World Health Organization” in the revised manuscript.**

11. P11, L10, “Add reference”:

***Response*: The reference WHO, 2022 has been added in the revised manuscript.**

12. P11, L11, “Mortally”

***Response*: “mortally” has been corrected to “mortality” in the revised manuscript.**

13. P Add “%” to the insecticides in all figures 2-4

***Response*: “%” has been added to the insecticides in all figures of 2-4 in the revised manuscript.**
