# Supplementary material for: Susceptibility status of Aedes aegypti (Diptera: Culicidae) to public health insecticides in Southern Afar Region, Ethiopia
Source: PLoS One. 2024 Aug 23;19(8):e0309335. doi: 10.1371/journal.pone.0309335 (PMC11343450; doi:10.1371/journal.pone.0309335)
Supplement: Attachment — Submitted filename: Comment.docx [file pone.0309335.s009.docx]

**Dear Editor**,

Thank you very much for your consistent efforts to improve the manuscript to meet the standards of your journal.

The authors have addressed my concerns, and I appreciate their thorough response. They have justified their use of the discrimination doses of insecticides according to WHO recommendations for testing insecticide susceptibility in *Aedes* mosquitoes. This indicates that they employed the recommended doses for testing *Anopheles* mosquitoes as outlined in the standard operating procedure for testing insecticide susceptibility in adult mosquitoes using WHO tube tests (WHO, 2022), rather than following the guidelines for monitoring and managing insecticide resistance in *Aedes* mosquito populations: Interim Guidance for Entomologists (WHO, 2016). Therefore, I suggest updating the reference accordingly.
