# Supplementary material for: Susceptibility status of Aedes aegypti (Diptera: Culicidae) to public health insecticides in Southern Afar Region, Ethiopia
Source: PLoS One. 2024 Aug 23;19(8):e0309335. doi: 10.1371/journal.pone.0309335 (PMC11343450; doi:10.1371/journal.pone.0309335)
Supplement: Attachment — Submitted filename: Reponses to the reviewers.docx [file pone.0309335.s011.docx]

**Date 24/06/2024**

**To: James Colborn**

**Academic editor**

**PLOS ONE Journal**

**Sub: Providing point-by-point responses to the Editor’s and reviewers’ comments, questions and suggestions**

Dear James Colborn (Dr./Prof), we are grateful again to You and the journal **PLOS ONE** for considering our original research manuscript entitled **Susceptibility status of *Aedes aegypti* (Diptera: Culicidae) to public health insecticides in Southern Afar region, Ethiopia** with the manuscript number of PONE-D-24-03915R2 for possible publication. We also appreciated the academic **Editor** and **Reviewers** to their helpful efforts in reviewing our manuscript in these series of revisions. We present point-by-point responses to the Editor and Reviewers’ questions, comments and suggestions below.

**Comments on journal requirements**

1. Please review your reference list to ensure that it is complete and correct. If you have cited papers that have been retracted, please include the rationale for doing so in the manuscript text, or remove these references and replace them with relevant current references. Any changes to the reference list should be mentioned in the rebuttal letter that accompanies your revised manuscript. If you need to cite a retracted article, indicate the article’s retracted status in the References list and also include a citation and full reference for the retraction notice.

***Response:* Dear Editor, We warmly thank You for Your valuable efforts to improve our manuscript to meet the PLOS ONE’s journal standard.**

**We have reviewed the references cited in the text to their correctness in the reference list. We have included the reference WHO 2022, number [****34****] on the Phenotypic susceptibility status of *Aedes aegypti* section of the revised manuscript as suggested by Reviewer #1. Retracted articles have not been used in the manuscript. We have also carefully checked the grammar and typos in the revised manuscript. We believed that the manuscript meet PLOS ONE’s publication standard.**

**Reviewer #1**

Reviewer #1: The authors have addressed my concerns, and I appreciate their thorough response. They have justified their use of the discrimination doses of insecticides according to WHO recommendations for testing insecticide susceptibility in *Aedes* mosquitoes. This indicates that they employed the recommended doses for testing *Anopheles* mosquitoes as outlined in the standard operating procedure for testing insecticide susceptibility in adult mosquitoes using WHO tube tests (WHO, 2022), rather than following the guidelines for monitoring and managing insecticide resistance in *Aedes* mosquito populations: Interim Guidance for Entomologists (WHO, 2016). Therefore, I suggest updating the reference accordingly.

***Response:* Dear Reviewer, We would like to thank you for your time and efforts you made to enhance the quality of our manuscript in these series of revisions.**

**We have updated the reference WHO 2022 [****34****]** **on the method section in the revised manuscript as suggested.**

**Reviewer #2**

I have inserted numerous corrections in the PDF file. Authors should carefully look at the suggested corrections to further improvise the manuscript. Additionally, two specific comments are as follows:

1. At the end of many lines, words are broken e.g. on page 1 lines 13, 14, 21, 24. Please correct these errors in the text throughout.

***Response:* Dear Reviewer, We highly appreciated you for your time and efforts that you made to enhance the quality of our manuscript in these series of revisions.**

**We have corrected the broken words throughout the revised manuscript as suggested. We have checked the grammar and correct typos in the revised manuscript.**

2. Page 10: line 6: *Aedes aegypti* is generally endophilic and endophilic whereas the authors have stated that they are generally exophilic and exophagic. Can they support this statement with any published information?

***Response:* Dear, Reviewer thank you again for the question.**

**We have re-phrased the statement*,* “*Aedes aegypti* is generally considered as exophilic and exophagic and daytime biters” as “*Aedes aegypti* is considered by some authors to be highly exophilic, exophagic, and a daytime biter”. The references number [****38****] and [****39****] cited in this study supported the high exophilic and exophagic-ness of Ae*. aegypti.* For instance, the authors in the reference 38 identified that 95.4% of blood-fed females of *Ae. aegypti formosus* subspecies were collected from outdoor sites whereas, only 4.6% were from indoor sites. In addition, 84.5% of the blood-fed females *Ae. aegypti aegypti* were collected from outdoor sites and 14.5 from the indoors. Thus, this suggested that the more exophilic/exophagic-ness of *Aedes aegypti* as a species.**
